# Spin and reporting quality in systematic reviews of nonrandomized studies in restorative dentistry: a meta-research study

**DOI:** 10.1590/1807-3107bor-2026.vol40.094

**Published:** 2026-11-09

**Authors:** Laylla GALDINO-SANTOS, Rokaia Ahmed ELAGAMI, Felipe de Araújo SENSEVER, Bas LOOMANS, Luiz Alexandre CHISINI, Tatiana PEREIRA-CENCI

**Affiliations:** (a) Universidade Federal de Pelotas – UFPel, Post-Graduate Program in Dentistry, Department of Restorative Dentistry, Pelotas, RS, Brazil.; (b) Universidade de São Paulo – USP, School of Dentistry, Department of Paediatric Dentistry, São Paulo, SP, Brazil.; (c) Radboud University Medical Center, Research Institute for Medical Innovation,Department of Dentistry, Nijmegen, The Netherlands.

**Keywords:** Bias, Systematic Review, Dental Research

## Abstract

To investigate the prevalence and types of spin, assess reporting quality and risk of bias, and identify factors associated with spin in systematic reviews with meta-analyses of nonrandomized studies in restorative dentistry. Five databases were searched through October 2025. Eligible systematic reviews compared at least two restorative materials or interventions for permanent teeth and reported restoration longevity, survival, or success/failure. Spin was assessed in abstracts and main texts using the classification by Yavchitz et al. Reporting quality and risk of bias were evaluated using AMSTAR 2 and ROBIS. Associations with spin were explored using Poisson and ordinal logistic regression. Twenty-six systematic reviews were included. Spin was identified in 92.3% of abstracts and 96.15% of main texts. In abstracts, the most frequent strategies were failure to declare conflicts of interest (34.61%) and failure to consider differences in study designs (34.61%). In the main text, common strategies included inadequate consideration of reporting bias (80.76%), insufficient consideration of risk of bias of primary studies (34.61%), and conclusions claiming equivalence despite nonsignificant results with wide confidence intervals (26.31%). Most reviews were rated as critically low quality by AMSTAR 2 (73.08%), and 38.46% had high risk of bias by ROBIS. Exceeding the abstract word limit (prevalence ratio [PR = 1.69; 95%CI: 1.19–2.42), failure to report funding sources (PR = 2.10; 95%CI: 1.22–3.59), and absence of a structured abstract (PR = 1.56; 95%CI: 1.18–2.07) were associated with spin. Spin is prevalent in systematic reviews of nonrandomized studies in restorative dentistry and is associated with inadequate reporting practices.

## Introduction

Spin refers to the distortion or misinterpretation of study results, whether intentional or not, in a way that presents findings as more favorable than is warranted by the available data.^1^ Evidence suggests that spin is frequently incorporated into the reporting and interpretation of clinical studies, leading to overestimation of the strength and certainty of the evidence.^2,3^ Such distorted reporting may misinform patients, clinicians, and policymakers and may ultimately influence healthcare decisions.^4^

In restorative dentistry, the longevity of restorations depends not only on the properties of materials and techniques but also on patient-related factors, such as caries risk and parafunctional habits, as well as operator skill.^5-7^ Despite this multifactorial nature, clinical research provides limited and heterogeneous evidence to guide optimal material selection, particularly when considering adverse events, costs, and patient preferences.^8,9^ In this context, spin may further mislead clinicians by overstating benefits or minimizing risks, potentially resulting in inappropriate clinical decisions.

The number of systematic reviews (SRs) in dentistry has increased substantially over recent decades.^9^ However, the limited availability of randomized controlled trials (RCTs) has led to the frequent inclusion of nonrandomized studies in evidence syntheses.^10,11^ Although RCTs remain the gold standard for evaluating treatment efficacy and safety,^11-13^ nonrandomized studies (NRs) play an important role in clinical research when randomized evidence is unavailable or impractical, despite their greater susceptibility to bias and confounding.^14,15^ Consequently, SRs including NRs have become increasingly influential in informing clinical practice guidelines.

Despite the central role of SRs in evidence-based dentistry, no previous study has systematically evaluated the prevalence of spin and reporting quality in SRs with meta-analyses of nonrandomized studies in operative dentistry. Building on prior meta-research conducted by the authors on randomized trials,^2^ the present study addresses this gap. Considering the inherent methodological limitations of nonrandomized studies, SRs synthesizing this evidence may be particularly vulnerable to interpretative bias and reporting distortion.^16-18^ Therefore, this study aimed to investigate the prevalence and types of spin in SRs with meta-analyses of nonrandomized studies in operative dentistry, to assess their reporting quality and risk of bias, and identify factors associated with spin.

## Methods

This SR followed the Preferred Reporting Items for Overviews of Reviews (PRIOR) guideline (Supplementary Material 1)^19^ and the reporting guidelines for meta-research studies,^20^ where applicable. The study protocol, data used in the analysis, and all supplementary materials (e.g., search strategies, assessment criteria, extracted data) are publicly available in the Open Science Framework (OSF) repository: https://osf.io/nkyfg. No deviations from the original protocol or amendments were made during the study.

### Eligibility criteria

SRs with meta-analyses of nonrandomized studies in operative dentistry were eligible if they compared at least two restorative materials or treatment modalities for permanent teeth. Included interventions comprised both direct (e.g., composite resin, amalgam) and indirect restorations (e.g., crowns, inlays, onlays, and posts) in anterior and posterior teeth. Reviews were required to report at least one of the following outcomes: restoration survival, success, or failure. Only reviews that analyzed nonrandomized studies separately from RCTs were included to avoid bias arising from combining study designs with distinct methodological characteristics and risks of bias.^21^ Studies involving primary dentition were excluded. No restrictions were applied regarding follow-up period, language, publication year, or geographic region.

### Literature search and study selection

A comprehensive literature search was conducted through October 2025 in PubMed/MEDLINE (via Elsevier), Scopus (via Elsevier), Web of Science (via Clarivate), and the Cochrane Library (via Wiley). Gray literature was searched using Google Scholar, with screening limited to the first 300 records retrieved. In addition, reference lists of included studies and relevant SRs were manually screened. Search strategies combined MeSH terms, keywords, and filters for SRs and were adapted for each database (Supplementary Material 2). Strategies were developed and peer-reviewed by two authors with experience in SRs. All records were imported into EndNote X9 for automatic and manual removal of duplicates.

Study selection was performed in two stages. First, two reviewers independently screened titles and abstracts using predefined eligibility criteria. Second, the same reviewers independently assessed the full texts of potentially eligible records. Disagreements were resolved by consensus or, when necessary, by consultation with a third reviewer. Inter-reviewer agreement was κ = 0.86. Reasons for full-text exclusion are provided in Supplementary Material 3 (https://osf.io/fj6x8/).

### Data collection process

Data were independently extracted and coded from all included SRs by two reviewers (LGS and RAE) using Microsoft Excel. Disagreements were resolved by discussion, and unresolved cases were adjudicated by a third reviewer (TPC).

Extracted variables were grouped into three domains: a) General review characteristics included the number of studies per meta-analysis, the total number of outcomes, and whether primary and secondary outcomes were explicitly defined. When not specified, the first outcome reported in the Results section was considered the primary outcome; b) Journal-related characteristics included abstract word count (classified according to compliance with journal limits), publishing continent based on the corresponding author affiliation, open-access status, and Scopus citation count at the time of data extraction, and c) Methodological and reporting characteristics comprised PRISMA adherence (assessed item by item as yes/no), protocol registration, funding and conflict of interest declarations (based on information reported at publication), use of a formal risk-of-bias assessment, and application of the GRADE approach. All variables were selected a priori based on previous meta-research indicating potential associations with reporting quality and bias (Supplementary Table 4).^2,22-26^

### Outcome assessment

Before data extraction, two reviewers (RAE and LGS) were trained and calibrated using the spin classification proposed by Yavchitz et al.^27^ Calibration involved independent assessment of five SRs not included in the final sample, with disagreements resolved by consensus with a third reviewer experienced in spin methodology (TPC). Following calibration, spin was independently assessed in duplicate by the same two reviewers for all included SRs, with disagreements resolved by discussion or consultation with a third reviewer.

The primary outcome was spin prevalence, evaluated as follows: a) presence of spin in abstracts; b) level of spin in the abstract; and c) presence of spin in the main text. Spin was classified according to Yavchitz et al.,^27^ and all items for abstracts and main texts are detailed in Table 1. Spin criteria were grouped into three categories: misleading reporting, misleading interpretation, and inappropriate extrapolation.

**Table 1:** Classification of spin in the main text and abstracts of systematic reviews and meta-analyses.

Main category	Description of spin in main text	Spin frequency, n = 26; n (%)	Description of spin in abstract	Spin frequency n (%)
Misleading reporting	(1) Failure to acknowledge a departure from protocol that could modify the interpretation of results	9 (34.6)	(1) Selective reporting of or overemphasis on efficacy outcomes favoring beneficial effects of the experimental intervention	2 (7.69)
	(2) Selective reporting of or overemphasis on efficacy outcomes favoring beneficial effects of the experimental intervention (e.g., secondary outcomes, subgroup analyses)	1 (3.84)	(2) Selective reporting of or overemphasis on harm outcomes favoring safety of the experimental intervention	1 (3.84)
	(3) Selective reporting of or overemphasis on harm outcomes favoring safety of the experimental intervention	0 (0)	(3) Failure to report a wide confidence interval of estimates	6 (23.07)
	(4) No or inadequate reporting of the limitations of the systematic review	4 (15.38)	(4) Failure to report conflicts of interest	9 (34.61)
	(5) Selective citation of articles in favor of the beneficial effect of the experimental intervention	1 (3.84)	(5) Inadequate focus on the results of primary studies favoring beneficial effects of the experimental intervention instead of the meta-analysis results	1 (3.84)
	(6) Failure to report conflicts of interest	8 (30.76)	(6) Conclusion focusing selectively on a statistically significant efficacy outcome	2 (7.69)
	(7) Conclusion focusing selectively on a statistically significant efficacy outcome	0 (0)	(7) Failure to report the number of studies/patients actually contributing to the analysis for main outcomes	5 (19.20)
	(8) Selective reporting of analysis favoring beneficial effects of the experimental intervention (e.g., selective analysis using random or fixed effect according to the results)	0 (0)	(8) Failure to specify the direction of the effect when it favors the control intervention	1 (3.84)
	(9) Inadequate focus on the results of primary studies favoring beneficial effects of the experimental intervention instead of the meta-analysis results	2 (7.69)		
	(10) Changing the scale of the forest plot to magnify the results (diamond size)	0 (0)		
Misleading interpretation	(11) Title claims or suggests a beneficial effect of the experimental intervention not supported by the findings	0 (0)	(9) Title claims or suggests a beneficial effect of the experimental intervention not supported by the findings	0 (0)
	(12) Inadequate interpretation of nonsignificant results (with a wide confidence interval) as a lack of effect or an equivalent effect for efficacy outcomes	1 (3.84)	(10) Inadequate focus on the p value instead of magnitude of the effect estimates for harm or efficacy outcome	0 (0)
	(13) Inadequate interpretation of nonsignificant results (with a wide confidence interval) as demonstrating safety for harm outcomes	1 (3.84)	(11) Focus on the relative effect when the absolute effect is small	0 (0)
	(14) Inadequate focus on the p value instead of magnitude of the effect estimates for harm or efficacy outcome	0 (0)	(12) Conclusion claiming equivalence or comparable effectiveness for nonsignificant results with a wide confidence interval	5 (19.23)
	(15) Focus on the relative effect when the absolute effect is small	0 (0)	(13) Conclusion formulating recommendations for clinical practice not supported by the findings	6 (23.07)
	(16) Misleading interpretation of cited articles, favoring the beneficial effect of the experimental intervention	1 (3.84)	(14) Conclusion claiming safety based on nonsignificant results with a wide confidence interval	0 (0)
	(17) Conclusion claiming equivalence or comparable effectiveness for nonsignificant results with a wide confidence interval	5 (19.23)	(15) Conclusion claiming the beneficial effect of the experimental treatment despite high risk of bias in primary studies	2 (7.69)
Misleading interpretation	(18) Conclusion formulating recommendations for clinical practice not supported by the findings	8 (30.76)	(16) Conclusion claiming the beneficial effect of the experimental treatment despite reporting bias	2 (7.69)
	(19) Conclusion claiming safety based on nonsignificant results with a wide confidence interval	1 (3.84)	(17) Conclusion claiming the beneficial effect of the experimental treatment despite high heterogeneity	5 (19.23)
	(20) Conclusion ignoring the high risk of bias of the studies, heterogeneity or reporting bias (i.e., a low level of evidence) in the interpretation of results	10 (38.46%)	(18) Ignoring that the review included different study designs (e.g., controlled trial or observational studies)	9 (34.61%)
	(21) No or inadequate consideration of heterogeneity in results interpretation (i.e., no assessment of heterogeneity reported, claims of absence of heterogeneity not supported by the data, claims of beneficial effect of the treatment despite high heterogeneity, no downgrading of evidence in cases of high heterogeneity, interpreting nonsignificant results for the test of heterogeneity as evidence of no heterogeneity)	6 (23.07)		
Inappropriate extrapolation	(22) No or inadequate consideration of the risk of bias of primary studies in results interpretation (i.e., no reporting of the risk of bias of primary studies, claims of low risk of bias not supported by the data, no downgrading of evidence despite several high risk of bias studies)	9 (34.61)		
	(23) No or inadequate consideration of reporting bias in results interpretation (i.e., no reporting of an assessment of reporting bias, claims of efficacy despite evidence of reporting bias, claims of absence of reporting bias not supported by the data, interpretation of a negative test result as absence of publication bias, use of a test without meeting validity conditions, inadequate interpretation of a funnel plot)	21 (80.76)		
	(24) Inadequate extrapolation of the results from a surrogate marker or specific outcomes to the global improvement of the disease	0 (0)	(19) Conclusion extrapolating review findings to a different population or setting	1 (3.84)
	(25) Inadequate extrapolation of results to a larger population, a larger setting, or a wider set of interventions (e.g., from a specific rehabilitation program to all rehabilitation programs, or from a specialized unit to a nonspecialized medical unit)	0 (0)	(20) Conclusion extrapolating review findings to a different intervention (i.e., claiming efficacy of one specific intervention although the review covers a class of interventions)	0 (0)
	(26) Conclusion extrapolating the review findings to a different population or setting	1 (3.84)	(21) Conclusion extrapolating review findings from a surrogate marker or specific outcomes to the global improvement of the disease	0 (0)
	(27) Conclusion extrapolating review findings to a different intervention (i.e., claiming efficacy of one specific intervention although the review covers a class of several interventions)	0 (0)		
	(28) Conclusion extrapolating review findings from a surrogate marker or a specific outcome to the global improvement of the disease	0 (0)		
Other spin strategies	Conclusion claiming superiority based on nonsignificant results without a direct meta-analysis of the primary outcome	2 (7.69)	Claims of clinical relevance regarding safety or recommendations for use not supported by the findings	1 (3.84)
			Conclusion claiming superiority without a meta-analysis of the primary outcome	1 (3.84)

Spin was assessed separately for abstracts and main texts. For abstracts, 21 spin items were evaluated, generating a count score ranging from 0 to 21. In addition, abstracts were classified according to spin severity as high (presence of at least one of the nine most severe spin items), low (spin present but without severe items), or no spin (Figure 1). When information was unclear, standardized interpretation rules defined during calibration were applied to ensure consistency and reproducibility.

**Figure 1:**
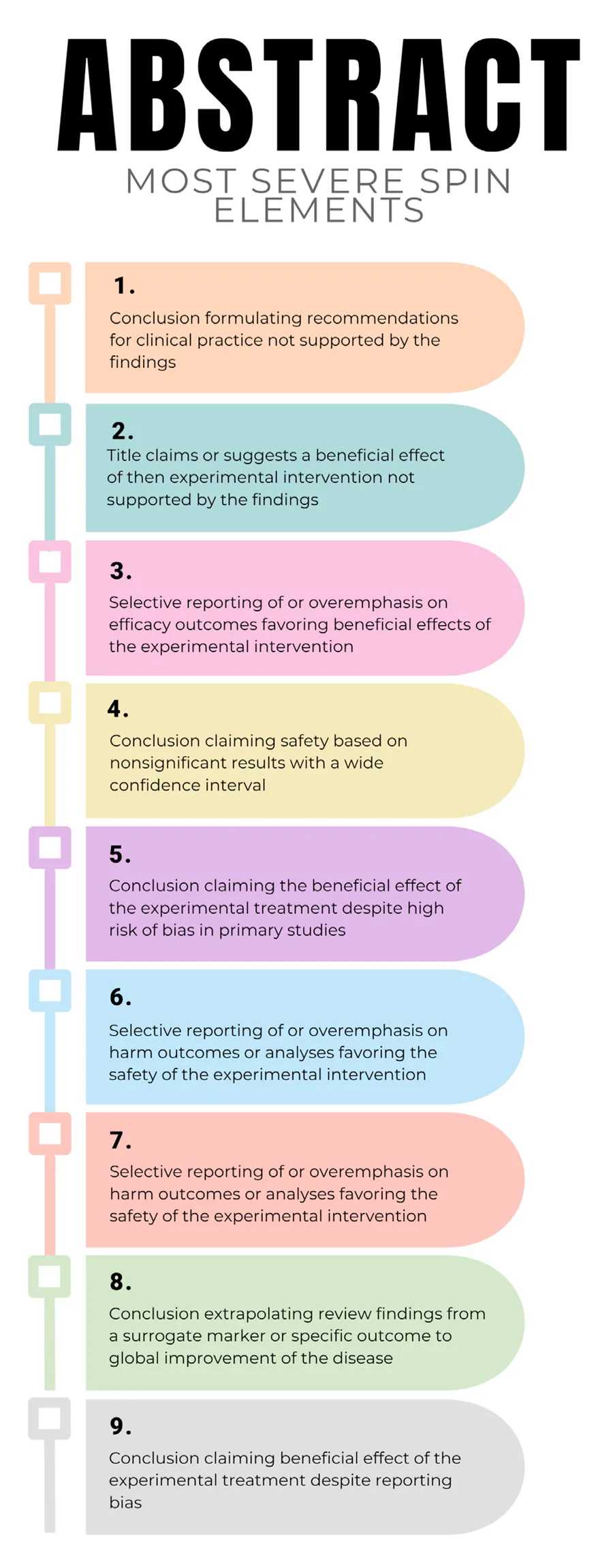
List of most severe types of spin in the abstract.

The original classification was applied without modification whenever possible. For criteria involving wide confidence intervals (CI), objective thresholds were adopted to reduce subjectivity: CIs were considered wide when the absolute difference between upper and lower bounds exceeded 1.0 for odds ratio or risk ratio estimates. Detailed results of abstract spin severity are provided in Supplementary Material 5.

#### Reporting quality

The same two reviewers were calibrated to ensure consistent and standardized assessment of the AMSTAR 2^28^ and ROBIS tools. For each tool, five SRs not included in the study sample were independently evaluated. Discrepancies were discussed in joint meetings, and consensus was reached with the support of a senior researcher (TPC) with prior experience using both tools. The methodological quality of the SRs was assessed using AMSTAR 2, a tool developed to evaluate the methodological quality of SRs that include RCTs or NRs. Each SR received a rating of high, moderate, low, or critically low based on its performance across various domains. Inter-reviewer agreement for AMSTAR 2 was calculated using Cohen’s κ, which was 0.81.

#### Methodology for assessing risk of bias

The risk of bias of the included SRs was assessed using the ROBIS tool, which is designed to evaluate the risk of bias in SRs^19^. ROBIS comprises four key phases: a) Establishment of Study Eligibility Criteria, b) Identification and Selection of Studies, c) Data Collection and Appraisal of Studies, and d) Synthesis of Findings. The assessment was independently performed by two reviewers trained in the tool, achieving almost perfect agreement (κ = 0.89). ROBIS includes signaling questions to assess potential concerns in the review process and the overall risk of bias,^29^ guiding evaluation of whether the review process, results, and conclusions may be biased.

## Data analysis

Quantitative variables were summarized as means and standard deviations, and categorical variables as frequencies and percentages. For exploratory purposes, year of publication was dichotomized at the sample median (2020), selected a priori based on the publication of the PRISMA 2020 update,^30^ allowing assessment of temporal patterns in reporting practices.

Poisson regression models were used to analyze count outcomes, with results expressed as prevalence ratios (PR) and 95%CI, representing relative differences in mean counts between groups. The level of spin (no, low, or high) was analyzed using ordinal logistic regression with robust standard errors, reflecting the ordered nature of the outcome. Variables associated with outcomes at p < 0.20 in univariate analyses were considered for inclusion in multivariable models, with final selection based on statistical significance and theoretical relevance.^31,32^ Given the exploratory nature of this meta-research, no formal adjustment for multiple comparisons was applied in the primary analyses. Bonferroni correction was performed as a sensitivity analysis to assess the robustness of the findings.^33,34^

Additionally, a descriptive comparative analysis was conducted to contextualize spin strategies identified in reviews of NRs against findings from SRs of RCTs reported by Sensever et al.^2^ This comparison—considered a protocol deviation—was based on absolute frequencies and percentages of comparable spin items. All analyses were performed using Stata 16.0, and data, code, and supplementary materials are publicly available in the Open Science Framework.

## Results

The initial search identified 10,175 unique articles, of which 3,135 were removed as duplicates. An additional 6,702 articles were excluded during the title and abstract screening. Full-text screening resulted in the exclusion of 312 articles. Thus, 25 SRs met the inclusion criteria. Additionally, one further record was identified through hand-searching, resulting in a total of 26 included reviews (Figure 2).^35-60^

**Figure 2:**
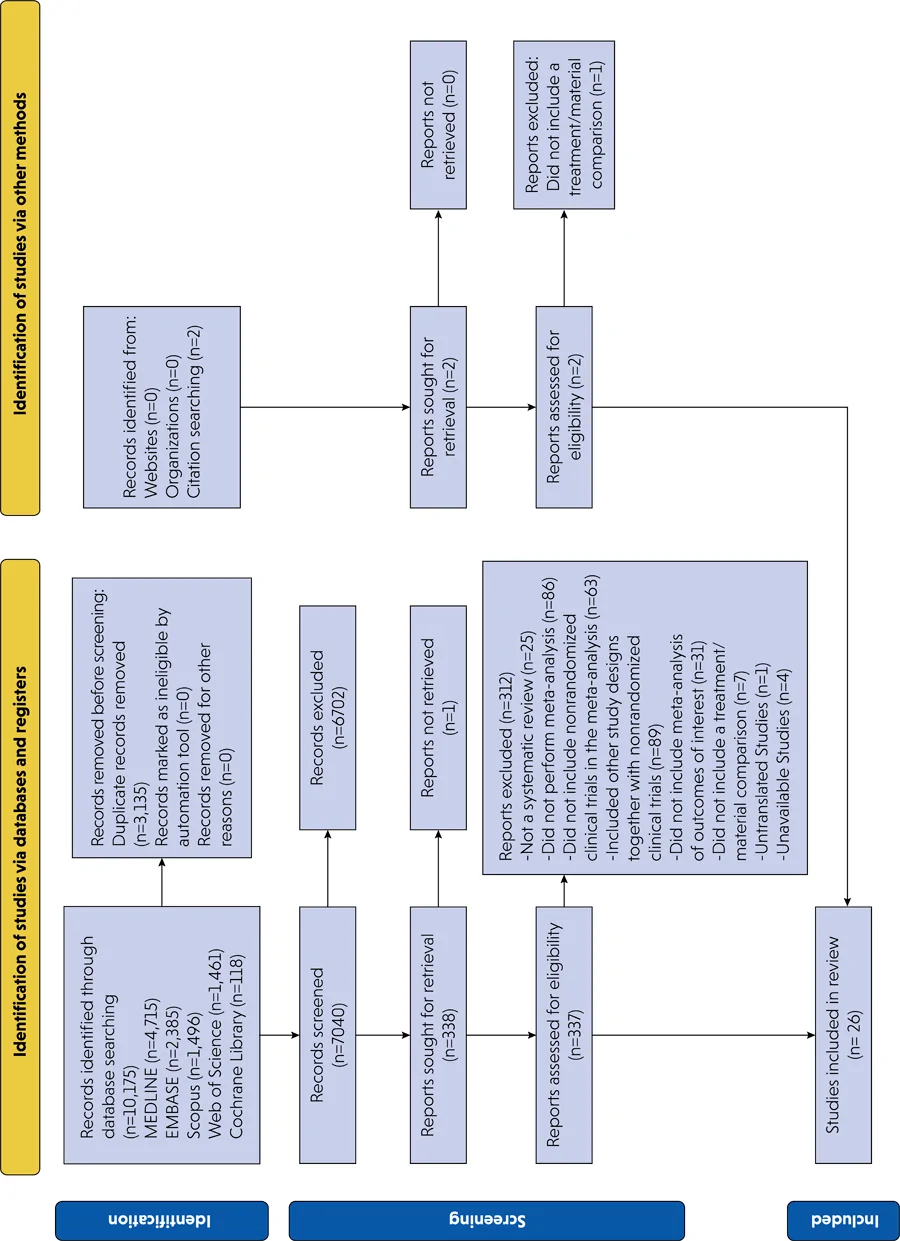
Flow diagram of the study selection.

Table 2 describes the included studies. The studies originated from 14 countries, with the majority from Europe (53.85%). The SRs were published in 14 different journals. Of these 25 studies, seven^46,48,49,52,58-60^ failed to use or report any standardized tool for assessing risk of bias or reporting quality. The mean number of primary studies included in the SRs was 18.34 (± 16.93), ranging from 3 to 62 studies. The mean journal impact factor was 10.5 (± 0.86), ranging from 1.3 to 5. Among the 26 included SRs, eleven (42.3%) synthesized data from more than one study design, specifically combining RCTs and NRs.^37-40,42,43,51,54,56,59,61^The NRs included both prospective and retrospective observational designs (retrospective, prospective studies, cohort studies, and case series).

**Table 2:** Characteristics of the included studies (n = 26).

Author (year)	Continent	Study design included in the SR	Risk of bias tool	Protocol registration	Number of primary outcomes	Number of secondary outcomes	Number of included primary studies	Conflict of interest reporting	Spin level in abstract	Number of spin items in abstract	Number of spin items in main text
Albanesi et al., 2016	America	Prospective and retrospective	Newcastle-Ottawa Scale (NOS)	CRD42015016606	1	0	8	No	Low	3	4
Al-Dabbagh et al., 2021	Asia	Prospective and retrospective	Newcastle-Ottawa Scale (NOS)	NP	1	3	3	No	Serious	3	3
Alenezi et al., 2024	Asia	Prospective and retrospective	NIH Quality Assessment Tool	NP	2	0	57	No	Low	1	4
Alqutaibi et al., 2024	Asia	RCT	Cochrane RoB 2 tool	CRD42021283002	1	1	12	Yes	Low	1	3
		NR	Newcastle-Ottawa Scale (NOS)								
Bhagwat et al., 2025	Asia	RCT	Newcastle-Ottawa Scale (NOS)	CRD42023469680	1	2	5	Yes	Serious	3	4
		NR	Cochrane RoB 2 tool								
Bresser et al., 2023	Europe	RCT	ROBINS-I	CRD42021233185	1	1	7	Yes	Serious	3	0
		NR									
Dewan et al., 2023	Asia	RCT/NR	Newcastle-Ottawa Scale (NOS)	NP	1	0	9	Yes	Low	1	7
Chantler et al., 2024	Europe	Prospective and retrospective	RoB 2	CRD42020173135	1	0	14	Yes	Serious	3	2
			Newcastle-Ottawa Scale (NOS)								
Dioguardi et al., 2021	Europe	Prospective and retrospective	Newcastle-Ottawa Scale (NOS)	CRD42020204095	1	1	6	Yes	No spin	0	0
Dioguardi et al., 2022	Europe	RCT/NR	Cochrane Handbook checklist	CRD42021268060	3	1	19	No	Low	3	1
Fan et al., 2025	Asia	RCT/NR	Newcastle-Ottawa Scale (NOS)	CRD42023397213	1	1	10	Yes	Low	3	2
		Case series	Joanna Briggs Institute (JBI) tool								
Fathi et al., 2022	Asia	Prospective	Newcastle-Ottawa Scale (NOS)	NP	1	1	9	No	Low	2	2
Heintze et al., 2022	Europe	RCT/NR	Cochrane Handbook checklist	NP	1	2	62	No	Low	3	4
Kassardjian et al., 2016	Europe	Prospective and retrospective	NI	NP	1	0	53	No	Low	1	4
Kreulen et al., 1998	Europe	Prospective and retrospective	Antczak et al., 1986	NP	1	0	15	No	Serious	5	6
Lim et al., 2023	Asia	RCT/NR	Newcastle-Ottawa Scale (NOS) RoB 2	CRD42022336857	2	0	7	Yes	Low	1	1
Mendes et al., 2022	America	Prospective and retrospective	ROBINS-I	CRD42021224970	1	0	3	Yes	Low	1	1
Pjetursson et al., 2004	Europe	Prospective and retrospective	NI	NP	2	0	21	Yes	Low	1	5
Pjetursson et al., 2007	Europe	Prospective and retrospective	NI	NP	1	2	34	No	Serious	5	5
Ploumaki et al., 2013	Europe	Prospective and retrospective	NI	NP	1	0	9	Yes	Serious	2	7
Sailer et al., 2007	Europe	Prospective and retrospective	NI	NP	1	1	9	No	Low	1	6
Sailer et al. 2018	Europe	RCT/NR	NI	CRD42017079072	2	0	19	Yes	Low	1	4
Thoma et al., 2017	Europe	Prospective and retrospective	NI	NP	1	4	29	Yes	Low	1	3
Thomas et al., 2020	Europe	Prospective and retrospective	Effective Public Health Practice Project (EPHPP)	CRD42019149543	1	0	8	Yes	Serious	4	1
Vetromila et al., 2020	America	RCT/NR	Cochrane RoB tool ROBINS-I	CRD42016048264	1	0	38	Yes	Low	1	1
Whang et al., 2022	Asia	RCT/NR	Newcastle-Ottawa Scale (NOS)	CRD42022277014	1	1	11	Yes	Low	1	0

NI: No information; NP: No protocol registration; SR: Systematic review; RCT: Randomized controlled trial; NR: Nonrandomized study

Overall, higher mean counts of spin were observed in the main text compared with the abstract across most review and journal characteristics (Table 2). The prevalence of spin in the abstract was 92.3%, with 24 studies exhibiting some level of spin in their abstracts^35-40,42-49,51,53-60^. The most common spin strategies in abstracts were failure to report conflicts of interest (34.61%) and failure to account for differences in study designs (34.61%). The prevalence of spin in the main text was 96.15%. The most common misleading reporting categories were “no or inadequate consideration of reporting bias in results interpretation (i.e., no reporting of an assessment of reporting bias, claims of efficacy despite evidence of reporting bias, claims of absence of reporting bias not supported by the data, interpretation of a negative test result as absence of publication bias, use of a test without meeting validity conditions, inadequate interpretation of a funnel plot)” (21 studies; 80.76%); “conclusion claiming equivalence or comparable effectiveness for nonsignificant results with wide confidence intervals” (26.31%); and “no or inadequate consideration of the risk of bias of primary studies in results interpretation (i.e., no reporting of the risk of bias of primary studies, claims of low risk of bias not supported by the data, no downgrading of evidence despite several high risk of bias studies)” (34.61%) (Table 2 and Figure 3).

**Figure 3:**
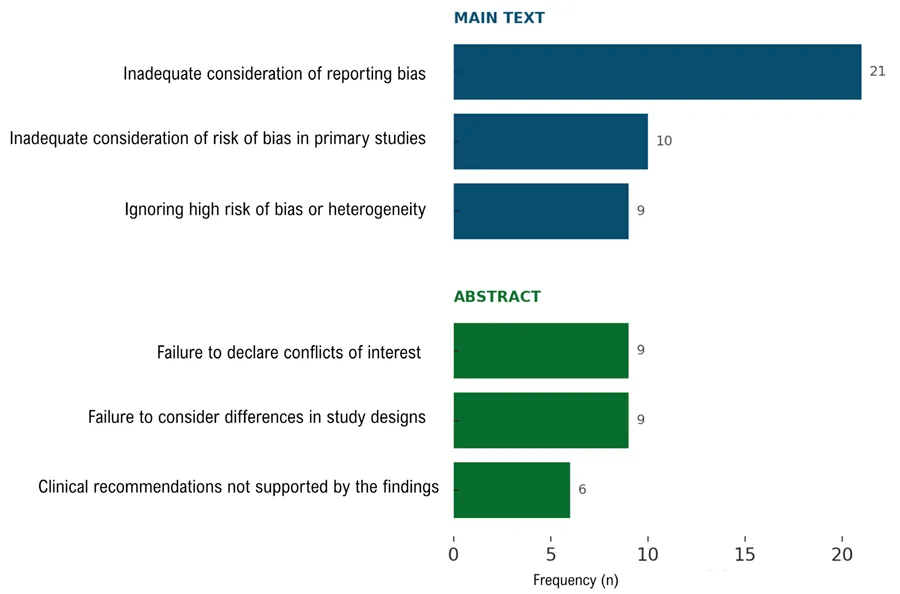
Most frequent spin strategies in abstracts and main texts.

### Reporting quality and risk of bias

The AMSTAR 2 tool was used to assess the methodological quality of SRs; most reviews were classified as critically low quality (73.08%), followed by low quality (19.23%). Only one review was rated as moderate quality (3.85%),^48^ and one as high quality (3.85%)^55^(Supplementary Material 6). Regarding risk of bias assessed using ROBIS, most reviews were classified as unclear (46.15%), followed by high risk of bias (38.46%), whereas four reviews were rated as low risk of bias (15.38%) (Supplementary Material 6 and Figure 3).

### Associated factors associated with spin occurrence

Ordinal logistic regression analysis evaluating potential factors associated with the level of spin in abstracts of SRs showed no statistically significant associations for any of the investigated variables (Supplementary Material 8). Regression analysis of spin occurrences in abstracts is presented in Table 4. After adjustment for confounding factors, manuscripts that did not comply with the recommended abstract word limit showed a 69% higher rate of spin occurrences (PR = 1.69, 95%CI: 1.19–2.42).

**Table 4:** Poisson regression analysis of potential predictors of spin occurrences in abstracts of systematic reviews (n = 26).

Varialbes	PR (Unadjusted)	p-value	PR (Ajusted)	p-value	Bonferroni-adjusted p value
Year					
≥ 2020	1	0.858			
< 2020	1.04 (0.61–1.78)				
Origin					
Asia	1	0.520			
America	1.12 (0.58–2.17)				
Europe	1.36 (0.78–2.39)				
Compliance with word limit					
Yes	1	0.695			
No	2.10 (0.66–1.82)				
Distinction of primary/secondary outcomes					
No	1	0.831			
Yes	1.04 (0.67–1.63)				
Journal endorsement of ICMJE					
Yes	1	0.941			
No	1.02 (0.50–2.06)				
Journal endorsement of PRISMA					
No	1	0.215	1	0.061	0.183
Yes	1.34 (0.84–2.15)		1.50 (0.98–2.31)		
Funding source					
No funding	1	0.028	1	0.004	0.016
Non-profit	1.45 (0.84–2.49)		1.19 (0.65–2.16)		
Not reported	2.17 (1.22–3.87)		2.10 (1.22–3.59)		
Manuscript citing PRISMA					
Yes	1	0.125			
No	1.50 (0.89–2.54)				
Manuscript open access					
Yes	1	0.202			
No	1.47 (0.81–2.65)				
Structured abstract					
No	1	0.001	1	0.069	0.207
Yes	1.71 (1.22–2.39)		1.45 (0.97–2.16)		
Conflict of interest reported					
Yes	1	0.095			
No	1.48 (0.93–2.38)				
Risk of bias assessment					
Yes	1	0.309			
No	1.35 (0.75–2.41)				
GRADE assessment					
Yes	1	0.661			
No	1.09 (0.72–1.64)				
Registered protocol					
Yes	1	0.966			
No	1.01 (0.60–1.68)				
ROBIS assessment					
Unclear	1	0.443			
High	1.40 (0.81–2.40)				
Low	1.10 (0.64–1.86)				

As shown in Table 4, most predictors were associated with the number of spin occurrences in abstracts. After multivariable adjustment, reviews with unreported funding had more spin occurrences compared with those declaring no funding (PR = 2.10, 95%CI: 1.22–3.59), and this association remained significant after Bonferroni correction (adjusted p = 0.016). Table 5 presents the Poisson regression analysis assessing potential predictors of spin occurrences in the main text of SRs. In adjusted models, absence of a structured abstract remained independently associated with a higher number of spin occurrences in the main text (PR = 1.56, 95%CI: 1.18–2.07), and this association persisted after Bonferroni correction (p = 0.008).

**Table 5:** Poisson regression analysis of potential predictors of spin occurrences in the main text of systematic reviews (n = 26).

Variables	PR (Unadjusted)	p-value	PR (Adjusted)	p-value	Bonferroni-adjusted p value
Year					
≥ 2020	1	<0.001	1	0.014	0.056
< 2020	1.80 (1.28–2.52)		1.81 (1.18–2.07)		
Continent					
Asia	1	0.781			
America	1.15 (0.64–2.07)				
Europe	1.18 (0.73–1.92)				
Compliance with word limit					
No	1	0.513			
Yes	1.17 (0.71–1.93)				
Distinction primary/secondary outcomes					
Yes	1	0.027			
No	1.69 (1.06–2.70)				
Journal endorsement of ICMJE					
Yes	1	0.250			
No	1.31 (0.82–2.08)				
Journal endorsement of PRISMA					
No	1	0.784			
Yes	1.06 (0.69–1.61)				
Funding source					
No funding	1	0.270			
Non-profit	1.11 (0.66–1.84)				
Not reported	1.42 (0.92–2.21)				
Manuscript citing PRISMA					
Yes	1	<0.001			
No	1.84 (1.37–2.47)				
Manuscript open access					
Yes	1	0.560			
No	1.17 (0.67–2.05)				
Structured abstract					
Yes	1	0.008	1	0.002	0.008
No	2.62 (1.86–3.69)		1.56 (1.18–2.07)		
Conflict of interest reported					
Yes	1	0.063			
No	2.19 (0.95–5.02)				
Risk of bias assessment					
Yes	1	0.005	1	0.087	
No	1.58 (1.14–2.19)		1.45 (0.94–2.21)		
GRADE assessment					
Yes	1	0.005			
No	2.61 (1.33–5.15)				
Protocol registration					
Yes	1	<0.001	1		
No	1.89 (1.30–2.74)		1.68 (1.08–2.61)	0.019	0.076
ROBIS assessment					
Low	1				
High	2.28 (0.75–6.95)	0.133			

In the sensitivity analysis (Supplementary Material 9), unreported funding and absence of a structured abstract remained significantly associated with higher spin counts after Bonferroni correction, whereas associations for publication year and protocol registration were attenuated. Supplementary Material 10 shows differences in the distribution of spin strategies between SRs of RCTs and NRs, with higher frequencies of unsupported clinical recommendations in NRs-based reviews and higher frequencies of selective emphasis and overinterpretation of efficacy outcomes in RCT-based reviews.

## Discussion

This study investigated the occurrence and types of spin in SRs with meta-analyses of NRs in restorative dentistry. The findings revealed a high occurrence of spin in both abstracts and main texts. Most studies did not fully meet reporting quality criteria and showed a considerable risk of bias, highlighting the need to improve the clarity of reported information. Additionally, factors potentially associated with spin included unreported funding and absence of a structured abstract.

These findings align with evidence showing that spin is highly prevalent in dental research. Recent meta-research reported spin in 30% to 86% of studies, including both randomized and nonrandomized designs.^62^In the present analysis, spin was identified in 92.3% of abstracts and 96.15% of main texts of SRs of NRs. Spin in abstracts is particularly concerning because many readers rely exclusively on this section, and biased framing may influence clinical decision-making, leading to suboptimal treatment choices.^27,63^In dentistry, spin often manifests as overinterpretation of nonsignificant findings or omission of study limitations, distorting the conclusions of SRs and meta-analyses.^22,64^This distortion may also affect clinical guidelines, reinforcing the need for rigorous reporting standards, critical peer review, and editorial oversight to reduce misleading interpretations in dental SRs.

**Table 3:** Distribution of spin according to review and journal characteristics: exploratory comparison of means (n = 26)

Variables	Total (%)	Number of spin items in abstract	Number of spin items in main text
		Mean (SD)	Mean (SD)
Year			
< 2020	9 (34.62)	2.22 (1.56)	4.55 (0.88)
≥ 2020	17 (65.38)	2.11 (1.31)	2.52 (1.69)
Origin			
Asia	9 (11.54)	1.77 (1.30)	2.88 (1.83)
America	3 (34.62)	2.00 (1.00)	3.33 (1.52)
Europe	14 (53.85)	2.72 (1.61)	3.42 (1.82)
Compliance with word limit			
No	19 (73.08)	2.21 (1.47)	3.36 (1.77)
Yes	7 (26.92)	2.00 (1.15)	2.85 (1.77)
Clear distinction of primary/secondary outcomes			
No	17 (65.38)	2.11 (1.57)	3.76 (1.67)
Yes	9 (34.62)	2.22 (0.97)	2.22 (1.48)
Journal endorsement of ICMJE			
No	5 (19.23)	2.20 (1.78)	4.00 (2.00)
Yes	21 (80.77)	2.14 (1.31)	3.04 (1.68)
Journal endorsement of PRISMA			
No	12 (46.15)	2.50 (1.62)	3.33 (2.05)
Yes	14 (53.85)	1.85 (1.09)	3.14 (1.51)
Funding source			
No funding	9 (34.62)	1.44 (1.01)	3.11 (2.02)
Non-profit	10 (38.46)	2.10 (1.10)	2.80 (1.39)
Not reported	7 (26.92)	3.14 (1.67)	4.00 (1.82)
Manuscript citing PRISMA			
No	7 (26.92)	2.85 (1.86)	4.85 (0.69)
Yes	19 (73.08)	1.89 (1.10)	2.63 (1.64)
Manuscript open access			
No	18 (69.23)	2.38 (1.37)	3.38 (1.53)
Yes	8 (30.77)	1.62 (1.30)	2.87 (2.23)
Structured abstract			
No	2 (7.69)	3.50 (0.70)	5.00 (1.14)
Yes	24 (92.31)	2.04 (1.36)	3.08 (1.71)
Conflict of interest reporting			
No	10 (38.46)	2.70 (1.63)	3.70 (1.41)
Yes	16 (61.54)	1.81 (1.10)	2.93 (1.91)
Risk of bias assessment			
No	7 (26.92)	1.71 (1.25)	4.42 (0.97)
Yes	19 (73.08)	2.31 (1.41)	2.78 (1.78)
GRADE assessment			
No	21 (80.77)	2.19 (1.50)	3.66 (1.59)
Yes	5 (19.23)	2.00 (0.70)	1.40 (1.14)
Protocol registration			
No	12 (46.15)	2.16 (1.79)	4.33 (1.43)
Yes	14 (53.85)	2.14 (1.02)	2.28 (1.43)
ROBIS assessment			
Low	4 (15.38)	2.00 (0.81)	1.75 (2.21)
Unclear	11 (42.31)	2.28 (0.95)	3.00 (1.67)
High	11 (42.31)	1.81 (1.25)	4.00 (1.34)

**Figure 4:**
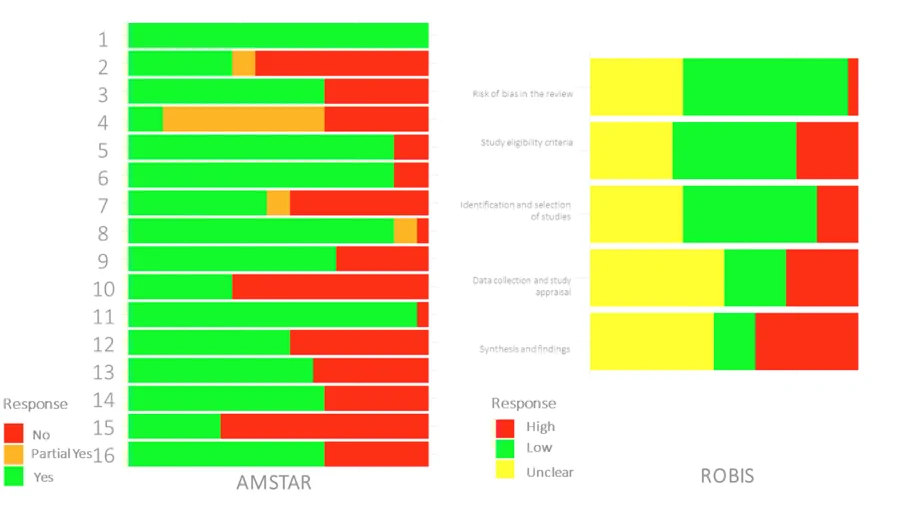
AMSTAR 2 and ROBIS tools.

In the main text, the most frequent spin strategy was no or inadequate consideration of reporting bias in results interpretation, observed in 21 studies (80.8%). This pattern suggests that, even when quantitative syntheses are performed, many authors fail to critically appraise whether selective publication or selective reporting may have distorted the available evidence. As emphasized in the current guidance for SRs, assessment of reporting bias is a key component in judging the credibility and certainty of effect estimates, and should be explicitly incorporated into the interpretation of findings and strength-of-evidence ratings^65,66^. When reviewers omit this assessment, interpret nonsignificant tests and imperfect funnel plots as evidence of “no publication bias,” or do not perform this analysis, the risk lies in overstating the apparent benefits or safety of interventions and providing overly optimistic messages for clinical practice.^67,68^

Reporting guidelines have a crucial role in shaping clinical practice and ensuring high-quality care.^69^ Clinical practice and public health guidelines have evolved in response to variation in health decision-making at both individual and population levels. These guidelines should contain systematically developed, evidence-informed, actionable statements (recommendations) that assist patients, clinicians, policymakers, and other stakeholders in making healthcare and public health decisions.^70,71^ The overarching goal of guidelines is not only to improve clinical care and minimize unjustified variation in practice but also to avoid unnecessary or ineffective services and ensure effective allocation of healthcare resources.^72^

One of the main predictors associated with a higher occurrence of spin in abstracts was failure to report funding sources. This finding does not necessarily indicate that SRs received external funding; however, according to the PRISMA checklist, authors should explicitly report sources of financial or nonfinancial support, the role of funders, or formally declare absence of funding. In the present study, although 19 reviews reported adherence to PRISMA, these same reviews did not report funding information, highlighting incomplete compliance with the guideline^30^. The absence of funding disclosure may reflect broader deficits in methodological rigor and transparency, which may be associated with a higher probability of spin. Transparency regarding funding and potential conflicts of interest is central to the credibility of scientific evidence, particularly in SRs. In this context, journals have increasingly implemented stricter policies requiring disclosure of financial and nonfinancial conflicts of interest, as well as explicit statements when none exist.^73,74^

Journal requirements for abstract structure vary substantially, which may influence how results are reported and interpreted.^75^ In this study, absence of a structured abstract in SRs and meta-analyses was associated with a higher prevalence of spin. Consistent with previous research, adherence to structured reporting guidelines, such as PRISMA, has been associated with greater transparency.^76^ Structured abstracts prompt authors to report key methodological elements, limitations, and sources of uncertainty—information that may be omitted in narrative formats.^77,78^ Because abstract structure is largely determined by journal policies and editorial practices, journals that allow unstructured abstracts may inadvertently facilitate selective reporting. Strengthening requirements for structured abstracts may therefore represent a practical strategy to reduce spin and improve the rigor of SR reporting.^79^

The inclusion of SRs published over a broad time period (1998–2025) allowed us to explore temporal changes in methodological and reporting practices. For the multivariable analysis, publication year was dichotomized using 2020 as the cutoff, reflecting the release of the PRISMA 2020 Statement, which introduced updated and more stringent recommendations for transparency and reporting of SRs.^80,81^ Although guidelines such as PRISMA, GRADE, and protocol registration have gained greater prominence in recent years, their limited uptake in earlier publications may have increased susceptibility to spin due to weaker methodological and reporting safeguards. Despite the absence of a statistically significant association between publication year and spin prevalence, the deficiencies observed in older reviews remain a relevant concern. Together, these findings underscore the importance of adherence to contemporary reporting standards and structured protocols, which enhance transparency, mitigate biased interpretation of results, and ultimately strengthen the credibility of evidence syntheses.

The quality assessment of SRs revealed discrepancies between AMSTAR 2 and ROBIS, underscoring the importance of cautious interpretation. AMSTAR 2 rated 73.6% of studies as critically low quality, largely reflecting its strict criteria that heavily penalize incomplete reporting.^82^ ROBIS, on the other hand, classified 42.1% of studies as high risk of bias, adopting a domain-based approach with signaling questions that facilitate the identification of potential methodological concerns.^29^ These differences are particularly relevant in the context of spin because studies with poor reporting or methodological limitations are more likely to yield misleading conclusions, underscoring the importance of robust reporting and adherence to established guidelines in SRs of NRs.

The comparative analysis between RCTs and NRs included in the SR reveals important differences in the distribution of spin strategies. Reviews of NRs showed consistently higher proportions of unsupported clinical recommendations in both abstracts and full texts, suggesting a greater tendency to translate uncertain or heterogeneous evidence into practice-oriented statements^2^. This finding is particularly concerning, since nonrandomized studies are inherently more susceptible to confounding bias, which should warrant greater caution in the formulation of clinical recommendations. SRs of NRs more frequently presented unsupported clinical recommendations in both abstracts and full texts, indicating a greater tendency to extend conclusions beyond the underlying evidence. Conversely, reviews of randomized trials more often showed selective emphasis and overenthusiastic interpretation of efficacy outcomes, particularly in abstracts. These findings suggest that spin is not limited to lower levels of evidence but rather assumes different forms depending on study design.

The findings suggest that researchers should improve reporting practices, including research ethics and integrity and the implementation of open science practices. Understanding the relationship between open science and research ethics and integrity is critical to prevent conflicting messages regarding ethical responsibilities and to ensure that research evaluation systems support, rather than undermine, ethical research practices.^83^ In this context, journal editors and peer reviewers play an important role in promoting transparent and responsible research practices. Editorial policies should enforce adherence to protocol registration, conflict of interest disclosures, and structured reporting guidelines. These measures are essential to reduce spin and improve the reliability of evidence synthesis, ultimately strengthening the foundation for evidence-based clinical decision-making.

Some limitations of this study should be acknowledged. Identification of spin is inherently subjective; however, this limitation was mitigated through predefined criteria, independent duplicate assessments, and verification by an experienced reviewer trained in clinical epidemiology. Although the classification tool provides a robust framework for detecting spin, it may be overly stringent and less suitable for certain outcomes, particularly in studies with ambiguous reporting. Nonetheless, its systematic application enhanced consistency and reduced the risk of misclassification. In addition, the relatively small number of included SRs may have limited the statistical power of multivariable analyses, resulting in wide CIs and potentially obscuring subtle associations. As more SRs of NRs are published, future research may include larger samples, allowing more precise estimates and a clearer identification of factors associated with spin.

## Conclusions

This study revealed a high prevalence of spin in SRs of NRs in restorative dentistry, affecting 92.30% of abstracts and 96.15% of main texts. Factors such as absence of structured abstracts and unreported funding were associated with spin, reinforcing the need for stricter editorial policies and reporting standards. Most reviews were classified as critically low quality and had a high or unclear risk of bias, underscoring the need for improved reporting and methodological rigor. These findings highlight the need for cautious interpretation of SRs of NRs, given their potential influence on clinical decision-making.

## Supplementary materials

Supplementary materials are publicly available in the Open Science Framework

Supplementary Material 1

Supplementary Material 2

Supplementary Material 3

Supplementary Material 4

Supplementary Material 5

Supplementary Material 6

Supplementary Material 7

Supplementary Material 8

Supplementary Material 9

Supplementary Material 10

## Data Availability

The datasets generated during and/or analyzed during the current study are available from the corresponding author on reasonable request.

## References

[1] Boutron I, Ravaud P (2018). Misrepresentation and distortion of research in biomedical
literature. Proceedings of the National Academy of Sciences.

[2] Sensever FA, CP de Lucena Alves, Lima G da S, Loomans B, Opdam N, Pereira-Cenci T (2022). Spin and reporting in systematic reviews with meta-analysis of
randomized clinical trials in restorative dentistry. J Dent.

[3] Boutron I (2010). Reporting and Interpretation of Randomized Controlled Trials With
Statistically Nonsignificant Results for Primary Outcomes. JAMA.

[4] Munafò MR, Nosek BA, Bishop DVM, Button KS, Chambers CD, Percie Du Sert N (2017). A manifesto for reproducible science. Nat Hum Behav.

[5] Pereira-Cenci T, Cademartori MG, dos Santos LG, Corrêa MB, Loomans B, Horta BL (2023). Prevalence of tooth wear and associated factors: A birth cohort
study. J Dent.

[6] Demarco FF, Corrêa MB, Cenci MS, Moraes RR, Opdam NJM (2012). Longevity of posterior composite restorations: Not only a matter
of materials. Dent Mater.

[7] Frankenberger R, Reinelt C, Petschelt A, Krämer N (2009). Operator vs. material influence on clinical outcome of bonded
ceramic inlays. Dent Mater.

[8] Concato J, Shah N, Horwitz RI (2000). Randomized, Controlled Trials, Observational Studies, and the
Hierarchy of Research Designs. N Engl J Med.

[9] Tarazona-Álvarez B, López-Roldán A, Vidal-Infer A, Alonso-Arroyo A (2023). Bibliometric study of the systematic reviews and meta-analyses in
dentistry. J Clin Exp Dent.

[10] Patole S (2021). Systematic Reviews and Meta-Analyses of Non-randomised Studies.
Principles and Practice of Systematic Reviews and Meta-Analysis.

[11] Prott LS, Carrasco-Labra A, Gierthmuehlen PC, Blatz MB (2025). How to Conduct and Publish Systematic Reviews and Meta-Analyses
in Dentistry. J Esthet Restor Dent.

[12] Bärnighausen T, Røttingen J-A, Rockers P, Shemilt I, Tugwell P (2017). Quasi-experimental study designs series-paper 1: introduction:
two historical lineages. J Clin Epidemiol.

[13] Quigley JM, Thompson JC, Halfpenny NJ, Scott DA (2019). Critical appraisal of nonrandomized studies-A review of
recommended and commonly used tools. J Eval Clin Pract.

[14] Cuello-Garcia CA, Morgan RL, Brozek J, Santesso N, Verbeek J, Thayer K (2018). A scoping review and survey provides the rationale, perceptions,
and preferences for the integration of randomized and nonrandomized studies
in evidence syntheses and GRADE assessments. J Clin Epidemiol.

[15] Sarri G, Patorno E, Yuan H, Guo J, Bennett D, Wen X (2022). Framework for the synthesis of non-randomised studies and
randomised controlled trials: a guidance on conducting a systematic review
and meta-analysis for healthcare decision making. BMJ Evid Based Med.

[16] Ioannidis JPA (2007). Limitations are not properly acknowledged in the scientific
literature. J Clin Epidemiol.

[17] Page MJ, Shamseer L, Altman DG, Tetzlaff J, Sampson M, Tricco AC (2016). Epidemiology and Reporting Characteristics of Systematic Reviews
of Biomedical Research: A Cross-Sectional Study. PLoS Med.

[18] Yavchitz A, Boutron I, Bafeta A, Marroun I, Charles P, Mantz J (2012). Misrepresentation of Randomized Controlled Trials in Press
Releases and News Coverage: A Cohort Study. PLoS Med.

[19] Gates M, Gates A, Pieper D, Fernandes RM, Tricco AC, Moher D (2022). Reporting guideline for overviews of reviews of healthcare
interventions: development of the PRIOR statement. BMJ.

[20] Murad MH, Wang Z (2017). Guidelines for reporting meta-epidemiological methodology
research. Evid Based Med.

[21] Axon E, Kanellopoulou A, Tsokani S, Livingstone N, Hilgart J, Richardson R (2025). Incorporating non-randomized evidence in Cochrane intervention
reviews: a scoping review. J Clin Epidemiol.

[22] Roszhart JI, Kumar SS, Allareddy V, Childs CA, Elangovan S (2020). Spin in abstracts of randomized controlled trials in
dentistry. J Am Dent Assoc.

[23] Gallo L, Yuan M, Gallo M, Chin B, Huynh MNQ, McRae M (2022). Evaluation of "Spin" in the Abstracts of Systematic Reviews and
Meta-Analyses of Therapeutic Interventions Published in High-Impact Plastic
Surgery Journals: A Systematic Review. Aesthet Surg J.

[24] Ghannad M, Olsen M, Boutron I, Bossuyt PM (2019). A systematic review finds that spin or interpretation bias is
abundant in evaluations of ovarian cancer biomarkers. J Clin Epidemiol.

[25] Nowlin R, Wirtz A, Wenger D, Ottwell R, Cook C, Arthur W (2022). Spin in Abstracts of Systematic Reviews and Meta-analyses of
Melanoma Therapies: Cross-sectional Analysis. JMIR Dermatol.

[26] Guyatt GH, Oxman AD, Sultan S, Glasziou P, Akl EA, Alonso-Coello P (2011). GRADE guidelines: 9. Rating up the quality of
evidence. J Clin Epidemiol.

[27] Yavchitz A, Ravaud P, Altman DG, Moher D, Hrobjartsson A, Lasserson T (2016). A new classification of spin in systematic reviews and
meta-analyses was developed and ranked according to the
severity. J Clin Epidemiol.

[28] Lu C, Lu T, Ge L, Yang N, Yan P, Yang K (2020). Use of AMSTAR-2 in the methodological assessment of systematic
reviews: protocol for a methodological study. Ann Transl Med.

[29] Whiting P, Savovic J, Higgins JPT, Caldwell DM, Reeves BC, Shea B (2016). ROBIS: A new tool to assess risk of bias in systematic reviews
was developed. J Clin Epidemiol.

[30] Page MJ, McKenzie JE, Bossuyt PM, Boutron I, Hoffmann TC, Mulrow CD (2021). The PRISMA 2020 statement: an updated guideline for reporting
systematic reviews. BMJ.

[31] Bendel RB, Afifi AA (1977). Comparison of Stopping Rules in Forward "Stepwise"
Regression. J Am Stat Assoc.

[32] Hosmer DW., Lemeshow Stanley., Sturdivant RX. (2013). Applied logistic regression.

[33] Bender R, Lange S (2001). Adjusting for multiple testing-when and how?. J Clin Epidemiol.

[34] Rothman KJ (1990). No adjustments are needed for multiple
comparisons. Epidemiology.

[35] Albanesi RB, Pigozzo MN, Sesma N, Laganá DC, Morimoto S (2016). Incisal coverage or not in ceramic laminate veneers: A systematic
review and meta-analysis. J Dent.

[36] Alenezi AA, Alyahya SO, Aldakhail NS, Alsalhi HA (2024). Clinical behavior and survival of endodontically treated teeth
with or without post placement: a systematic review and
meta-analysis. J Oral Sci.

[37] Alqutaibi AY, Alghauli MA, Almuzaini SA, Alharbi AF, Alsani AA, Mubarak AM (2024). Failure and complication rates of different materials, designs,
and bonding techniques of ceramic cantilever resin-bonded fixed dental
prostheses for restoring missing anterior teeth: A systematic review and
meta-analysis. J Esthet Restor Dent.

[38] Chantler JGM, Pirc M, Strauss FJ, Rohr N, Thoma DS, Ioannidis A (2025). Rehabilitation of the Worn Dentition With Direct and Indirect
Minimally Invasive Concepts-A Systematic Review and
Meta-Analysis. J Esthet Restor Dent.

[39] Bresser RA, Hofsteenge JW, Wieringa TH, Braun PG, Cune MS, Özcan M (2023). Clinical longevity of intracoronal restorations made of gold,
lithium disilicate, leucite, and indirect resin composite: a systematic
review and meta-analysis. Clin Oral Investig.

[40] Dioguardi M, Alovisi M, Comba A, Baldi A, Troiano G, Cadenaro M (2022). The influence of indirect bonded restorations on clinical
prognosis of endodontically treated teeth: A systematic review and
meta-analysis. Dent Mater.

[41] Dioguardi M, Alovisi M, Troiano G, Caponio CVA, Baldi A, Rocca GT (2021). Clinical outcome of bonded partial indirect posterior
restorations on vital and non-vital teeth: a systematic review and
meta-analysis. Clin Oral Investig.

[42] Wang B, Fan J, Wang L, Xu B, Wang L, Chai L (2022). Onlays/partial crowns versus full crowns in restoring posterior
teeth: a systematic review and meta-analysis. Head Face Med.

[43] Fan J, Wang B, Wang L, Xu B, Wang L, Wang C (2025). Clinical performance of minimally invasive full-mouth
rehabilitation using different materials and techniques for patients with
moderate to severe tooth wear: a systematic review and
meta-analysis. Clin Oral Investig.

[44] Heintze SD, Cavalleri A, Forjanic M, Zellweger G, Rousson V (2008). Wear of ceramic and antagonist-A systematic evaluation of
influencing factors in vitro. Dent Mater.

[45] Fathi AH, Dezaki SN, Khodadadi R (2022). Evaluation of the survival and complication rates of monolithic
single crowns and fixed partial dentures: a systematic review and
meta-analysis. Int J Med Dent.

[46] Kassardjian V, Varma S, Andiappan M, Creugers NHJ, Bartlett D (2016). A systematic review and meta-analysis of the longevity of
anterior and posterior all-ceramic crowns. J Dent.

[47] Kreulen CM, Creugers NHJ, Meijering AC (1998). Meta-analysis of anterior veneer restorations in clinical
studies. J Dent.

[48] Pjetursson BE, Sailer I, Zwahlen M, Hämmerle CHF (2007). A systematic review of the survival and complication rates of
all-ceramic and metal-ceramic reconstructions after an observation period of
at least 3 years. Part I: Single crowns. Clin Oral Implants Res.

[49] Ploumaki A, Bilkhair A, Tuna T, Stampf S, Strub JR (2013). Success rates of prosthetic restorations on endodontically
treated teeth; a systematic review after 6 years. J Oral Rehabil.

[50] Thomas RM, Kelly A, Tagiyeva N, Kanagasingam S (2020). Comparing endocrown restorations on permanent molars and
premolars: a systematic review and meta-analysis. Br Dent J.

[51] Al-Dabbagh RA (2021). Survival and success of endocrowns: A systematic review and
meta-analysis. J Prosthet Dent.

[52] Thoma DS, Sailer I, Ioannidis A, Zwahlen M, Makarov N, Pjetursson BE (2017). A systematic review of the survival and complication rates of
resin-bonded fixed dental prostheses after a mean observation period of at
least 5 years. Clin Oral Implants Res.

[53] Vetromilla BM, Opdam NJ, Leida FL, Sarkis-Onofre R, Demarco FF, van der Loo MPJ (2020). Treatment options for large posterior restorations: a systematic
review and network meta-analysis. J Am Dent Assoc.

[54] Bhagwat S, Mandke L, Vandekar M, Basmatkar N, Pawar A, Khatri R (2025). Longevity of Amalgam Versus Composite Resin Restorations in
Permanent Posterior Teeth: A Systematic Review. Cureus.

[55] Dewan H (2023). Clinical Effectiveness of 3D-Milled and 3D-Printed Zirconia
Prosthesis-A Systematic Review and Meta-Analysis. Biomimetics (Basel).

[56] Lim TW, Tan SK, Li KY, Burrow MF (2023). Survival and complication rates of resin composite laminate
veneers: a systematic review and meta-analysis. J Evid Based Dent Pract.

[57] Mendes LT, Pedrotti D, Casagrande L, Lenzi TL (2022). Risk of failure of repaired versus replaced defective direct
restorations in permanent teeth: a systematic review and
meta-analysis. Clin Oral Investig.

[58] Sailer I, Pjetursson BE, Zwahlen M, Hämmerle CHF (2007). A systematic review of the survival and complication rates of
all-ceramic and metal-ceramic reconstructions after an observation period of
at least 3 years. Part II: fixed dental prostheses. Clin Oral Implants Res.

[59] Sailer I, Strasding M, Valente NA, Zwahlen M, Liu S, Pjetursson BE (2018). A systematic review of the survival and complication rates of
zirconia-ceramic and metal-ceramic multiple-unit fixed dental
prostheses. Clin Oral Implants Res.

[60] Pjetursson BE, Tan K, Lang NP, Brägger U, Egger M, Zwahlen M (2004). A systematic review of the survival and complication rates of
fixed partial dentures (FPDs) after an observation period of at least 5
years. Clin Oral Implants Res.

[61] Dioguardi M, Alovisi M, Troiano G, Caponio CVA, Baldi A, Rocca GT (2021). Clinical outcome of bonded partial indirect posterior
restorations on vital and non-vital teeth: a systematic review and
meta-analysis. Clin Oral Investig.

[62] Cruz LR, Braga SF, Nadanovsky P, Santos APP dos (2024). Spin in dental publications: a scoping review. Braz Oral Res.

[63] Chiu K, Grundy Q, Bero L (2017). 'Spin' in published biomedical literature: A methodological
systematic review. PLoS Biol.

[64] Eleftheriadi I, Ioannou T, Pandis N (2020). Extent and prevalence of spin in randomized controlled trials in
dentistry. J Dent.

[65] Higgins JPT, Green S Cochrane Handbook for Systematic Reviews of Interventions.

[66] Kolaski K, Logan LR, Ioannidis JPA (2023). Guidance to best tools and practices for systematic
reviews. BMC Infect Dis.

[67] Page MJ, McKenzie JE, Higgins JPT (2018). Tools for assessing risk of reporting biases in studies and
syntheses of studies: a systematic review. BMJ Open.

[68] Koletsi D, Valla K, Fleming PS, Chaimani A, Pandis N (2016). Assessment of publication bias required improvement in oral
health systematic reviews. J Clin Epidemiol.

[69] Schünemann HJ, Reinap M, Piggott T, Laidmäe E, Köhler K, Pold M (2022). The ecosystem of health decision making: from fragmentation to
synergy. Lancet Public Health.

[70] Falciola L, Barbieri M (2023). Disclosure of Patenting Activities within Scientific Publications
as Potential Conflict-of-Interests: Evidences from Biomedical
Literature. Research Square.

[71] World Health Organization (2015). Who Handbook for Guideline Development.

[72] Verdugo-Paiva F, Rojas-Gómez AM, Wielandt V, Peña J, Silva-Ruz I, Novillo F (2024). Evidence-informed guidelines in oral health: insights from a
systematic survey. BMC Oral Health.

[73] Vasconcelos SMR, Cassimiro MC, Martins MFM, Palácios M (2013). Addressing conflicts of interest in the research paper: a
societal demand in contemporary science?. Braz J Med Biol Res.

[74] Goldberg DS (2020). Financial Conflicts of Interest are of a higher ethical priority
than "Intellectual" Conflicts of Interest. J Bioeth Inq.

[75] Rennie D, Glass RM (1991). Structuring Abstracts to Make Them More
Informative. JAMA.

[76] Carter-Templeton H, Oermann MH, Owens JK, Vance B, Mastorovich ML, Quazi M (2025). Completeness of Systematic Reviews in Nursing Literature Based on
PRISMA Reporting Guidelines. ANS Adv Nurs Sci.

[77] Nakayama T, Hirai N, Yamazaki S, Naito M (2005). Adoption of structured abstracts by general medical journals and
format for a structured abstract. J Med Libr Assoc.

[78] Sharma S, Harrison JE (2006). Structured abstracts: Do they improve the quality of information
in abstracts?. Am J Orthod Dentofacial Orthop.

[79] Pierson CA (2016). Structured abstracts improve clarity and reach your intended
audience. J Am Assoc Nurse Pract.

[80] Patro P, Misra DP (2021). Registering and reporting systematic reviews. Cent Asian J Med Hypotheses Ethics.

[81] Carlo Torres G, Ledbetter L, Cantrell S, Alomo ARL, Blodgett TJ, Bongar MV (2024). Adherence to PRISMA 2020 reporting guidelines and scope of
systematic reviews published in nursing: A cross-sectional
analysis. J Nurs Scholarsh.

[82] Shea BJ, Reeves BC, Wells G, Thuku M, Hamel C, Moran J (2017). AMSTAR 2: a critical appraisal tool for systematic reviews that
include randomised or non-randomised studies of healthcare interventions, or
both. BMJ.

[83] Lindemann T, Häberlein L (2023). Contours of a research ethics and integrity perspective on open
science. Front Res Metr Anal.

