# Supplementary material for: Spin and reporting quality in systematic reviews of nonrandomized studies in restorative dentistry: a meta-research study
Source: Braz Oral Res. 2026 Nov 9;40:e094. doi: 10.1590/1807-3107bor-2026.vol40.094 (PMC13750809; doi:10.1590/1807-3107bor-2026.vol40.094)
Supplement: Supplementary file 2 [file 1807-3107-bor-40-e094-suppl02.pdf]

Supplementary Table 2. Electronic databases and search strategy.

Electronic Database	Search Strategy
PubMed MEDLINE 4715	**#1 Restorative Dentistry terms:**<br>Dental Restoration, Permanent[Mesh] OR Dental Restorations, Permanent[tiab] OR Permanent Dental Restoration OR Permanent Dental Restorations OR Restoration, Permanent Dental[tiab] OR Restorations, Permanent Dental[tiab] OR Dental Permanent Fillings[tiab] OR Fillings, Dental Permanent[tiab] OR Fillings, Permanent Dental[tiab] OR Permanent Dental Fillings OR Permanent Fillings, Dental[tiab] OR Dental Fillings, Permanent[tiab] OR Dental Filling, Permanent[tiab] OR Dental Permanent Filling[tiab] OR Filling, Dental Permanent[tiab] OR Filling, Permanent Dental[tiab] OR Permanent Dental Filling OR Permanent Filling, Dental[tiab] OR Dental Restoration, Temporary[Mesh] OR Restoration, Temporary Dental OR Restorations, Temporary Dental[tiab] OR Temporary Dental Restoration OR Temporary Dental Restorations OR Dental Fillings, Temporary[tiab] OR Dental Filling, Temporary OR Filling, Temporary Dental[tiab] OR Temporary Dental Filling OR Fillings, Temporary Dental[tiab] OR Temporary Dental Fillings OR Dental Prosthesis, Temporary[tiab] OR Dental Prostheses, Temporary[tiab] OR Prosthesis, Temporary Dental[tiab] OR Temporary Dental Prostheses OR Temporary Dental Prosthesis OR Dentistry, Operative OR Operative Dentistry OR dental restoration OR restorative dentistry OR Dental Restoration Repair OR Dental Restoration Repairs OR Repair, Dental Restoration OR Repairs, Dental Restoration[tiab] OR Restoration Repair, Dental[tiab] OR Restoration Repairs, Dental[tiab] OR Dental Restoration Failure OR Failure, Dental Restoration[tiab] OR Restoration Failure, Dental[tiab] OR Restoration Failures, Dental[tiab] OR Dental Restoration Failures OR Failures, Dental Restoration[tiab] OR Crowns OR Crown OR Dental Crowns OR Dental Crown OR Crown, Dental OR Crowns, Dental OR tooth crown OR Dental Veneers OR Veneer, Dental OR Veneers, Dental[tiab] OR Dental Laminates OR Dental Laminate OR Laminate, Dental[tiab] OR Laminates, Dental[tiab] OR Dental Veneer OR tooth veneer OR Dental Restoration Wear OR Restoration Wear, Dental[tiab] OR Wear, Dental Restoration[tiab] OR Restoration Occlusal Wear OR Wear, Restoration Occlusal[tiab] OR Occlusal Wear, Restoration OR Denture Wear OR Wear, Denture OR Dental Prosthesis OR Dental Prostheses OR Prostheses, Dental OR Prosthesis, Dental OR Post and Core Technique OR Post and Core Technic OR Post-Core Technic OR Post-Core Technics OR Technic, Post-Core[tiab] OR Technics, Post-Core[tiab] OR Post Technique OR Post Techniques OR Technique, Post OR Techniques, Post OR Post Technic OR Post Technics[tiab] OR Technic, Post[tiab] OR Technics, Post[tiab] OR Dental Dowel OR Dowel, Dental[tiab] OR Dowels, Dental[tiab] OR Dental Dowels OR Dental Prosthesis Repair[Mesh] OR Dental Prosthesis Repairs[tiab] OR Prosthesis Repair, Dental[tiab] OR Prosthesis Repairs, Dental[tiab] OR Repair, Dental Prosthesis[tiab] OR Repairs, Dental Prosthesis[tiab] OR Dental Prosthesis Retention[Mesh] OR Dental Prosthesis Retention OR Denture Retention<br><br>**#2 Systematic Review terms:**<br>Systematic Review[Publication Type] OR Systematic Reviews as Topic[Mesh] OR systematic review[ti]<br><br>**#3 Meta-analysis terms:**

	Meta-Analysis OR Meta-Analysis as Topic OR Meta Analysis as Topic OR Data Pooling OR Data Poolings OR Clinical Trial Overviews OR Clinical Trial Overview OR Overview, Clinical Trial OR Network Meta-Analysis OR Meta-Analyses, Network OR Meta-Analysis, Network OR Network Meta Analysis OR Network Meta-Analyses OR Multiple Treatment Comparison Meta-Analysis OR Multiple Treatment Comparison Meta Analysis OR Mixed Treatment Meta-Analysis OR Meta-Analysis, Mixed Treatment OR Mixed Treatment Meta Analysis OR Mixed Treatment Meta-Analyses OR meta analysis OR analysis, meta OR metaanalysis OR metaanalyses<br>#1 AND #2 AND #3
Embase 2385	**#1:** ('permanent dental restoration'/exp OR 'dental restoration'/exp OR 'operative dentistry'/exp OR 'restorative dentistry'/exp OR 'dental crown'/exp OR 'crown'/exp OR 'dental veneer'/exp OR 'post and core technique'/exp OR 'dowel pin'/exp OR 'dental restoration repair'/exp OR 'dental restoration failure'/exp OR 'tooth wear'/exp OR 'prosthesis repair'/exp OR 'denture retention'/exp OR 'dental prosthesis'/exp OR 'permanent dental restoration':ti,ab OR 'permanent dental restorations':ti,ab OR 'restoration, permanent dental':ti,ab OR 'restorations, permanent dental':ti,ab OR 'permanent dental filling':ti,ab OR 'permanent dental fillings':ti,ab OR 'dental permanent filling':ti,ab OR 'dental permanent fillings':ti,ab OR 'filling, dental permanent':ti,ab OR 'fillings, dental permanent':ti,ab OR 'temporary dental restoration':ti,ab OR 'temporary dental restorations':ti,ab OR 'restoration, temporary dental':ti,ab OR 'restorations, temporary dental':ti,ab OR 'temporary dental filling':ti,ab OR 'temporary dental fillings':ti,ab OR 'filling, temporary dental':ti,ab OR 'fillings, temporary dental':ti,ab OR 'restorative dentistry':ti,ab OR 'dental restoration':ti,ab OR 'operative dentistry':ti,ab OR 'crown':ti,ab OR 'crowns':ti,ab OR 'dental crown':ti,ab OR 'dental crowns':ti,ab OR 'tooth crown':ti,ab OR 'dental veneer':ti,ab OR 'dental veneers':ti,ab OR 'veneer, dental':ti,ab OR 'veneers, dental':ti,ab OR 'dental laminate':ti,ab OR 'dental laminates':ti,ab OR 'tooth veneer':ti,ab OR 'post and core technique':ti,ab OR 'post and core technic':ti,ab OR 'post-core technic':ti,ab OR 'post technique':ti,ab OR 'post techniques':ti,ab OR 'dental dowel':ti,ab OR 'dental dowels':ti,ab OR 'dental restoration repair':ti,ab OR 'dental restoration repairs':ti,ab OR 'repair, dental restoration':ti,ab OR 'restoration repair, dental':ti,ab OR 'dental restoration failure':ti,ab OR 'dental restoration failures':ti,ab OR 'failure, dental restoration':ti,ab OR 'restoration failure, dental':ti,ab OR 'dental restoration wear':ti,ab OR 'restoration wear, dental':ti,ab OR 'wear, dental restoration':ti,ab OR 'restoration occlusal wear':ti,ab OR 'denture wear':ti,ab OR 'dental prosthesis repair':ti,ab OR 'dental prosthesis repairs':ti,ab OR 'prosthesis repair, dental':ti,ab OR 'repair, dental prosthesis':ti,ab OR 'dental prosthesis retention':ti,ab OR 'denture retention':ti,ab OR 'temporary dental prosthesis':ti,ab OR 'temporary dental prostheses':ti,ab OR 'dental prosthesis, temporary':ti,ab OR 'dental prostheses, temporary':ti,ab OR 'prosthesis, temporary dental':ti,ab OR 'dental prosthesis':ti,ab OR 'dental prostheses':ti,ab OR 'prostheses, dental':ti,ab OR 'prosthesis, dental':ti,ab)<br><br>**#2:** 'systematic review'/exp OR 'systematic review (topic)'/exp OR 'systematic review':ti<br><br>**#3:** 'meta analysis'/exp OR 'meta analysis (topic)'/exp OR 'network meta-analysis'/exp OR 'data pooling'/exp OR 'clinical trial'/exp OR 'overview'/exp OR 'meta analysis':ti,ab OR 'meta-analysis':ti,ab OR 'metaanalysis':ti,ab OR 'metaanalyses':ti,ab OR 'data pooling':ti,ab OR 'data poolings':ti,ab OR 'clinical trial overview':ti,ab OR 'clinical trial overviews':ti,ab OR 'overview, clinical trial':ti,ab OR 'network meta-analysis':ti,ab OR 'network meta

	analyses':ti,ab OR 'network meta analysis':ti,ab OR 'multiple treatment comparison meta-analysis':ti,ab OR 'multiple treatment comparison meta analysis':ti,ab OR 'mixed treatment meta-analysis':ti,ab OR 'mixed treatment meta analysis':ti,ab OR 'mixed treatment meta-analyses':ti,ab<br><br>#1 AND #2 AND #3
Scopus<br>1496	#1 TITLE-ABS-KEY("Dental Restoration, Permanent" OR "Dental Restorations, Permanent" OR "Permanent Dental Restoration" OR "Permanent Dental Restorations" OR "Restoration, Permanent Dental" OR "Restorations, Permanent Dental" OR "Dental Permanent Fillings" OR "Fillings, Dental Permanent" OR "Fillings, Permanent Dental" OR "Permanent Dental Fillings" OR "Permanent Fillings, Dental" OR "Dental Fillings, Permanent" OR "Dental Filling, Permanent" OR "Dental Permanent Filling" OR "Filling, Dental Permanent" OR "Filling, Permanent Dental" OR "Permanent Dental Filling" OR "Permanent Filling, Dental" OR "Dental Restoration, Temporary" OR "Restoration, Temporary Dental" OR "Restorations, Temporary Dental" OR "Temporary Dental Restoration" OR "Temporary Dental Restorations" OR "Dental Fillings, Temporary" OR "Dental Filling, Temporary" OR "Filling, Temporary Dental" OR "Temporary Dental Filling" OR "Fillings, Temporary Dental" OR "Temporary Dental Fillings" OR "Dental Prosthesis, Temporary" OR "Dental Prostheses, Temporary" OR "Prosthesis, Temporary Dental" OR "Temporary Dental Prostheses" OR "Temporary Dental Prosthesis" OR "Dentistry, Operative" OR "Operative Dentistry" OR "dental restoration" OR "restorative dentistry" OR "Dental Restoration Repair" OR "Dental Restoration Repairs" OR "Repair, Dental Restoration" OR "Repairs, Dental Restoration" OR "Restoration Repair, Dental" OR "Restoration Repairs, Dental" OR "Dental Restoration Failure" OR "Failure, Dental Restoration" OR "Restoration Failure, Dental" OR "Restoration Failures, Dental" OR "Dental Restoration Failures" OR "Failures, Dental Restoration" OR Crowns OR Crown OR "Dental Crowns" OR "Dental Crown" OR "Crown, Dental" OR "Crowns, Dental" OR "tooth crown" OR "Dental Veneers" OR "Veneer, Dental" OR "Veneers, Dental" OR "Dental Laminates" OR "Dental Laminate" OR "Laminate, Dental" OR "Laminates, Dental" OR "Dental Veneer" OR "tooth veneer" OR "Dental Restoration Wear" OR "Restoration Wear, Dental" OR "Wear, Dental Restoration" OR "Restoration Occlusal Wear" OR "Wear, Restoration Occlusal" OR "Occlusal Wear, Restoration" OR "Denture Wear" OR "Wear, Denture" OR "Dental Prosthesis" OR "Dental Prostheses" OR "Prostheses, Dental" OR "Prosthesis, Dental" OR "Post and Core Technique" OR "Post and Core Technic" OR "Post-Core Technic" OR "Post-Core Technics" OR "Technic, Post-Core" OR "Technics, Post-Core" OR "Post Technique" OR "Post Techniques" OR "Technique, Post" OR "Techniques, Post" OR "Post Technic" OR "Post Technics" OR "Technic, Post" OR "Technics, Post" OR "Dental Dowel" OR "Dowel, Dental" OR "Dowels, Dental" OR "Dental Dowels" OR "Dental Prosthesis Repair" OR "Dental Prosthesis Repairs" OR "Prosthesis Repair, Dental" OR "Prosthesis Repairs, Dental" OR "Repair, Dental Prosthesis" OR "Repairs, Dental Prosthesis" OR "Dental Prosthesis Retention" OR "Denture Retention")<br><br>#2 TITLE-ABS-KEY("Systematic Reviews as Topic" OR "Systematic Review as Topic" OR "Reviews Systematic as Topic" OR "Systematic Review" OR "Review, Systematic" "systematic reviews")<br><br>#3 TITLE-ABS-KEY("Meta-Analysis" OR "Meta-Analysis as Topic" OR "Meta Analysis as Topic" OR "Data Pooling" OR "Data Poolings" OR "Overviews, Clinical Trial" OR "Clinical Trial Overviews" OR "Clinical Trial Overview" OR "Overview, Clinical Trial" OR "Network Meta-Analysis" OR "Meta-Analyses, Network" OR "Meta-Analysis, Network" OR "Network Meta Analysis" OR "Network Meta-Analyses" OR "Multiple Treatment Comparison Meta-Analysis" OR "Multiple Treatment Comparison Meta Analysis" OR "Mixed Treatment Meta-Analysis" OR "Meta-Analyses, Mixed Treatment" OR

	"Meta-Analysis, Mixed Treatment" OR "Mixed Treatment Meta Analysis" OR "Mixed Treatment Meta-Analyses" OR "meta analysis" OR "analysis, meta" OR metaanalysis OR metaanalyses)<br><br>#1 AND #2 AND #3
Web of science 1416	**#1:** TS=((Dentistry, Operative) OR (Operative Dentistry) OR (Dental Restoration, Permanent) OR (Restorations, Permanent Dental) OR (Permanent Dental Restorations) OR (Restoration, Permanent Dental) OR (Dental Restorations, Permanent) OR (Permanent Dental Restoration) OR (Dental Permanent Fillings) OR (Fillings, Permanent Dental) OR (Permanent Dental Fillings) OR (Permanent Fillings, Dental) OR (Permanent Filling, Dental) OR (Dental Filling, Permanent) OR (Dental Permanent Filling) OR (Filling, Dental Permanent) OR (Filling, Permanent Dental) OR (Permanent Dental Filling) OR (Fillings, Dental Permanent) OR (Dental Fillings, Permanent) OR (dental restoration) OR (restorative dentistry) OR (Dental Restoration Repair) OR (Dental Restoration Repairs) OR (Repair, Dental Restoration) OR (Repairs, Dental Restoration) OR (Restoration Repair, Dental) OR (Restoration Repairs, Dental) OR (Dental Restoration Failure) OR (Failure, Dental Restoration) OR (Restoration Failure, Dental) OR (Restoration Failures, Dental) OR (Dental Restoration Failures) OR (Failures, Dental Restoration) OR (Crowns) OR (Crown) OR (Dental Crowns) OR (Dental Crown) OR (Crown, Dental) OR (Crowns, Dental) OR (tooth crown) OR (Dental Veneers) OR (Veneer, Dental) OR (Veneers, Dental) OR (Dental Laminates) OR (Dental Laminate) OR (Laminate, Dental) OR (Laminates, Dental) OR (Dental Veneer) OR (tooth veneer))<br>**#2:** TS=((Meta-Analysis) OR (Meta-Analysis as Topic) OR (Meta Analysis as Topic) OR (Data Pooling) OR (Data Poolings) OR (Overviews, Clinical Trial) OR (Clinical Trial Overviews) OR (Clinical Trial Overview) OR (Overview, Clinical Trial) OR (Network Meta-Analysis) OR (Meta-Analyses, Network) OR (Meta-Analysis, Network) OR (Network Meta Analysis) OR (Network Meta-Analyses) OR (Multiple Treatment Comparison Meta-Analysis) OR (Multiple Treatment Comparison Meta Analysis) OR (Mixed Treatment Meta-Analysis) OR (Meta-Analyses, Mixed Treatment) OR (Meta-Analysis, Mixed Treatment) OR (Mixed Treatment Meta Analysis) OR (Mixed Treatment Meta-Analyses) OR (meta analysis) OR (analysis, meta) OR (metaanalysis) OR (metaanalyses))<br>**#3:** TS=((Systematic Reviews as Topic) OR (Systematic Review as Topic) OR (Reviews Systematic as Topic) OR (Systematic Review) OR (Review, Systematic) OR (systematic literature review) OR (systematic scoping review) OR (systematic narrative review) OR (systematic qualitative review) OR (systematic evidence review) OR (systematic quantitative review) OR (systematic meta-review) OR (systematic critical review) OR (systematic mixed studies review) OR (systematic mapping review) OR (systematic cochrane review) OR (systematic integrative review) OR (systematic reviews))<br>#1 AND #2 AND #3

**Cochrane Collaboration Library 118**	**#1** ("Dental Restoration, Permanent" OR "Dental Restorations, Permanent" OR "Permanent Dental Restoration" OR "Permanent Dental Restorations" OR "Restoration, Permanent Dental" OR "Restorations, Permanent Dental" OR "Dental Permanent Fillings" OR "Fillings, Dental Permanent" OR "Fillings, Permanent Dental" OR "Permanent Dental Fillings" OR "Permanent Fillings, Dental" OR "Dental Fillings, Permanent" OR "Dental Filling, Permanent" OR "Dental Permanent Filling" OR "Filling, Dental Permanent" OR "Filling, Permanent Dental" OR "Permanent Dental Filling" OR "Permanent Filling, Dental" OR "Dental Restoration, Temporary" OR "Restoration, Temporary Dental" OR "Restorations, Temporary Dental" OR "Temporary Dental Restoration" OR "Temporary Dental Restorations" OR "Dental Fillings, Temporary" OR "Dental Filling, Temporary" OR "Filling, Temporary Dental" OR "Temporary Dental Filling" OR "Fillings, Temporary Dental" OR "Temporary Dental Fillings" OR "Dental Prosthesis, Temporary" OR "Dental Prostheses, Temporary" OR "Prosthesis, Temporary Dental" OR "Temporary Dental Prostheses" OR "Temporary Dental Prosthesis" OR "Dentistry, Operative" OR "Operative Dentistry" OR "dental restoration" OR "restorative dentistry" OR "Dental Restoration Repair" OR "Dental Restoration Repairs" OR "Repair, Dental Restoration" OR "Repairs, Dental Restoration" OR "Restoration Repair, Dental" OR "Restoration Repairs, Dental" OR "Dental Restoration Failure" OR "Failure, Dental Restoration" OR "Restoration Failure, Dental" OR "Restoration Failures, Dental" OR "Dental Restoration Failures" OR "Failures, Dental Restoration" OR Crowns OR Crown OR "Dental Crowns" OR "Dental Crown" OR "Crown, Dental" OR "Crowns, Dental" OR "tooth crown" OR "Dental Veneers" OR "Veneer, Dental" OR "Veneers, Dental" OR "Dental Laminates" OR "Dental Laminate" OR "Laminate, Dental" OR "Laminates, Dental" OR "Dental Veneer" OR "tooth veneer" OR "Dental Restoration Wear" OR "Restoration Wear, Dental" OR "Wear, Dental Restoration" OR "Restoration Occlusal Wear" OR "Wear, Restoration Occlusal" OR "Occlusal Wear, Restoration" OR "Denture Wear" OR "Wear, Denture" OR "Dental Prosthesis" OR "Dental Prostheses" OR "Prostheses, Dental" OR "Prosthesis, Dental" OR "Post and Core Technique" OR "Post and Core Technic" OR "Post-Core Technic" OR "Post-Core Technics" OR "Technic, Post-Core" OR "Technics, Post-Core" OR "Post Technique" OR "Post Techniques" OR "Technique, Post" OR "Techniques, Post" OR "Post Technic" OR "Post Technics" OR "Technic, Post" OR "Technics, Post" OR "Dental Dowel" OR "Dowel, Dental" OR "Dowels, Dental" OR "Dental Dowels" OR "Dental Prosthesis Repair" OR "Dental Prosthesis Repairs" OR "Prosthesis Repair, Dental" OR "Prosthesis Repairs, Dental" OR "Repair, Dental Prosthesis" OR "Repairs, Dental Prosthesis" OR "Dental Prosthesis Retention" OR "Denture Retention")<br><br>**#2** ("Meta-Analysis" OR "Meta-Analysis as Topic" OR "Meta Analysis as Topic" OR "Data Pooling" OR "Data Poolings" OR "Overviews, Clinical Trial" OR "Clinical Trial Overviews" OR "Clinical Trial Overview" OR "Overview, Clinical Trial" OR "Network Meta-Analysis" OR "Meta-Analyses, Network" OR "Meta-Analysis, Network" OR "Network Meta Analysis" OR "Network Meta-Analyses" OR "Multiple Treatment Comparison Meta-Analysis" OR "Multiple Treatment Comparison Meta Analysis" OR "Mixed Treatment Meta-Analysis" OR "Meta-Analyses, Mixed Treatment" OR "Meta-Analysis, Mixed Treatment" OR "Mixed Treatment Meta Analysis" OR "Mixed Treatment Meta-Analyses" OR "meta analysis" OR "analysis, meta" OR "metaanalysis" OR "metaanalyses")<br><br>**#3** ("Systematic Reviews as Topic" OR "Systematic Review as Topic" OR "Reviews Systematic as Topic" OR "Systematic Review" OR "Review, Systematic" "systematic reviews")<br><br>#1 AND #2 AND #3
