# Supplementary material for: Spin and reporting quality in systematic reviews of nonrandomized studies in restorative dentistry: a meta-research study
Source: Braz Oral Res. 2026 Nov 9;40:e094. doi: 10.1590/1807-3107bor-2026.vol40.094 (PMC13750809; doi:10.1590/1807-3107bor-2026.vol40.094)
Supplement: Supplementary file 3 [file 1807-3107-bor-40-e094-suppl03.pdf]

Supplementary Material 3. Reports excluded and reasons.

Reports excluded and reasons (n= 312):

Not a systematic review (n= 25):

[1] Alhareky M, Tavares M. Amalgam vs Composite Restoration, Survival, and Secondary Caries. J Evid Based Dent Pract. 2016 Jun;16(2):107-9. doi: 10.1016/j.jebdp.2016.05.001. Epub 2016 May 14. PMID: 27449837.

[2] Baghdadi ZD. Preservation-based approaches to restore posterior teeth with amalgam, resin or a combination of materials. Am J Dent 2002;15:54-65.

[3] Beuer F, Pieralli S, Spitznagel F, Zlatarić DK, Bleiel H, Gierthmühlen P, Schnutenhaus S, Strub J, Rutten L, Rutten P, Jäggi M, Yilmaz B, Reuss JM, Mallat-Callis E, Jiménez J, Martinez-de-Fuentes R, Ortiz MA, Gómez-Polo M, Klein P. Survival and Complication Rates of Ceramic Partial Coverage Restorations (PCRs) and Ceramic Laminate Veneers Made of Different Types of Ceramics. Consensus Statement From SSRD, SEPES, and PROSEC Conference on Minimally Invasive Restorations. J Esthet Restor Dent. 2025 Mar;37(3):664-668. doi: 10.1111/jerd.13418.

[4] Borbely J. Low-quality Evidence Suggests Digital Impressions (DIs) and Conventional Impressions Are Comparable for Marginal and Internal Fit, Very-low Quality Evidence for Interproximal and Occlusal Contacts is Insufficient to Draw Conclusion and There Is No Evidence Regarding Survival of Full-coverage Restorations. J Evid Based Dent Pract. 2019 Dec;19(4):101347. doi: 10.1016/j.jebdp.2019.101347.

[5] Carvalho RM, Manso AP. Biodegradation of Resin-Dentin Bonds: a Clinical Problem? Curr Oral Heal Rep 2016;3:229-33. https://doi.org/10.1007/s40496-016-0104-0

[6] Davies H, Ahmed SH, Edwards D. Metal vs fibre posts - which is clinically superior for the restoration of endodontically treated teeth? Evid Based Dent 2021;22:162-3. https://doi.org/10.1038/s41432-021-0210-6

[7] Ellakwa A, Goh D. The marginal accuracy of lithium disilicate single crowns is comparable when made from conventional or digital (direct or indirect) workflows. J Evid Based Dent Pract. 2023 Dec;23(4):101914. doi: 10.1016/j.jebdp.2023.101914.

[8] Khan A.S., Syed M., Khurshid Z., Imran E. Fiber-reinforced composites in dentistry – An insight into adhesion aspects of the material and the restored tooth construct. 2023. https://doi.org/10.1016/j.dental.2022.12.003

[9] Murchie B, Jiwan N, Edwards D. What are the success rates of anterior restorations used in localised wear cases? Evid Based Dent. 2025 Mar;26(1):54-56. doi: 10.1038/s41432-025-01112-z. Epub 2025 Feb 1. PMID: 39893261; PMCID: PMC11953042.

[10] Lamperti ST, Wolleb K, Hämmerle CHF, Jung RE, Hüsler J, Thoma DS. Cemented versus screw-retained zirconia-based single-implant restorations: 5-year results of a randomized controlled clinical trial. Clin Oral Implants Res. 2022 Apr;33(4):353-361. doi: 10.1111/clr.13895.

[11] Manso AP, Carvalho RM. Dental Cements for Luting and Bonding Restorations: Self-Adhesive Resin Cements. Dent Clin North Am 2017;61:821-34. https://doi.org/10.1016/j.cden.2017.06.006

[12] Mickenautsch S. High-viscosity glass-ionomer cements for direct posterior tooth restorations in permanent teeth: The evidence in brief. J Dent 2016;55:121-3. https://doi.org/10.1016/j.jdent.2016.07.008

[13] Oh WS. Cement Type (Adhesive vs. Conventional) May Not Affect Clinical Performance of Zirconia and Lithium Disilicate Tooth-Supported Crowns. J Evid Based Dent Pract. 2020 Jun;20(2):101415. doi: 10.1016/j.jebdp.2020.101415.

[14] Pecie R, Krejci I, García-Godoy F, Bortolotto T. Noncarious cervical lesions (NCCL) - A clinical concept based on the literature review. Part 2: Restoration. Am J Dent 2011;24:183-92. PMID: 21874940.

[15] Perdigão J, Swift Jr EJ. Universal Adhesives. J Esthet Restor Dent 2015;27:331-4. https://doi.org/10.1111/jerd.12185

[16] Pini NP, Aguiar FHB, Leite Lima DAN, Lovadino JR, Suga Terada RS, Pascotto RC. Advances in dental veneers: Materials, applications, and techniques. Clin Cosmet Investig Dent 2012;4:9-16. https://doi.org/10.2147/CCIDE.S19650

[17] Sadowsky SJ. An overview of treatment considerations for esthetic restorations: A review of the literature. J Prosthet Dent 2006;96:433-42. https://doi.org/10.1016/j.prosdent.2006.09.018

[18] Schwendicke F. Incomplete dental caries restoration: a systematic review and meta-analysis. Z Evid Fortbild Qual Gesundhwes 2015;109:56-8. https://doi.org/10.1016/j.zefq.2014.12.009

[19] Soleimani F, Jalali H, Mostafavi AS, Zeighami S, Memarian M. Retention and Clinical Performance of Zirconia Crowns: A Comprehensive Review. Int J Dent 2020;2020. https://doi.org/10.1155/2020/8846534

[20] Tsujimoto A, Barkmeier WW, Fischer NG, Nojiri K, Nagura Y, Takamizawa T, et al. Wear of resin composites: Current insights into underlying mechanisms, evaluation methods and influential factors. Jpn Dent Sci Rev 2018;54:76-87. https://doi.org/10.1016/j.jdsr.2017.11.002

[21] Tang W, Wu Y, Smales RJ. Identifying and Reducing Risks for Potential Fractures in

Endodontically Treated Teeth. J Endod 2010;36:609-17. https://doi.org/10.1016/j.joen.2009.12.002

[22] Tezvergil-Mutluay A, Pashley D, Mutluay MM. Long-Term Durability of Dental Adhesives. Curr Oral Heal Rep 2015;2:174-81. https://doi.org/10.1007/s40496-015-0070-y

[23] Tjäderhane L, Tezvergil-Mutluay A. Performance of Adhesives and Restorative Materials After Selective Removal of Carious Lesions: Restorative Materials with Anticaries Properties. Dent Clin North Am 2019;63:715-29. https://doi.org/10.1016/j.cden.2019.05.001

[24] Van Meerbeek B, Peumans M, Poitevin A, Mine A, Van Ende A, Neves A, et al. Relationship between bond-strength tests and clinical outcomes. Dent Mater 2010;26. https://doi.org/10.1016/j.dental.2009.11.148

[25] Zhang X, Pei X, Pei X, Wan Q, Chen J, Wang J. Success and Complication Rates of Root-Filled Teeth Restored with Zirconia Posts: A Critical Review. Int J Prosthodont. 2019 Sep/Oct;32(5):411-419. doi: 10.11607/ijp.6179.

Did not perform meta-analysis (n= 86):

[1] Ástvaldsdóttir Á, Dagerhamn J, Van Dijken JW V, Naimi-Akbar A, Sandborgh-Englund G, Tranæus S, et al. Longevity of posterior resin composite restorations in adults - A systematic review. J Dent 2015;43(8):934–54. https://doi.org/10.1016/j.jdent.2015.05.001

[2] Abad-Coronel C, Naranjo B, Valdiviezo P. Adhesive Systems Used in Indirect Restorations Cementation: Review of the Literature. Dent J. 2019;7(3).

[3] Abdullah AO, Muhammed FK, Zheng B, Liu Y. An overview of extrinsic tooth bleaching and its impact on oral restorative materials. World J Dent [Internet]. 2017;8(6):503–10. https://doi.org/10.5005/jp-journals-10015-1494.

[4] Abduo J, Sambrook RJ. Longevity of ceramic onlays: A systematic review. J Esthet Restor Dent [Internet]. 2018;30(3):193–215. https://doi.org/10.1111/jerd.12384.

[5] Agossa K, Godel G, Dubar M, S.Y. K, Behin P, Delcourt-Debruyne E. Does Evidence Support a Combined Restorative Surgical Approach for the Treatment of Gingival Recessions Associated With Noncarious Cervical Lesions? J Evid Based Dent Pract [Internet]. 2017;17(3):226–38. https://doi.org/10.1016/j.jebdp.2017.04.001.

[6] Ahmed KE, Li KY, Murray CA. Longevity of fiber-reinforced composite fixed partial dentures (FRC FPD)-Systematic review. J Dent. 2017 Jun;61:1-11. doi: 10.1016/j.jdent.2016.08.007.

[7] Ahmed KE, Murbay S. Survival rates of anterior composites in managing tooth wear: systematic review. J Oral Rehabil. 2016 Feb;43(2):145-53. doi: 10.1111/joor.12360. Epub 2015 Oct 5. PMID: 26440584.

[8] AlJazairy YH. Survival Rates for Porcelain Laminate Veneers: A Systematic Review. Eur J Dent. 2021 May;15(2):360–8.

[9] Alhajj et al. Fracture Resistance of Titanium and Fiber Dental Posts: A Systematic Review and Meta-Analysis. J Prosthodont 2022 Jun;31(5):374-384. https://doi.org/10.1111/jopr.13428.

[10] Alghauli MA, Alqutaibi AY, Borzangy S. Clinical benefits of immediate dentin sealing: A systematic review and meta-analysis. J Prosthet Dent. 2025 Oct;134(4):996-1004. doi: 10.1016/j.prosdent.2024.03.014. Epub 2024 Apr 3. PMID: 38570282.

[11] Alhamdan MM, Alghuwainem N, Alharbi M, Hummady S. Clinical Outcome of Indirect Bonded Porcelain Restoration Versus Full-Coverage Crown on Endodontically Treated Teeth in Posterior Areas: A Systematic Review. Cureus. 2024 Sep 24;16(9):e70116. doi: 10.7759/cureus.70116. PMID: 39449898; PMCID: PMC11501469.

[12] Alhumaidan et al. Clinical performance of indirect restorations with cervical margin relocation in posterior teeth: A systematic review. Dentistry Review 2022; 2(1): 100034. https://doi.org/10.1016/j.dentre.2022.100034

[13] Alraheam IA, Ngoc CN, Wiesen CA, Donovan TE. Five-year success rate of resin-bonded fixed partial dentures: A systematic review. J Esthet Restor Dent. 2019 Jan;31(1):40-50. doi: 10.1111/jerd.12431.

[14] Alvanforoush N, Palamara J, Wong RH, Burrow MF. Comparison between published clinical success of direct resin composite restorations in vital posterior teeth in 1995–2005 and 2006–2016 periods. Aust Dent J [Internet]. 2017;62(2):132–45. https://doi.org/10.1111/adj.12487.

[15] Alqutaibi AY, Al-Gabri RS, Al-Zaghruri AS, Farghal AE, Alnazzawi AA, Alghauli MA. Clinical performance, accuracy, and physical-mechanical properties of 3D-printed removable partial denture metal frameworks compared with conventionally and partially digitally produced frameworks: A systematic review. J Prosthodont Res. 2025 Sep 3. doi: 10.2186/jpr.JPR_D_25_00028.

[16] Ansari SH, Alfaqeeh AA, Al Buryk A, Alfaqeeh SA, Almusharraf AYA, Aljarullah AHN. Indications and Success Rate of Endo Crowns - A Systematic Review. J Evol Med Dent Sci 2020;9(43):3247–51.

[17] Angerame D, De Biasi M. Do Nanofilled/Nanohybrid Composites Allow for Better Clinical Performance of Direct Restorations Than Traditional Microhybrid Composites? A Systematic Review. Oper Dent 2018;43(4) –209.

[18] Apte A, et al. Evaluation of success rate of Zirconia based restorations: A systematic review. J Clin Exp Dent 2022;14(9): e756–e761. https://doi.org/10.4317/jced.59711.

[19] Aurélio IL, Fraga S, Rippe MP, Valandro LF. Are posts necessary for the restoration of root filled teeth with limited tissue loss? A structured review of laboratory and clinical studies. Int Endod J 2016;49(9):827–35. https://doi.org/10.1111/iej.12538.

[20] Aziz A, El-Mowafy O, Paredes S. Clinical outcomes of lithium disilicate glass-ceramic crowns fabricated with CAD/CAM technology: A systematic review. Dent Med Probl. 2020;57(2):197–206. https://doi.org/10.17219/dmp/115522.

[21] Benli N, et al. Clinical performance of lithium disilicate and zirconia CAD/CAM crowns using digital impressions: A systematic review. Prim Dent J 2022;11(4):71-76. https://doi.org/10.1177/20501684221132941.

[22] Bhuva B, Giovarruscio M, Rahim N, Bitter K, Mannocci F. The restoration of root filled teeth: a review of the clinical literature. Int Endod J 2021;54(4):509–35. https://doi.org/10.1111/iej.13438

[23] Blum IR, Özcan M. Reparative Dentistry: Possibilities and Limitations. Curr Oral Heal Reports 2018;5(4):264–9. https://doi.org/10.1007/s40496-018-0191-1.

[24] Boitelle P, Mawussi B, Tapie L, Fromentin O. A systematic review of CAD/CAM fit restoration evaluations. J Oral Rehabil 2014;41(11):853–74. https://doi.org/10.1111/joor.12205.

[25] Brian HCM, Lam OL, Jagannathan N, Botelho MG. A systematic review of amalgam bonded restorations: In vitro and clinical findings. J Contemp Dent Pract 2018;19(8):1012–24.

[26] Conejo J, Nueesch R, Vonderheide M, Blatz MB. Clinical Performance of All-Ceramic Dental Restorations. Curr Oral Heal Reports 2017;4(2):112–23. https://doi.org/10.1007/s40496-017-0132-4.

[27] Conrad HJ, Seong WJ, Pesun GJ. Current ceramic materials and systems with clinical recommendations: A systematic review. J Prosthet Dent. 2007;98(5):389–404. https://doi.org/10.1016/S0022-3913(07)60124-3.

[28] Costa TS, Brandão RMR, Farias Vajgel BC, SoutoMaior JR. CAD-CAM glass fiber compared with conventional prefabricated glass fiber posts: A systematic review. J Prosthet Dent. 2024 Aug;132(2):337-342. doi: 10.1016/j.prosdent.2022.01.007.

[29]Çötert I, Çötert H. Survival of Partial Laminate Veneers and Categorical Covariates Affecting the Survival: A Systematic Review . Open Dent J, 2023; 17: e187421062308280. http://dx.doi.org/10.2174/0118742106254225230921100826

[30]da Mota Martins V, Paranhos LR, de Oliveira MN, Maia LC, Machado AC, Santos-Filho PCF. Does the addition of chlorhexidine to glass ionomer cements influence its antimicrobial effect and survival rate? A systematic review. Eur Arch Paediatr Dent 2022; 23(3):365-379. https://doi.org/10.1007/s40368-022-00699-6.

[31]de Carvalho MA, Lazari PC, Gresnigt M, Del Bel Cury AA, Magne P. Current options concerning the endodontically-treated teeth restoration with the adhesive approach. Braz Oral Res 2018; 32:147–58. https://doi.org/10.1590/1807-3107bor-2018.vol32.0074

[32]de Carvalho Martins BM, Nogueira Leal da Silva EJ, Masterson Tavares Pereira Ferreira D, Rodrigues Reis K, da Silva Fidalgo TK. Longevity of defective direct restorations treated by minimally invasive techniques or complete replacement in permanent teeth: A systematic review. J Dent 2018; 78:22–30. https://doi.org/10.1016/j.jdent.2018.09.001

[33]de Menezes AJO, do Nascimento Barbosa L, Leite JVC, Barbosa LMM, Montenegro RV, Dantas RVF, de Souza GM, de Andrade AKM, Lima RBW. Clinical Outcomes of Bulk-Fill Resin Composite Restorations: A 10-Year Mapping Review and Evidence Gap Map. J Esthet Restor Dent. 2025 Apr;37(4):920-933. doi: 10.1111/jerd.13339.

[34]Demarco FF, Collares K, Coelho-De-Souza FH, Correa MB, Cenci MS, Moraes RR, et al. Anterior composite restorations: A systematic review on long-term survival and reasons for failure. Dent Mater 2015;31(10):1214–24. . https://doi.org/10.1016/j.dental.2015.07.005.

[35]Denis R, Marty M, Esclassan R, Noirrit-Esclassan E, Canceill T. Description and Durability of the Various Direct Restoration Techniques in Molar-Incisor Hypomineralization: A Systematic Review. Eur J Prosthodont Restor Dent. 2025 Feb 28;33(1):113-122. doi: 10.1922/EJPRD_2760Denis10.

[36]Dionysopoulos D. Effect of digluconate chlorhexidine on bond strength between dental adhesive systems and dentin: A systematic review. J Conserv Dent 2016;19(1):11–6. https://doi.org/10.4103/0972-0707.173185.

[37]Fathy H, Hamama HH, El-Wassefy N, Mahmoud SH. Clinical performance of resin-matrix ceramic partial coverage restorations: a systematic review. Clin Oral Investig 2022;26(5):3807-3822. https://doi.org/10.1007/s00784-022-04449-2.

[38]Ferracane JL, Lawson NC. Probing the hierarchy of evidence to identify the best strategy for placing class II dental composite restorations using current materials. J Esthet Restor Dent 2021;33(1):39–50. https://doi.org/10.1111/jerd.12686.

[39]Ferrari M, Vichi A, Zarone F. Zirconia abutments and restorations: From laboratory to clinical investigations. Dent Mater 2015;31(3)–76. https://doi.org/10.1016/j.dental.2014.11.015.

[40]Fernández E, Díaz L, Bersezio C, Martín J, Cabello R, Angel P. Efficacy of Short Fiber-Reinforced Composite Resins in MOD and Endodontically Treated Posterior Teeth: A Systematic Review. J Esthet Restor Dent. 2025 Oct 16. doi: 10.1111/jerd.70046. Epub ahead of print. PMID: 41102600.

[41]Guess PC, Schultheis S, Bonfante EA, Coelho PG, Ferencz JL, Silva NRFA. All-ceramic systems: Laboratory and clinical performance. Dent Clin North Am 2011;55(2):333–52. https://doi.org/10.1016/j.cden.2011.01.005.

[42]Goh D, Ruprai S, Singh Khehra A, Cheng M, Ellakwa A. The Reported Complications of Milled Ceramic Anterior Veneers with at Least One Year Follow Up: A Systematic Review. Eur J Prosthodont Restor Dent. 2025 Aug 29;33(3):277-286. doi: 10.1922/EJPRD_2846Goh10. PMID: 40497595.

[43]Gonzaga CC, et al. Do tooth-supported zirconia restorations present more technical failures related to fracture or loss of retention? Systematic review and meta-analysis. Clin Oral Investig 2022;26(8):5129-5142. https://doi.org/10.1007/s00784-022-04573-z.

[44]Hardan L, Mancino D, Bourgi R, Cuevas-Suárez CE, Lukomska-Szymanska M, Zarow M, Jakubowicz N, Zamarripa-Calderón JE, Kafa L, Etienne O, Reitzer F, Kharouf N, Haïkel Y. Treatment of Tooth Wear Using Direct or Indirect Restorations: A Systematic Review of Clinical Studies. Bioengineering (Basel). 2022 Jul 27;9(8):346. doi: 10.3390/bioengineering9080346.

[45]Hayashi M, Wilson NH, Yeung CA, et al. Systematic review of ceramic inlays. 2003. In: Database of Abstracts of Reviews of Effects (DARE): Quality-assessed Reviews [Internet]. York (UK): Centre for Reviews and Dissemination (UK); 1995-. Available from: https://www.ncbi.nlm.nih.gov/books/NBK70287/

[46]Hayes M, Brady P, Burke FM, Allen PF. Failure rates of class V restorations in the management of root caries in adults - a systematic review. Gerodontology. 2016 Sep;33(3):299–307. https://doi.org/10.1111/ger.12173.

[47]Heintze SD, Loguercio AD, Hanzen TA, Reis A, Rousson V. Clinical efficacy of resin-based direct posterior restorations and glass-ionomer restorations - An updated meta-analysis of clinical outcome parameters. Dent Mater 2022 ;38(5): e109-e135. https://doi.org/10.1016/j.dental.2021.10.018.

[48]Heintze SD. Systematic reviews: I. The correlation between laboratory tests on marginal quality and bond strength. II. The correlation between marginal quality and clinical

outcome. J Adhes Dent 2007;9(35th Annual Meeting of the American-Association-for-Dental-Research/American-Dental-Education-Association):77–106. https://doi.org/10.3290/j.jad.a13084.

[49]Heintze SD, Rousson V. Survival of zirconia- and metal-supported fixed dental prostheses: a systematic review. Int J Prosthodont. 2010 Nov-Dec;23(6):493-502. PMID: 21209982.

[50]Heydecke G, Peters MC. The restoration of endodontically treated, single-rooted teeth with cast or direct posts and cores: a systematic review. J Prosthet Dent. 2002 Apr;87(4):380–6. https://doi.org/10.1067/mpr.2002.122578.

[51]Hickel R, Brüshaver K, Ilie N. Repair of restorations - Criteria for decision making and clinical recommendations. Dent Mater 2013;29(1):28–50. https://doi.org/10.1016/j.dental.2012.07.018.

[52]Josic U, Maravic T, Mazzitelli C, Radovic I, Jacimovic J, Del Bianco F, et al. Is clinical behavior of composite restorations placed in non-carious cervical lesions influenced by the application mode of universal adhesives? A systematic review and meta-analysis. Dent Mater 2021 ;37(11):e503-e521. https://doi.org/10.1016/j.dental.2021.06.014.

[53]Kanzow P, Wiegand A, Schwendicke F, Göstemeyer G. Same, same, but different? A systematic review of protocols for restoration repair. J Dent 2019; 86:1–16. https://doi.org/10.1016/j.jdent.2019.05.001.

[54]Khabadze Z, Mordanov O, Taraki F, Magomedov O, Kuznetsova A, Solimanov S, et al. Effects of the ferrule design on fracture resistance to endodontically-treated teeth restored with fiber posts: A systematic review. Open Dent 2019;13(1):493–8. https://doi.org/10.5935/2238-8321.20190082.

[55]Khabadze Z, Protskaya A, Mordanov O, Protskiy M, et al. Systematica review of zirconium dioxide restoration decontamination methods after contact with saliva during try-in. Journal of international dental and medical research 2023;16(2):879-882.

[56]Khan AA, Zafar MS, Fareed MA, AlMufareh NA, Alshehri F, AlSunbul H, Lassila L, Garoushi S, Vallittu PK. Fiber-reinforced composites in dentistry - An insight into adhesion aspects of the material and the restored tooth construct. Dent Mater. 2023 Feb;39(2):141-151. doi: 10.1016/j.dental.2022.12.003.

[57]Maroulakos G, Thompson GA, Kontogiorgos ED. Effect of cement type on the clinical performance and complications of zirconia and lithium disilicate tooth-supported crowns: A systematic review. Report of the Committee on Research in Fixed Prosthodontics of the American Academy of Fixed Prosthodontic. J Prosthet Dent 2019 May;121(5):754–65. https://doi.org/10.1016/j.prosdent.2018.07.006.

[58]Maroulakos G, Nagy WW, Stathopoulou PG, Mattheos N, Chronopoulos V. Restoration of the Worn Dentition: Basic Prosthodontic Principles and Current Rehabilitation Techniques. Curr Oral Heal Reports 2017;4(2):124–30. https://doi.org/10.1007/s40496-017-0121-7.

[59]Masarwa N, Mohamed A, Abou-Rabii I, Abu Zaghlan R, Steier L. Longevity of Self-etch Dentin Bonding Adhesives Compared to Etch-and-rinse Dentin Bonding Adhesives: A Systematic Review. J Evid Based Dent Pract. 2016 Jun;16(2):96-106. doi: 10.1016/j.jebdp.2016.03.003. Epub 2016 Mar 29. PMID: 27449836.

[60]Mathew VB, Shamsuddin S, Langaliya A, Rathod PT, Gupta B, Ronsivalle V, Cicciù M, Minervini G. Survivability of endodontically treated cracked tooth: A systematic review. Technol Health Care. 2024;32(4):2023-2037. doi: 10.3233/THC-231993. Retraction in: Technol Health Care. 2025 Nov 11:9287329251390260. doi: 10.1177/09287329251390260. PMID: 38517822.

[61]McLister C, Donnelly M, Cardwell CR, Moore C, O'Neill C, Brocklehurst P, McKenna G. Effectiveness of prosthodontic interventions and survival of remaining teeth in adult patients with shortened dental arches-A systematic review. J Dent. 2018 Nov;78:31-39. doi: 10.1016/j.jdent.2018.02.003. Epub 2018 Feb 21. PMID: 29476794.

[62]Mesko ME, Sarkis-Onofre R, Cenci MS, Opdam NJ, Loomans B, Pereira-Cenci T. Rehabilitation of severely worn teeth: A systematic review. J Dent 2016; 48:9–15. https://doi.org/10.1016/j.jdent.2016.03.006.

[63]Mejáre I, Lingström P, Petersson LG, Holm A-K, Twetman S, Källestål C, et al. Caries-preventive effect of fissure sealants: A systematic review. Acta Odontol Scand 2003;61(6):321–30. https://doi.org/10.1080/00016350310007581.

[64]Michaud et al., 2023. Do onlays and crowns offer similar outcomes to posterior teeth with mesial-occlusal-distal preparations? A systematic review. J Esthet Restor Dent 2024;36(2):295-302. https://doi.org/10.1111/jerd.13114.

[65]Mickenautsch S. Are high-viscosity glass-ionomer cements inferior to silver amalgam as restorative materials for permanent posterior teeth? A Bayesian analysis. BMC Oral Health 2015;15(1):118. https://doi.org/10.1186/s12903-015-0095-6.

[66]Mickenautsch S, Yengopal V. Failure Rate of Direct High-Viscosity Glass-Ionomer Versus Hybrid Resin Composite Restorations in Posterior Permanent Teeth - a Systematic Review. Open Dent J 2015; 9:438–48. https://doi.org/10.2174/1874210601509010438.

[67] Miyazaki T, Nakamura T, Matsumura H, Ban S, Kobayashi T. Current status of zirconia restoration. J Prosthodont Res 2013;57(4):236–61. https://doi.org/10.1016/j.jpor.2013.07.001.

[68] Naaman R, El-Housseiny AA, Alamoudi N. The use of pit and fissure sealants-a literature review. Dent J 2017;5(4). https://doi.org/10.3390/dj5040037

[69] Perdigão J, Araujo E, Ramos RQ, Gomes G, Pizzolotto L. Adhesive dentistry: Current concepts and clinical considerations. J Esthet Restor Dent 2021;33(1):51–68. https://doi.org/10.1111/jerd.12666.

[70] Ruengrungsom C, Palarama JEA, Burrow MF. Comparison of ART and conventional techniques on clinical performance of glass-ionomer cement restorations in load bearing areas of permanent and primary dentitions: A systematic review. J Dent 2018 Nov: 78:1-21. https://doi.org/10.1016/j.jdent.2018.07.008.

[71] Santos MC, Azevedo L, Fonseca P, Viana PC, Araújo F, Villarinho E, Fernandes GVO, Correia A. The Success Rate of the Adhesive Partial Fixed Prosthesis after Five Years: A Systematic Review. Prosthesis. 2023; 5(1):282-294. https://doi.org/10.3390/prosthesis5010021

[72] Sarkis-Onofre R, et al. Performance of Post-retained Single Crowns: A Systematic Review of Related Risk Factors. J Endod 2017;43(2):175-183. https://doi.org/10.1016/j.joen.2016.10.025.

[73] Sequeira-Byron P, Fedorowicz Z, Carter B, Nasser M, Alrowaili EF. Single crowns versus conventional fillings for the restoration of root-filled teeth. Cochrane Database Syst Rev. 2015 Sep 25;2015(9):CD009109. doi: 10.1002/14651858.CD009109.pub3.

[74] Stavropoulou AF, Koidis PT. A systematic review of single crowns on endodontically treated teeth. J Dent 2007;35(10):761–7. https://doi.org/10.1016/j.jdent.2007.06.005.

[75] Suksaphar W, Banomyong D, Jirathanyanatt T, Ngoenwiwatkul Y. Survival rates against fracture of endodontically treated posterior teeth restored with full-coverage crowns or resin composite restorations: a systematic review. Restor Dent Endod. 2017 Aug;42(3):157-167. doi: 10.5395/rde.2017.42.3.157.

[76] Tezulas E, Yildiz C, Evren B, Ozkan Y. Clinical procedures, designs, and survival rates of all-ceramic resin-bonded fixed dental prostheses in the anterior region: A systematic review. J Esthet Restor Dent. 2018 Jul;30(4):307-318. doi: 10.1111/jerd.12389.

[77] Tiu J, Belli R, Lohbauer U. Contemporary CAD/CAM Materials in Dentistry. Curr Oral Heal Reports 2019;6(4):250–6. https://doi.org/10.1007/s40496-019-00235-y.

[78] Tonprasong W, Inokoshi M, Shimizubata M, Yamamoto M, Hatano K, Minakuchi S. Impact of direct restorative dental materials on surface root caries treatment. Evidence based

and current materials development: A systematic review. Jpn Dent Sci Rev. 2022 Nov;58:13-30. doi: 10.1016/j.jdsr.2021.11.004.

[79]Turkyilmaz I, Benli M, Yun S. Evaluation of marginal and internal fit of lithium disilicate and zirconia all-ceramic CAD-CAM crowns using digital impressions: A systematic review. Prim Dent J. 2023 Mar;12(1):88-95. doi: 10.1177/20501684231154323.

[80]Wittneben JG, Wright RF, Weber HP, Gallucci GO. A Systematic Review of the Clinical Performance of CAD/CAM Single-Tooth Restorations. Int J Prosthodont 2009;22(5):466–71. https://doi.org/10.1016/j.jdent.2007.06.005.

[81]Tonprasong et al., 2022. Impact of direct restorative dental materials on surface root caries treatment. Evidence based and current materials development: A systematic review. Jpn Dent Sci Rev 2022; 58:13-30. https://doi.org/10.1016/j.jdsr.2021.11.004.

[82]Turkyilmaz et al., 2023. Evaluation of marginal and internal fit of lithium disilicate and zirconia all-ceramic CAD-CAM crowns using digital impressions: A systematic review. Primary Dental Journal 2023;12(1):88-95. https://doi.org/10.1177/20501684231154323;

[83]Mathew VB, Shamsuddin S, Langaliya A, Rathod PT, Gupta B, Ronsivalle V, Cicciù M, Minervini G. Survivability of endodontically treated cracked tooth: A systematic review. Technol Health Care. 2024;32(4):2023-2037. https://doi.org/10.3233/THC-231993.

[84]de Menezes AJO, do Nascimento Barbosa L, Leite JVC, Barbosa LMM, Montenegro RV, Dantas RVF, de Souza GM, de Andrade AKM, Lima RBW. Clinical Outcomes of Bulk-Fill Resin Composite Restorations: A 10-Year Mapping Review and Evidence Gap Map. J Esthet Restor Dent. 2024 Oct 27. https://doi.org/10.1111/jerd.13339.

[85]Costa TS, Brandão RMR, Farias Vajgel BC, SoutoMaior JR. CAD-CAM glass fiber compared with conventional prefabricated glass fiber posts: A systematic review. J Prosthet Dent. 2024 Aug;132(2):337-342. https://doi.org/10.1016/j.prosdent.2022.01.007.

[86]Alhamdan MM, Alghuwainem N, Alharbi M, Hummady S. Clinical Outcome of Indirect Bonded Porcelain Restoration Versus Full-Coverage Crown on Endodontically Treated Teeth in Posterior Areas: A Systematic Review. Cureus. 2024;16(9):e70116. https://doi.org/10.7759/cureus.70116.

Did not include non-randomized clinical trials in the meta-analysis (n= 63)

[1] Afrashtehfar KI, Ahmadi M, Emami E, Abi-Nader S, Tamimi F. Failure of single-unit restorations on root filled posterior teeth: a systematic review. Int Endod J 2017;50(10):951-66. https://doi.org/ 10.1111/iej.12723.

[2] Alghauli MA, Alqutaibi AY, Wille S, Kern M. Clinical reliability of self-adhesive luting resins compared to other adhesive procedures: A systematic review and meta-analysis. J Dent 2023;129:104394. https://doi.org/ 10.1016/j.jdent.2022.104394.

[3] Alghauli MA, Alqutaibi AY. 3D-printed intracoronal restorations, occlusal and laminate veneers: Clinical relevance, properties, and behavior compared to milled restorations; a systematic review and meta-analysis. J Esthet Restor Dent. 2024 Aug;36(8):1153-1170. doi: 10.1111/jerd.13228.

[4] Alghauli MA, Alqutaibi AY, Borzangy S. Clinical benefits of immediate dentin sealing: A systematic review and meta-analysis. J Prosthet Dent. 2025 Oct;134(4):996-1004. doi: 10.1016/j.prosdent.2024.03.014. Epub 2024 Apr 3. PMID: 38570282.

[5] Alghauli MA, Alqutaibi AY. Mechanical behavior and laboratory survival of thin and ultrathin ceramic crowns: A systematic review and meta-analysis. J Prosthodont. 2025 Oct 13. doi: 10.1111/jopr.70044. Epub ahead of print. PMID: 41084098.

[6] Arbildo-Vega HI, Cruzado-Oliva FH, Coronel-Zubiate FT, Luján-Valencia SA, Meza-Málaga JM, Aguirre-Ipenza R, Echevarria-Goche A, Luján-Urviola E, Castillo-Cornock TB, Serquen-Olano K, Farje-Gallardo CA. Clinical Effectiveness of Ion-Releasing Restorations versus Composite Restorations in Dental Restorations: Systematic Review and Meta-Analysis. Dent J (Basel). 2024 May 24;12(6):158. doi: 10.3390/dj12060158. PMID: 38920859; PMCID: PMC11203382.

[7] Besegato JF, Freire A, Zaniboni JF, Melo GL, Silva AM. Clinical performance of self-adhesive resin composite direct restorations in permanent teeth: a systematic review and meta-analysis. Clin Oral Investig. 2025 Jul 8;29(8):375. doi: 10.1007/s00784-025-06451-w. PMID: 40627224.

[8] Bonfanti-Gris M, Pradies G, Moron-Conejo B, Gil A, Martinez-Rus F. Vertical Versus Horizontal Finishing Lines for Dental Preparations: A Systematic Review With Meta-Analysis. J Esthet Restor Dent. 2024. https://doi.org/ 10.1111/jerd.13360.

[9] Avila WM, Hesse D, Bonifacio CC. Surface Conditioning Prior to the Application of Glass-Ionomer Cement: A Systematic Review and Meta-analysis. J Adhes Dent 2019;21(5):391-9. https://doi.org/ 10.3290/j.jad.a43183.

[10]Cebula M, Göstemeyer G, Krois J, Pitchika V, Paris S, Schwendicke F, Effenberger S. Resin Infiltration of Non-Cavitated Proximal Caries Lesions in Primary and Permanent Teeth: A Systematic Review and Scenario Analysis of Randomized Controlled Trials. J Clin Med 2023;12(2):727. https://doi.org/ 10.3390/jcm12020727.

[11]Chen H, Li T, Ng JPZ, Almeheni L, Li KY, Burrow MF. Clinical performance of zirconia-based tooth-supported fixed dental prostheses: A systematic review and meta-analysis. J Dent. 2024 Dec;151:105382. doi: 10.1016/j.jdent.2024.105382.

[12]Chua DR, Tan BL, Nazzal H, Srinivasan N, Duggal MS, Tong HJ. Outcomes of preformed metal crowns placed with the conventional and Hall techniques: A systematic review and meta-analysis. Int J Paediatr Dent. 2023 Mar;33(2):141-157. doi: 10.1111/ipd.13029.

[13]Correia A, Bresciani E, Borges AB, Pereira DM, Maia LC, Caneppele T. Do Tooth- and Cavity-related Aspects of Noncarious Cervical Lesions Affect the Retention of Resin Composite Restorations in Adults? A Systematic Review and Meta-analysis. Oper Dent. 2020 May/Jun;45(3):E124-E140. doi: 10.2341/19-091-L.

[14]Cuevas-Suárez CE, da Rosa WLO, Lund RG, da Silva AF, Piva E. Bonding Performance of Universal Adhesives: An Updated Systematic Review and Meta-Analysis. J Adhes Dent 2019;21(1):7-26. https://doi.org/ 10.3290/j.jad.a41975.

[15]Dhanraj M, Ranganathan H, Jain AR, Varma ACU. Effect of tooth surface modifications influencing retention in complete veneer restorations - A systematic review and meta analysis. Biomed Pharmacol J 2017;10(2):927-36. https://dx.doi.org/10.13005/bpj/1187.

[16]Fröhlich TT, Rocha RO, Botton G. Does previous application of silver diammine fluoride influence the bond strength of glass ionomer cement and adhesive systems to dentin? Systematic review and meta-analysis. Int J Paediatr Dent 2020;30(1):85-95. https://doi.org/ 10.1111/ipd.12571.

[17]Hadilou M, Dolatabadi A, Ghojazadeh M, Hosseinifard H, Alizadeh Oskuee P, Pournaghi Azar F. Effect of Different Surface Treatments on the Long-Term Repair Bond Strength of Aged Methacrylate-Based Resin Composite Restorations: A Systematic Review and Network Meta-analysis. Biomed Res Int 2022;2022:7708643. https://doi.org/ 10.1155/2022/7708643.

[18]Inokoshi M, De Munck J, Minakuchi S, Van Meerbeek B. Meta-analysis of bonding effectiveness to zirconia ceramics. J Dent Res 2014;93(4):329-34. https://doi.org/ 10.1177/0022034514524228.

[19]Kiuru O, Sinervo J, Vähänikkilä H, Anttonen V, Tjäderhane L. MMP Inhibitors and Dentin Bonding: Systematic Review and Meta-Analysis. Int J Dent 2021;2021:9949699. https://doi.org/ 10.1155/2021/9949699.

[20] de Kuijper MCFM, Cune MS, Özcan M, Gresnigt MMM. Clinical performance of direct composite resin versus indirect restorations on endodontically treated posterior teeth: A systematic review and meta-analysis. J Prosthet Dent. 2023 Sep;130(3):295-306. https://doi.org/ 10.1016/j.prosdent.2021.11.009.

[21] de Geus JL, Maran BM, Cabral KA, Dávila-Sánchez A, Tardem C, Barceleiro MO, Heintze SD, Reis A, Loguercio AD. Clinical Performance of Filled/Nanofilled Versus Nonfilled Adhesive Systems in Noncarious Cervical Lesions: A Systematic Review and Meta-analysis. Oper Dent. 2021 Jan 1;46(1):E34-E59. https://doi.org/10.2341/19-252-L.

[22] Dimova-Gabrovska M, Gusiyska A, Shopova D, Gerdzhikov I, Brusarska1 Y. Modern composites with ceramic fillings for fixed additive prosthetic constructions - are they reliable for permanent application? J of IMAB. 2025 Apr-Jun;31(2):6124-6135. https://doi.org/ 10.5272/jimab.2025312.6124]

[23] Doshi K, Nivedhitha MS, Solete P, S DPA, Balasubramaniam A, Jacob B, Siddique R. Effect of adhesive strategy of universal adhesives in noncarious cervical lesions - an updated systematic review and meta-analysis. BDJ Open. 2023 Feb 13;9(1):6. https://doi.org/10.1038/s41405-022-00124-6.

[24] Elkaffas AA, Hamama HHH, Mahmoud SH. Do universal adhesives promote bonding to dentin? A systematic review and meta-analysis. Restor Dent Endod. 2018 Jun 18;43(3):e29. https://doi.org/ 10.5395/rde.2018.43.e29.

[25] Elrashid AH, Alshaiji BS, Saleh SA, Zada KA, Baseer MA. Efficacy of Resin Infiltrate in Noncavitated Proximal Carious Lesions: A Systematic Review and Meta-Analysis. J Int Soc Prev Community Dent. 2019 May-Jun;9(3):211-218. https://doi.org/10.4103/jispcd.JISPCD_26_19.

[26] Fang K, Chen K, Shi M, Wang L. Effect of different adhesive systems on dental defects and sensitivity to teeth in composite resin restoration: a systematic review and meta-analysis. Clin Oral Investig. 2023 Jun;27(6):2495-2511. doi: 10.1007/s00784-023-05007-0.

[27] Fehrenbach J, de Soares JLS, do Nascimento Foly JCS, Miotti LL, Münchow EA. Mechanical performance of endocrown restorations in anterior teeth: A systematic review and network meta-analysis. Dent Mater. 2025 Jan;41(1):28-41. doi: 10.1016/j.dental.2024.10.012.

[28] Hadilou M, Dolatabadi A, Ghojazadeh M, Hosseinifard H, Alizadeh Oskuee P, Pournaghi Azar F. Effect of Different Surface Treatments on the Long-Term Repair Bond Strength of Aged Methacrylate-Based Resin Composite Restorations: A Systematic Review and Network Meta-analysis. Biomed Res Int. 2022 Sep 5;2022:7708643. doi: 10.1155/2022/7708643.

[29] Heydecke G, Peters MC. The restoration of endodontically treated, single-rooted teeth with cast or direct posts and cores: a systematic review. J Prosthet Dent. 2002 Apr;87(4):380-6. doi: 10.1067/mpr.2002.123848.

[30] Jardim JS, Ferreira VMF, Fernandes E Oliveira HF, Faé DS, Lemos CAA. Is the use of an intraradicular post essential for reducing failures in restoring endodontically treated teeth? A systematic review and meta-analysis. J Dent. 2025 Aug;159:105739. doi: 10.1016/j.jdent.2025.105739.

[31] Josic U, Mazzitelli C, Maravic T, Radovic I, Jacimovic J, Mancuso E, Florenzano F, Breschi L, Mazzoni A. The influence of selective enamel etch and self-etch mode of universal adhesives' application on clinical behavior of composite restorations placed on non-carious cervical lesions: A systematic review and meta-analysis. Dent Mater 2022;38(3):472-88. https://doi.org/ 10.1016/j.dental.2022.01.002.

[32] Lubowitz JH, Cote MP. Meta-analysis of Nonrandomized Controlled Trials Is Rarely Justified: Systematic Reviews Must Avoid Improper Pooling. Arthroscopy. 2025 Feb;41(2):155-159. doi: 10.1016/j.arthro.2024.09.039.

[33] Kerimova Köse, L., Işık, H., Eyüboğlu, T. F., Özcan, M., Çehreli, S. B., & Arhun, N. (2024). Is the repair bond strength affected when substrate and repair composite are not of the same kind? A systematic review and meta-analysis: Effect of repair composite on repair bond strength. Journal of Adhesion Science and Technology, 38(16), 2923–2940. https://doi.org/10.1080/01694243.2024.2325224

[34] Kölüş T, Uçar AY. A Systematic Review and Meta-Analysis of the Success of Resin Composite Restorations. J Adv Oral Res 2022;13(2):157-66. https://doi.org/ 10.1177/23202068221114979.

[35] Kühnisch J, Bedir A, Lo YF, Kessler A, Lang T, Mansmann U, Heinrich-Weltzien R, Hickel R. Meta-analysis of the longevity of commonly used pit and fissure sealant materials. Dent Mater 2020;36(5) https://doi.org/ 10.1016/j.dental.2020.02.001.

[36] Kunz PVM, Wambier LM, Kaizer MDR, Correr GM, Reis A, Gonzaga CC. Is the clinical performance of composite resin restorations in posterior teeth similar if restored with incremental or bulk-filling techniques? A systematic review and meta-analysis. Clin Oral Investig. 2022 Mar;26(3):2281-2297. https://doi.org/ 10.1007/s00784-021-04337-1.

[37] Lins R, Sebold M, Magno MB, Maia LC, Martins L, Giannini M. Does the Type of Solvent in Dental Adhesives Influence the Clinical Performance of Composite Restorations Placed in Noncarious Cervical Lesions? A Systematic Review and Meta-analysis. Oper Dent. 2020 Sep 1;45(5):E237-E254. https://doi.org/10.2341/19-124-LIT.

[38] Mickenautsch S, Yengopal V, Banerjee A. Atraumatic restorative treatment versus amalgam restoration longevity: a systematic review. Clin Oral Investig 2010;14(3):233-40. https://doi.org/ 10.1007/s00784-0

[39] Manisha J, Srivastava G, Das SS, Tabarak N, Choudhury GK. Accuracy of single-unit ceramic crown fabrication after digital versus conventional impressions: A systematic review and meta-analysis. J Indian Prosthodont Soc 2023;23(2):105-11. https://doi.org/ 10.4103/jips.jips_534_22.

[40] Moussa C, Savard G, Rochefort G, Renaud M, Denis F, Daou MH. Fracture Resistance of Direct versus Indirect Restorations on Posterior Teeth: A Systematic Review and Meta-Analysis. Bioengineering (Basel). 2024 May 24;11(6):536. doi: 10.3390/bioengineering11060536.

[41] Narde, Joshua; Pandurangan, Kiran Kumar; Venugopalan, Suresh; Ahmed, Nabeel. Comparison and Evaluation of Quality and Survival of Indirect Composite Resin and Ceramic in Fixed Restorations: A Systematic Review and Meta-analysis. Journal of International Oral Health 17(1):p 23-32, January-February 2025. https://doi.org/ 10.4103/jioh.jioh_97_24

[42] Narde, Joshua; Pandurangan, Kiran Kumar; Venugopalan, Suresh; Ahmed, Nabeel. Comparison and Evaluation of Quality and Survival of Indirect Composite Resin and Ceramic in Fixed Restorations: A Systematic Review and Meta-analysis. Journal of International Oral Health 17(1):p 23-32, January-February 2025. https://doi.org/ 10.4103/jioh.jioh_97_24

[43] Nogueira IO, Pereira CNB, Abreu LG, Diniz IMA, Magalhães CS, Silveira RRD. Do different protocols affect the success rate or bond strength of glass-ceramics repaired with composite resin? A systematic review and meta-analysis. J Prosthet Dent 2023;S0022-3913(23)00432-8. https://doi.org/ 10.1016/j.prosdent.2023.06.020.

[44] Pachiou A, Zervou E, Tsirogiannis P, Sykaras N. Evaluation of the marginal fit of lithium disilicate single crowns fabricated with the conventional (IPS e.max Press) and CAD/CAM (IPS e.max CAD) methods: a systematic review and meta-analysis. Int J Comput Dent. 2024 Dec 9;27(4):365-377. https://doi.org/10.3290/j.ijcd.b4224643.

[45] Petridis HP, Zekeridou A, Malliari M, Tortopidis D, Koidis P. Survival of ceramic veneers made of different materials after a minimum follow-up period of five years: a systematic review and meta-analysis. Eur J Esthet Dent 2012;7(2):138-52.

[46] Portella FF, Müller R, Zimmer R, Reston EG, Arossi GA. Is immediate dentin sealing a mandatory or optional clinical step for indirect restorations? J Esthet Restor Dent. 2024 Jun;36(6):892-900. doi: 10.1111/jerd.13205.

[47]Prott LS, Pieralli S, Klein P, Spitznagel FA, Ibrahim F, Metzendorf MI, Carrasco-Labra A, Blatz MB, Gierthmuehlen PC. Survival and Complications of Partial Coverage Restorations on Posterior Teeth-A Systematic Review and Meta-Analysis. J Esthet Restor Dent. 2025 Mar;37(3):620-641. doi: 10.1111/jerd.13353.

[48]Puleio F, Fiorillo L, Gorassini F, Iandolo A, Meto A, D'Amico C, Cervino G, Pinizzotto M, Bruno G, Portelli M, Amato A, Lo Giudice R. Systematic Review on White Spot Lesions Treatments. Eur J Dent 2022;16(1):41-8. https://doi.org/ 10.1055/s-0041-1731931.

[49]Rossoni NB, Cavalheiro CP, Casagrande L, Lenzi TL. Influence of the chemomechanical and mechanical carious tissue removal on the risk of restorative failure: a systematic review and meta-analysis. Clin Oral Investig 2022;26(11):6457-67. https://doi.org/ 10.1007/s00784-022-04695-4.

[50]Sanches IB, Metzker TC, Kappler R, Oliveira MV, Carvalho AO, Castor Xisto Lima EM. Marginal adaptation of CAD-CAM and heat-pressed lithium disilicate crowns: A systematic review and meta-analysis. J Prosthet Dent. 2023 Jan;129(1):34-39. doi: 10.1016/j.prosdent.2021.03.021.

[51]Santos AP, Moreira IK, Scarpelli AC, Pordeus IA, Paiva SM, Martins CC. Survival of Adhesive Restorations for Primary Molars: A Systematic Review and Metaanalysis of Clinical Trials. Pediatr Dent. 2016 Oct 15;38(5):370-378.

[52]Santos M, Saadaldin S, Rêgo H, Tayan N, Melo NM, El-Najar M. Clinical Longevity of Complex Direct Posterior Resin Composite and Amalgam Restorations: A Systematic Review and Meta-analysis. Oper Dent. 2025 Sep 23;50(4):E30-E46. doi: 10.2341/24-007-LIT.

[53]Schmidt J, Proesl S, Schulz-Kornas E, Haak R, Meyer-Lueckel H, Campus G, Esteves-Oliveira M. Systematic review and network meta-analysis of restorative therapy and adhesive strategies in root caries lesions. J Dent. 2024 Mar;142:104776. doi: 10.1016/j.jdent.2023.104776. Epub 2023 Nov 17.

[54]Sedrez-Porto JA, Rosa WL, da Silva AF, Münchow EA, Pereira-Cenci T. Endocrown restorations: A systematic review and meta-analysis. J Dent 2016;52:8-14. https://doi.org/ 10.1016/j.jdent.2016.07.005.

[55]Shaalan OO, Abou-Auf E, El Zoghby AF. Clinical evaluation of flowable resin composite versus conventional resin composite in carious and noncarious lesions: Systematic review and meta-analysis. J Conserv Dent 2017;20(6):380-5. https://doi.org/ 10.4103/JCD.JCD_226_17.

[56]Sailer I, Makarov NA, Thoma DS, Zwahlen M, Pjetursson BE. All-ceramic or metal-ceramic tooth-supported fixed dental prostheses (FDPs)? A systematic review of the survival and

complication rates. Part I: Single crowns (SCs). Dent Mater 2015;31(6):603-23. doi: 10.1016/j.dental.2015.02.011. Erratum in: Dent Mater 2016;32(12). https://doi.org/ 10.1016/j.dental.2016.09.032.

[57]Sanches IB, Metzker TC, Kappler R, Oliveira MV, Carvalho AO, Castor Xisto Lima EM. Marginal adaptation of CAD-CAM and heat-pressed lithium disilicate crowns: A systematic review and meta-analysis. J Prosthet Dent 2023;129(1):34-9. https://doi.org/ 10.1016/j.prosdent.2021.03.021.

[58]Solon-de-Mello M, da Silva Fidalgo TK, Dos Santos Letieri A, Masterson D, Granjeiro JM, Monte Alto RV, Maia LC. Longevity of indirect restorations cemented with self-adhesive resin luting with and without selective enamel etching. A Systematic review and meta-analysis. J Esthet Restor Dent. 2019 Jul;31(4):327-337. doi: 10.1111/jerd.12504. Epub 2019 Jun 17. PMID: 31207007.

[59]Tabesh M, Nejatidanesh F, Savabi G, Davoudi A, Savabi O. Marginal Accuracy of Lithium Disilicate Full-Coverage Single Crowns Made by Direct and Indirect Digital or Conventional Workflows: A Systematic Review and Meta-Analysis. J Prosthodont 2022;31(9):744-53. https://doi.org/ 10.1111/jopr.13515.

[60]Tennert C, Suárez Machado L, Jaeggi T, Meyer-Lueckel H, Wierichs RJ. Posterior ceramic versus metal restorations: A systematic review and meta-analysis. Dent Mater 2022;38(10):1623-1632. https://doi.org/ 10.1016/j.dental.2022.08.002.

[61]Veloso SRM, Lemos CAA, de Moraes SLD, do Egito Vasconcelos BC, Pellizzer EP, de Melo Monteiro GQ. Clinical performance of bulk-fill and conventional resin composite restorations in posterior teeth: a systematic review and meta-analysis. Clin Oral Investig. 2019 Jan;23(1):221-233. doi: 10.1007/s00784-018-2429-7.

[62]Worthington HV, Khangura S, Seal K, Mierzwinski-Urban M, Veitz-Keenan A, Sahrmann P, Schmidlin PR, Davis D, Iheozor-Ejiofor Z, Rasines Alcaraz MG. Direct composite resin fillings versus amalgam fillings for permanent posterior teeth. Cochrane Database Syst Rev. 2021 Aug 13;8(8):CD005620. doi: 10.1002/14651858.CD005620.pub3.

[63]Zhu Z, Dong XY, He S, Pan X, Tang L. Effect of Post Placement on the Restoration of Endodontically Treated Teeth: A Systematic Review. Int J Prosthodont. 2015 Sep-Oct;28(5):475-83. doi: 10.11607/ijp.4120.

Other study designs were included in the meta-analysis together with non-randomized clinical trials (n=89):

[1] Adánez MH, Nishihara H, Att W. A systematic review and meta-analysis on the clinical outcome of zirconia implant-restoration complex. J Prosthodont Res. 2018 Oct;62(4):397-406. doi: 10.1016/j.jpor.2018.04.007.

[2] Al-Bermani ASA, Quigley NP, Ha WN. Do zirconia single-retainer resin-bonded fixed dental prostheses present a viable treatment option for the replacement of missing anterior teeth? A systematic review and meta-analysis. J Prosthet Dent 2023;130(4):533-542. https://doi.org/ 10.1016/j.prosdent.2021.10.015.

[3] Al-Dabbagh RA, Sindi MA, Sanari MA, Manna AI, Al-Dabbagh MA. Effect of a circumferential ferrule on the survival and success of endodontically treated teeth restored with fiber posts: A systematic review and meta-analysis. J Prosthet Dent. 2024 Dec;132(6):1251-1259. doi: 10.1016/j.prosdent.2023.12.002.

[4] Alghauli MA, Al-Gabri RS, Keshk A, Mirah MA, Alqutaibi AY. Clinical Complications and Survival Rate of Resin Matrix Ceramic Restorations: A Systematic Review and Meta-Analysis. J Esthet Restor Dent. 2025 Jul;37(7):1721-1739. doi: 10.1111/jerd.13471.

[5] Alqutaibi AY, Alghauli MA, Almuzaini SA, Alharbi AF, Alsani AA, Mubarak AM, Alhajj MN. Failure and complication rates of different materials, designs, and bonding techniques of ceramic cantilever resin-bonded fixed dental prostheses for restoring missing anterior teeth: A systematic review and meta-analysis. J Esthet Restor Dent. 2024 Oct;36(10):1396-1411. doi: 10.1111/jerd.13238.

[6] Alqutaibi AY, Saker S, Alghauli MA, Algabri RS, AbdElaziz MH. Clinical survival and complication rate of ceramic veneers bonded to different substrates: A systematic review and meta-analysis. J Prosthet Dent. 2025 Oct;134(4):1030-1039. doi: 10.1016/j.prosdent.2024.03.019.

[7] AlSaleh E, Dutta A, Dummer PMH, Farnell DJJ, Vianna ME. Influence of remaining axial walls on of root filled teeth restored with a single crown and adhesively bonded fibre post: A systematic review and meta-analysis. J Dent 2021; 114:103813. https://doi.org/ 10.1016/j.jdent.2021.103813.

[8] Aswal GS, Rawat R, Dwivedi D, Prabhakar N, Kumar V. Clinical Outcomes of CAD/CAM (Lithium disilicate and Zirconia) Based and Conventional Full Crowns and Fixed Partial Dentures: A Systematic Review and Meta-Analysis. Cureus 2023 20;15(4):e37888. https://doi.org/ 10.7759/cureus.37888.

[9] Aykor A, Ozel E. Five-year clinical evaluation of 300 teeth restored with porcelain laminate veneers using total-etch and a modified self-etch adhesive system. Oper Dent 2009;34(5):516-23. https://doi.org/ 10.2341/08-038-C.

[10] Bagherian A, Sarraf Shirazi A, Sadeghi R. Adhesive systems under fissure sealants: yes or no?: A systematic review and meta-analysis. J Am Dent Assoc 2016;147(6):446-56 https://doi.org/ 10.1016/j.adaj.2016.01.014.

[11] Banh W, Hughes J, Sia A, Chien DCH, Tadakamadla SK, Figueredo CM, Ahmed KE. Longevity of Polymer-Infiltrated Ceramic Network and Zirconia-Reinforced Lithium Silicate Restorations: A Systematic Review and Meta-Analysis. Materials (Basel) 20213;14(17):5058. https://doi.org/ 10.3390/ma14175058.

[12] Batista VES, Bitencourt SB, Bastos NA, Pellizzer EP, Goiato MC, Dos Santos DM. Influence of the ferrule effect on the failure of fiber-reinforced composite post-and-core restorations: A systematic review and meta-analysis. J Prosthet Dent 2020;123(2):239-245. https://doi.org/ 10.1016/j.prosdent.2019.01.004.

[13] Bezerra IM, Brito ACM, de Sousa SA, Santiago BM, Cavalcanti YW, de Almeida LFD. Glass ionomer cements compared with composite resin in restoration of noncarious cervical lesions: A systematic review and meta-analysis. Heliyon. 2020 May 21;6(5):e03969. doi: 10.1016/j.heliyon.2020.e03969.

[14] Bustamante-Hernández N, Montiel-Company JM, Bellot-Arcís C, Mañes-Ferrer JF, Solá-Ruíz MF, Agustín-Panadero R, Fernández-Estevan L. Clinical Behavior of Ceramic, Hybrid and Composite Onlays. A Systematic Review and Meta-Analysis. Int J Environ Res Public Health. 2020 Oct 19;17(20):7582. doi: 10.3390/ijerph17207582.

[15] Cavalheiro CP, Scherer H, Imparato JCP, Collares FM, Lenzi TL. Use of flowable resin composite as an intermediate layer in class II restorations: a systematic review and meta-analysis. Clin Oral Investig. 2021 Oct;25(10):5629-5639. doi: 10.1007/s00784-021-04090-5.

[16] Chen J, Cai H, Ren X, Suo L, Pei X, Wan Q. A Systematic Review of the Survival and Complication Rates of All-Ceramic Resin-Bonded Fixed Dental Prostheses. J Prosthodont. 2018 Jul;27(6):535-543. doi: 10.1111/jopr.12678.

[17] de Amorim RG, Frencken JE, Raggio DP, Chen X, Hu X, Leal SC. Survival percentages of atraumatic restorative treatment (ART) restorations and sealants in posterior teeth: an updated systematic review and meta-analysis. Clin Oral Investig 2018;22(8):2703-2725. https://doi.org/10.1007/s00784-018-2625-5.

[18] de Carvalho IF, Santos Marques TM, Araújo FM, Azevedo LF, Donato H, Correia A. Clinical Performance of CAD/CAM Tooth-Supported Ceramic Restorations: A Systematic Review. Int J Periodontics Restorative Dent 2018;38(4):e68-e78. https://doi.org/10.11607/prd.3519.

[19] de Mello LS de, Galdino FF, Brooks JS, Fidalgo TK da S, Reis KR. Clinical Longevity of Sonicated and Unsonicated Composite Resin Restorations in Posterior Permanent Teeth: A Systematic Review and Meta-Analysis. Pesqui Bras Odontopediatria Clín Integr [Internet]. 2024;24:e230120. Available from: https://doi.org/10.1590/pboci.2024.083

[20] de Oliveira RP, Baia J, da Silva T, Magno MB, Maia LC, Loretto SC, da Silva E Souza MH. Does Addition of 10-MDP Monomer in Self-etch Adhesive Systems Improve the Clinical Performance of Noncarious Cervical Lesion Restorations? A Systematic Review and Meta-analysis. Oper Dent. 2021 Sep 1;46(5):E224-E239. doi: 10.2341/20-053-LIT.

[21] Dos Santos MH, Santos LR dos, Alves LNS, Moura HS, Carvalho MMSG de, Lins RBE. Do Bulk-Fill Resin Composites Present More Susceptibility to Marginal Degradation in Different Clinical Scenarios? A Systematic Review and Meta-Analysis. Pesqui Bras Odontopediatria Clín Integr [Internet]. 2022;22:e210145. Available from: https://doi.org/10.1590/pboci.2022.055

[22] Fan J, Xu Y, Si L, Li X, Fu B, Hannig M. Long-term Clinical Performance of Composite Resin or Ceramic Inlays, Onlays, and Overlays: A Systematic Review and Meta-analysis. Oper Dent 2021;46(1):25-44. https://doi.org/ 10.2341/19-107-LIT.

[23] Fathy H, Hamama HH, El-Wassefy N, Mahmoud SH. Clinical performance of resin-matrix ceramic partial coverage restorations: a systematic review. Clin Oral Investig 2022;26(5):3807-3822. https://doi.org/ 10.1007/s00784-022-04449-2.

[24] Ferrairo BM, de Azevedo-Silva LJ, Minim PR, Monteiro-Sousa RS, Pereira LF, Bitencourt SB, Cesar PF, Sidhu SK, Borges AFS. Biomechanical consideration in tooth-supported glass-ceramic restorations: A systematic review and meta-analysis of survival rates and irreparable failures. J Prosthet Dent. 2024 Nov;132(5):879.e1-879.e13. doi: 10.1016/j.prosdent.2024.05.007.

[25] Figueiredo FE, Martins-Filho PR, Faria-E-Silva AL. Do metal post-retained restorations result in more root fractures than fiber post-retained restorations? A systematic review and meta-analysis. J Endod 2015;41(3):309-16. https://doi.org/ 10.1016/j.joen.2014.10.006.

[26] Frencken JE, Liang S, Zhang Q. Survival estimates of atraumatic restorative treatment versus traditional restorative treatment: a systematic review with meta-analyses. Br Dent J 2021. https://doi.org/ 10.1038/s41415-021-2701-0.

[27] Garbim et al., 2021. Garbim JR, Laux CM, Tedesco TK, Braga MM, Raggio DP. Atraumatic restorative treatment restorations performed in different settings: systematic review and meta-analysis. Aust Dent J 2021;66(4):430-443. https://doi.org/ 10.1111/adj.12871.

[28] Gonzaga CC, Garcia PP, Wambier LM, Prochnow FHO, Madeira L, Cesar PF. Do tooth-supported zirconia restorations present more technical failures related to fracture or loss of

retention? Systematic review and meta-analysis. Clin Oral Investig 2022;26(8):5129-5142. doi: 10.1007/s00784-022-04573-z.

[29]Heintze SD, Ruffieux C, Rousson V. Clinical performance of cervical restorations--a meta-analysis. Dent Mater. 2010 Oct;26(10):993-1000. doi: 10.1016/j.dental.2010.06.003.

[30]Hong N, Yang H, Li J, Wu S, Li Y. Effect of Preparation Designs on the Prognosis of Porcelain Laminate Veneers: A Systematic Review and Meta-Analysis. Oper Dent 2017;42(6):E197-E213. https://doi.org/ 10.2341/16-390-L.

[31]Huang X, Zhang R, Yu X, Sun W, Zhang L, Hua F, Chen Z. The clinical performance of high-viscosity glass ionomer-based and bulk-fill resin-based restorations in permanent teeth with occlusal or proximal cavities: a systematic review and meta-analysis. Clin Oral Investig. 2025 Jan 7;29(1):50. doi: 10.1007/s00784-024-06127-x.

[32]Husain N, Özcan M, Molinero-Mourelle P, Joda T. Clinical Performance of Partial and Full-Coverage Fixed Dental Restorations Fabricated from Hybrid Polymer and Ceramic CAD/CAM Materials: A Systematic Review and Meta-Analysis. J Clin Med. 2020 Jul 4;9(7):2107. doi: 10.3390/jcm9072107.

[33]Kamposiora P, Papathanasiou I, Barmak AB, Chochlidakis K. EPA Consensus Project Paper: Failure Rates of Direct Versus Indirect Single-Tooth Restorations. A Systematic Review and Meta-Analysis. Eur J Prosthodont Restor Dent 2023. https://doi.org/ 10.1922/EJPRD_2489Komposiora22.

[34]Karrabi et al., 2020. Evaluate the Difference in Failure Rates between Amalgam and Composite Resin Posterior Restorations: A Systematic Review and Meta-Analysis. International Journal of Pharmaceutical Research 2020;2(1):573-579.https://doi.org/10.31838/ijpr/2020.12.01.123

[35]Klein P, Spitznagel FA, Zembic A, Prott LS, Pieralli S, Bongaerts B, Metzendorf MI, Langner R, Gierthmuehlen PC. Survival and Complication Rates of Feldspathic, Leucite-Reinforced, Lithium Disilicate and Zirconia Ceramic Laminate Veneers: A Systematic Review and Meta-Analysis. J Esthet Restor Dent. 2025 Mar;37(3):601-619. doi: 10.1111/jerd.13351.

[36]Kölüş T, Uçar AY. A Systematic Review and Meta-Analysis of the Success of Resin Composite Restorations. Journal of Advanced Oral Research. 2022;13(2):157-166. doi:10.1177/23202068221114979

[37]Kühnisch J, Bedir A, Lo YF, Kessler A, Lang T, Mansmann U, Heinrich-Weltzien R, Hickel R. Meta-analysis of the longevity of commonly used pit and fissure sealant materials. Dent Mater. 2020 May;36(5):e158-e168. doi: 10.1016/j.dental.2020.02.001.

[38]Leitão CIMB, Fernandes GVO, Azevedo LPP, Araújo FM, Donato H, Correia ARM. Clinical performance of monolithic CAD/CAM tooth-supported zirconia restorations: systematic

review and meta-analysis. J Prosthodont Res. 2022 Jul 30;66(3):374-384. doi: 10.2186/jpr.JPR_D_21_00081.

[39]Lemos CAA, Verri FR, de Luna Gomes JM, Santiago Junior JF, Miyashita E, Mendonça G, Pellizzer EP. Survival and prosthetic complications of monolithic ceramic implant-supported single crowns and fixed partial dentures: A systematic review with meta-analysis. J Prosthet Dent. 2024 Dec;132(6):1237-1249. doi: 10.1016/j.prosdent.2022.11.013.

[40]Liu Y, Hou X, Zhang W, Zhao J. Efficacy of indirect and direct resin composite restorations in permanent posterior teeth: a meta-analysis. Chin J Evid Based Med. 2017;17(6):685-691. doi:10.7507/1672-2531.201702022.

[41]Liu M, Gai K, Chen J, Jiang L. Comparison of Failure and Complication Risks of Porcelain Laminate and Indirect Resin Veneer Restorations: A Meta-Analysis. Int J Prosthodont 2019 Jan/Feb;32(1):59-65. https://doi.org/ 10.11607/ijp.6099.

[42]Manisha J, Srivastava G, Das SS, Tabarak N, Choudhury GK. Accuracy of single-unit ceramic crown fabrication after digital versus conventional impressions: A systematic review and meta-analysis. J Indian Prosthodont Soc. 2023 Apr-Jun;23(2):105-111. doi: 10.4103/jips.jips_534_22.

[43]Martins MD, Junqueira RB, de Carvalho RF, Lacerda MFLS, Faé DS, Lemos CAA. Is a fiber post better than a metal post for the restoration of endodontically treated teeth? A systematic review and meta-analysis J Dent 2021 Sep;112:103750. https:// https://doi.org/ 10.1016/j.jdent.2021.103750.

[44]Mazza LC, Lemos CAA, Pesqueira AA, Pellizzer EP. Survival and complications of monolithic ceramic for tooth-supported fixed dental prostheses: A systematic review and meta-analysis. J Prosthet Dent 2022 Oct;128(4):566-574. https:// doi: 10.1016/j.prosdent.2021.01.020.

[45]Melo GS, Batistella EÂ, Bertazzo-Silveira E, Simek Vega Gonçalves TM, Mendes de Souza BD, Porporatti AL, Flores-Mir C, De Luca Canto G. Association of sleep bruxism with ceramic restoration failure: A systematic review and meta-analysis. J Prosthet Dent. 2018 Mar;119(3):354-362. doi: 10.1016/j.prosdent.2017.07.005.

[46]Mickenautsch S, Yengopal V. Caries-preventive effect of glass ionomer and resin-based fissure sealants on permanent teeth: An update of systematic review evidence. BMC Res Notes. 2011 Jan 28;4:22. doi: 10.1186/1756-0500-4-22.

[47]Mickenautsch S. High-viscosity glass-ionomer cements for direct posterior tooth restorations in permanent teeth: The evidence in brief. J Dent. 2016 Dec;55:121-123. doi: 10.1016/j.jdent.2016.10.007.

[48] Monsarrat P, Garnier S, Vergnes JN, Nasr K, Grosgogeat B, Joniot S. Survival of directly placed ormocer-based restorative materials: A systematic review and meta-analysis of clinical trials. Dent Mater. 2017;33(5):e212-e220. doi: 10.1016/j.dental.2017.01.019.

[49] Moraschini V, Fai CK, Alto RM, Dos Santos GO. Amalgam and resin composite longevity of posterior restorations: A systematic review and meta-analysis. J Dent 2015;43(9):1043-1050. doi: 10.1016/j.jdent.2015.06.005.

[50] Morimoto S, Albanesi RB, Sesma N, Agra CM, Braga MM. Main Clinical Outcomes of Feldspathic Porcelain and Glass-Ceramic Laminate Veneers: A Systematic Review and Meta-Analysis of Survival and Complication Rates. Int J Prosthodont 2016;29(1):38-49. https://doi.org/ 10.11607/ijp.4315.

[51] Morimoto S, Rebello de Sampaio FB, Braga MM, Sesma N, Özcan M. Survival Rate of Resin and Ceramic Inlays, Onlays, and Overlays: A Systematic Review and Meta-analysis. J Dent Res 2016;95(9):985-94. https://doi.org/ 10.1177/0022034516652848.

[52] Muller-Bolla M, Lupi-Pégurier L, Tardieu C, Velly AM, Antomarchi C. Retention of resin-based pit and fissure sealants: A systematic review. Community Dent Oral Epidemiol 2006;34(5):321-36. https://doi.org/ 10.1111/j.1600-0528.2006.00319.x.

[53] Nagarkar SR, Perdigão J, Seong WJ, Theis-Mahon N. Digital versus conventional impressions for full-coverage restorations: A systematic review and meta-analysis. J Am Dent Assoc 2018 Feb;149(2):139-147.e1. https://doi.org/ 10.1016/j.adaj.2017.10.001.

[54] Naik VB, Jain AK, Rao RD, Naik BD. Comparative evaluation of clinical performance of ceramic and resin inlays, onlays, and overlays: A systematic review and meta analysis. J Conserv Dent 2022 Jul-Aug;25(4):347-355. https://doi.org/ 10.4103/jcd.jcd_184_22.

[55] Nogueira IO, Pereira CNB, Abreu LG, Diniz IMA, Magalhães CS, Silveira RRD. Do different protocols affect the success rate or bond strength of glass-ceramics repaired with composite resin? A systematic review and meta-analysis. J Prosthet Dent. 2025 May;133(5):1157-1171. doi: 10.1016/j.prosdent.2023.06.020.

[56] Pachiou A, Zervou E, Tsirogiannis P, Sykaras N. Evaluation of the marginal fit of lithium disilicate single crowns fabricated with the conventional (Emax press) and the CAD/CAM (Emax CAD) method: A systematic review and meta-analysis. Int J Comput Dent 2023;0(0):0. https://doi.org/ 10.3290/j.ijcd.b4224643.

[57] Paula AM, Boing TF, Wambier LM, Hanzen TA, Loguercio AD, Armas-Vega A, Reis A. Clinical Performance of Non-Carious Cervical Restorations Restored with the "Sandwich Technique" and Composite Resin: A Systematic Review and Meta-analysis. J Adhes Dent. 2019;21(6):497-508. doi: 10.3290/j.jad.a43696.

[58] Pauletto P, Ruales-Carrera E, Sorrentino R, Eyűboğlu TF, Özcan M. Influence of Cementation Strategies on the Incidence of Fiber Post Debonding in Root Canal Treated Teeth: A Systematic Review and Meta-Analysis. Eur J Prosthodont Restor Dent. 2024 Oct 23. doi: 10.1922/EJPRD_2679Pauletto15.

[59] Petridis HP, Zekeridou A, Malliari M, Tortopidis D, Koidis P. Survival of ceramic veneers made of different materials after a minimum follow-up period of five years: a systematic review and meta-analysis. Eur J Esthet Dent. 2012 Summer;7(2):138-52.

[60] Peumans M, De Munck J, Mine A, Van Meerbeek B. Clinical effectiveness of contemporary adhesives for the restoration of non-carious cervical lesions. A systematic review. Dent Mater 2014;30(10):1089-103. https://doi.org/ 10.1016/j.dental.2014.07.007.

[61] Peumans M, Kanumilli P, De Munck J, Van Landuyt K, Lambrechts P, Van Meerbeek B. Clinical effectiveness of contemporary adhesives: a systematic review of current clinical trials. Dent Mater. 2005 Sep;21(9):864-81. doi: 10.1016/j.dental.2005.02.003.

[62] Pilcher L, Pahlke S, Urquhart O, O'Brien KK, Dhar V, Fontana M, González-Cabezas C, Keels MA, Mascarenhas AK, Nascimento MM, Platt JA, Sabino GJ, Slayton RL, Tinanoff N, Young DA, Zero DT, Tampi MP, Purnell D, Salazar J, Megremis S, Bienek D, Carrasco-Labra A. Direct materials for restoring caries lesions: Systematic review and meta-analysis-a report of the American Dental Association Council on Scientific Affairs. J Am Dent Assoc 2023;154(2):e1-e98. https://doi.org/ 10.1016/j.adaj.2022.09.012. Erratum in: J Am Dent Assoc. 2023;154(4):A9. https://doi.org/ 10.1016/j.adaj.2023.02.002.

[63] Pjetursson BE, Sailer I, Makarov NA, Zwahlen M, Thoma DS. All-ceramic or metal-ceramic tooth-supported fixed dental prostheses (FDPs)? A systematic review of the survival and complication rates. Part II: Multiple-unit FDPs. Dent Mater. 2015 Jun;31(6):624-39. doi: 10.1016/j.dental.2015.02.013. Epub 2015 Apr 30. Erratum in: Dent Mater. 2017 Jan;33(1):e48-e51. doi: 10.1016/j.dental.2016.09.033.

[64] Pjetursson BE, Valente NA, Strasding M, Zwahlen M, Liu S, Sailer I. A systematic review of the survival and complication rates of zirconia-ceramic and metal-ceramic single crowns. Clin Oral Implants Res. 2018 Oct;29 Suppl 16:199-214. doi: 10.1111/clr.13306. PMID: 30328190.

[65] Qin W, Lei L, Huang QT, Wang L, Lin ZM. Clinical effectiveness of self-etching adhesives with or without selective enamel etching in noncarious cervical lesions: A systematic review. J Dent Sci. 2014 Dec;9(4):303-312. doi: 10.1016/j.jds.2014.03.002.

[66] Qiu J, Liu W, Wu D, Qiao F, Sui L. Fit accuracy in the rest region of RPDs fabricated by digital technologies and conventional lost-wax casting: a systematic review and meta-analysis. BMC Oral Health. 2023 Sep 15;23(1):667. doi: 10.1186/s12903-023-03348-6.

[67]Rodrigues SB, Franken P, Celeste RK, Leitune VCB, Collares FM. CAD/CAM or conventional ceramic materials restorations longevity: a systematic review and meta-analysis. J Prosthodont Res 2019;63(4):389-395. https://doi.org/ 10.1016/j.jpor.2018.11.006.

[68]Saini RS, Vyas R, Vaddamanu SK, Quadri SA, Mosaddad SA, Heboyan A. Efficacy of different adhesive systems in bonding direct resin composite restorations: a systematic review and meta-analysis. Evid Based Dent. 2025. https://doi.org/10.1038/s41432-024-01095-3.

[69]Sailer I, Makarov NA, Thoma DS, Zwahlen M, Pjetursson BE. All-ceramic or metal-ceramic tooth-supported fixed dental prostheses (FDPs)? A systematic review of the survival and complication rates. Part I: Single crowns (SCs). Dent Mater. 2015 Jun;31(6):603-23. doi: 10.1016/j.dental.2015.02.011. Epub 2015 Apr 2. Erratum in: Dent Mater. 2016 Dec;32(12):e389-e390. doi: 10.1016/j.dental.2016.09.032.

[70]Saravi B, Vollmer A, Hartmann M, Lang G, Kohal RJ, Boeker M, Patzelt SBM. Clinical Performance of CAD/CAM All-Ceramic Tooth-Supported Fixed Dental Prostheses: A Systematic Review and Meta-Analysis. Materials (Basel). 2021 May 20;14(10):2672. doi: 10.3390/ma14102672. PMID: 34065202; PMCID: PMC8161295.

[71]Sampaio FBWR, Özcan M, Gimenez TC, Moreira MSNA, Tedesco TK, Morimoto S. Effects of manufacturing methods on the survival rate of ceramic and indirect composite restorations: A systematic review and meta-analysis. J Esthet Restor Dent 2019;31(6):561-571. https://doi.org/ 10.1111/jerd.12555.

[72]Schwendicke F, Jäger AM, Paris S, Hsu LY, Tu YK. Treating pit-and-fissure caries: a systematic review and network meta-analysis. J Dent Res 2015;94(4):522-33. https://doi.org/ 10.1177/0022034515571184.

[73]Shaalan OO, Abou-Auf E, El Zoghby AF. Clinical evaluation of flowable resin composite versus conventional resin composite in carious and noncarious lesions: Systematic review and meta-analysis. J Conserv Dent. 2017 Nov-Dec;20(6):380-385. doi: 10.4103/JCD.JCD_226_17.

[74]Shah M, Patel MC, Patel F, Fernandes M, Makwani D, Mehta M. Evaluation of Glass Ionomer Cement and Composite Resin Restorations in Hypomineralized Permanent First Molars: A Systematic Review and Meta-Analysis. Cureus. 2025 Mar 27;17(3):e81265. doi: 10.7759/cureus.81265.

[75]Shenoy VK, Bangera MK, Miranda G, Rodrigues A, Shenoy RK, Mehendale A. Influence of presence or absence of posts on the failure rates of post endodontic restorations: A systematic review and meta-analysis. J Indian Prosthodont Soc 2022;22(2):122-130. https://doi.org/ 10.4103/jips.jips_315_21.

[76]Shihabi S, Chrcanovic BR. Clinical outcomes of tooth-supported monolithic zirconia vs. porcelain-veneered zirconia fixed dental prosthesis, with an additional focus on the cement

type: a systematic review and meta-analysis. Clin Oral Investig 2023;27(10):5755-5769. https://doi.org/ 10.1007/s00784-023-05219-4.

[77]Shu X, Mai QQ, Blatz M, Price R, Wang XD, Zhao K. Direct and Indirect Restorations for Endodontically Treated Teeth: A Systematic Review and Meta-analysis, IAAD 2017 Consensus Conference Paper. J Adhes Dent 2018;20(3):183-194. https://doi.org/ 10.3290/j.jad.a40762.

[78]Skupien JA, Luz MS, Pereira-Cenci T. Ferrule Effect: A Meta-analysis. JDR Clin Trans Res 2016;1(1):31-39. https://doi.org/ 10.1177/2380084416636606.

[79]Tabesh M, Nejatidanesh F, Savabi G, Davoudi A, Savabi O, Mirmohammadi H. Marginal adaptation of zirconia complete-coverage fixed dental restorations made from digital scans or conventional impressions: A systematic review and meta-analysis. J Prosthet Dent 2021 Apr;125(4):603-610. https://doi.org/ 10.1016/j.prosdent.2020.01.035.

[80]Thammajaruk P, Guazzato M, Naorungroj S. Cleaning methods of contaminated zirconia: A systematic review and meta-analysis. Dent Mater. 2023 Mar;39(3):235-245. doi: 10.1016/j.dental.2023.01.009.

[81]Vagropoulou GI, Klifopoulou GL, Vlahou SG, Hirayama H, Michalakis K. Complications and survival rates of inlays and onlays vs complete coverage restorations: A systematic review and analysis of studies. J Oral Rehabil 2018 Nov;45(11):903-920. https://doi.org/ 10.1111/joor.12695.

[82]van de Sande FH, Collares K, Correa MB, Cenci MS, Demarco FF, Opdam N. Restoration Survival: Revisiting Patients' Risk Factors Through a Systematic Literature Review. Oper Dent 2016 Sep;41(S7):S7-S26. https://doi.org/ 10.2341/15-120-LIT.

[83]Vieira BR, Dantas ELA, Cavalcanti YW, Santiago BM, Sousa FB. Comparison of Self-Etching Adhesives and Etch-and-Rinse Adhesives on the Failure Rate of Posterior Composite Resin Restorations: A Systematic Review and Meta-Analysis. Eur J Dent. 2022 May;16(2):258-265. doi: 10.1055/s-0041-1736332.

[84]Zou Y, Bai J, Xiang Z. Survival rate of ceramic inlay and onlay restorations in posterior teeth with one-surface or multi-surface after a 10-year follow-up: A systematic review and meta-analysis. Vojnosanit Pregl 2021; 78(5): 549–555. https://doi.org/10.2298/VSP161208034Z.

[85]Yuen JJX, Saw ZK, Chua HS, Beh YH. Clinical behavior and survival of CAD-CAM resin nanoceramic and polymer interpenetrating ceramic network material restorations on endodontically treated teeth: A systematic review and meta-analysis. J Prosthet Dent. 2025:S0022-3913(25)00052-6. https://doi.org/10.1016/j.prosdent.2025.01.019.

[86]Wen Y, Zhao X, Li SKY, Lo ECM, Jiang CM. Factors associated with the success of restorative treatment for root caries: A systematic review with meta-analysis. J Dent. 2025 Feb;153:105539. https://doi.org/10.1016/j.jdent.2024.105539.

[87]Wittich FK, Cebula M, Effenberger S, Schoppmeier CM, Schwendicke F, Barbe AG, Wicht MJ. Masking efficacy of bleaching and/ or resin infiltration of fluorotic spots on anterior teeth - a systematic review and meta-analysis. J Dent. 2024 Oct;149:105276. doi: 10.1016/j.jdent.2024.105276.

[88]Yuen JJX, Saw ZK, Chua HS, Beh YH. Clinical behavior and survival of CAD-CAM resin nanoceramic and polymer interpenetrating ceramic network material restorations on endodontically treated teeth: A systematic review and meta-analysis. J Prosthet Dent. 2025 Jun;133(6):1437.e1-1437.e9. doi: 10.1016/j.prosdent.2025.01.019.

[89]Zou Y, Bai J, Xiang JZ. Survival rate of ceramic inlay and onlay resto-rations in posterior teeth with one-surface or multi-surface after a10-year follow-up: a systematic review and meta-analysis. VojnosanitPregl. 2021;78(5):549-555.

Lacking Treatment/Material Comparison (n=17)

[1] Beck F, Lettner S, Graf A, Bitriol B, Dumitrescu N, Bauer P, Moritz A, Schedle A. Survival of direct resin restorations in posterior teeth within a 19-year period (1996-2015): A meta-analysis of prospective studies. Dent Mater. 2015;31(8):958-85. https://doi.org/10.1016/j.dental.2015.05.004.

[2] Bezerra IM, Brito ACM, de Sousa SA, Santiago BM, Cavalcanti YW, de Almeida LFD. Glass ionomer cements compared with composite resin in restoration of noncarious cervical lesions: A systematic review and meta-analysis. Heliyon 2020;6(5):e03969. https://doi.org/10.1016/j.heliyon.2020.e03969.

[3] Bustamante-Hernández N, Montiel-Company JM, Bellot-Arcís C, Mañes-Ferrer JF, Solá-Ruíz MF, Agustín-Panadero R, Fernández-Estevan L. Clinical Behavior of Ceramic, Hybrid and Composite Onlays. A Systematic Review and Meta-Analysis. Int J Environ Res Public Health. 2020;17(20):7582. https://doi.org/10.3390/ijerph17207582.

[4] Fathy H, Hamama HH, El-Wassefy N, Mahmoud SH. Clinical performance of resin-matrix ceramic partial coverage restorations: a systematic review. Clin Oral Investig. 2022 May;26(5):3807-3822. doi: 10.1007/s00784-022-04449-2.

[5] Jokstad A. After 10 years seven out of ten fixed dental prostheses (FDP) remain intact and nine out of ten FDPs remain in function following biological and technical complications that have been repaired. J Evid Based Dent Pract. 2010 Mar;10(1):39-40. doi: 10.1016/j.jebdp.2009.11.019.

[6] Jung RE, Pjetursson BE, Glauser R, Zembic A, Zwahlen M, Lang NP. A systematic review of the 5-year survival and complication rates of implant-supported single crowns. Clin Oral Implants Res. 2008 Feb;19(2):119-30. doi: 10.1111/j.1600-0501.2007.01453.x. Epub 2007 Dec 7. PMID: 18067597.

[7] Kamposiora P, Papathanasiou I, Barmak AB, Chochlidakis K. EPA Consensus Project Paper: Failure Rates of Direct Versus Indirect Single-Tooth Restorations. A Systematic Review and Meta-Analysis. Eur J Prosthodont Restor Dent. 2023 May 5. doi: 10.1922/EJPRD_2489Komposiora22. Epub ahead of print. PMID: 37191573.

[8] Lampl S, Gurunathan D, Krithikadatta J, Mehta D, Moodley D. Reasons for Failure of CAD/CAM Restorations in Clinical Studies: A Systematic Review and Meta-analysis. J Contemp Dent Pract. 2023;24(2):129-136. https://doi.org/10.5005/jp-journals-10024-3472.

[9] Layton DM, Clarke M. A systematic review and meta-analysis of the survival of non-feldspathic porcelain veneers over 5 and 10 years. Int J Prosthodont 2013 Mar-Apr;26(2):111-24. https://doi.org/ 10.11607/ijp.3202.

[10] Layton DM, Clarke M, Walton TR. A systematic review and meta-analysis of the survival of feldspathic porcelain veneers over 5 and 10 years. Int J Prosthodont 2012;25(6):590-603.

[11] Leitão CIMB, Fernandes GVO, Azevedo LPP, Araújo FM, Donato H, Correia ARM. Clinical performance of monolithic CAD/CAM tooth-supported zirconia restorations: systematic review and meta-analysis. J Prosthodont Res 2022;66(3):374-384. https://doi.org/ 10.2186/jpr.JPR_D_21_00081.

[12] Lemos CAA, Verri FR, de Luna Gomes JM, Santiago Junior JF, Miyashita E, Mendonça G, Pellizzer EP. Survival and prosthetic complications of monolithic ceramic implant-supported single crowns and fixed partial dentures: A systematic review with meta-analysis. J Prosthet Dent 2022:S0022-3913(22)00736-3. https://doi.org/ 10.1016/j.prosdent.2022.11.013.

[13] Mungur A, Chen H, Shahid S, Baysan A. A systematic review on the effect of silver diamine fluoride for management of dental caries in permanent teeth. Clin Exp Dent Res. 2023 Apr;9(2):375-387. doi: 10.1002/cre2.716.

[14] Pjetursson BE, Tan WC, Tan K, Brägger U, Zwahlen M, Lang NP. A systematic review of the survival and complication rates of resin-bonded bridges after an observation period of at least 5 years. Clin Oral Implants Res. 2008 Feb;19(2):131-41. doi: 10.1111/j.1600-0501.2007.01527.x. Epub 2007 Dec 7.

[15] Opdam NJ, van de Sande FH, Bronkhorst E, Cenci MS, Bottenberg P, Pallesen U, Gaengler P, Lindberg A, Huysmans MC, van Dijken JW. Longevity of posterior composite restorations: a systematic review and meta-analysis. J Dent Res 2014;93(10):943-9. https://doi.org/ 10.1177/0022034514544217.

[16] van Heumen CC, Kreulen CM, Creugers NH. Clinical studies of fiber-reinforced resin-bonded fixed partial dentures: a systematic review. Eur J Oral Sci. 2009 Feb;117(1):1-6. doi: 10.1111/j.1600-0722.2008.00595.x.

17] Zhang X, Pei X, Pei X, Wan Q, Chen J, Wang J. Success and Complication Rates of Root-Filled Teeth Restored with Zirconia Posts: A Critical Review. Int J Prosthodont 2019;32(5):411-419. https://doi.org/ 10.11607/ijp.6179.

Did not include meta-analysis of the outcomes of interest (n= 31):

[1] Askar H, Krois J, Göstemeyer G, Schwendicke F. Secondary caries risk of different adhesive strategies and restorative materials in permanent teeth: Systematic review and network meta-analysis. J Dent 2021;104:103541. https://doi.org/10.1016/j.jdent.2020.103541.

[2] Cidreira Boaro LC, Pereira Lopes D, de Souza ASC, Lie Nakano E, Ayala Perez MD, Pfeifer CS, Gonçalves F. Clinical performance and chemical-physical properties of bulk fill composites resin - a systematic review and meta-analysis. Dent Mater. 2019 Oct;35(10):e249-e264. doi: 10.1016/j.dental.2019.07.007. Epub 2019 Aug 14. PMID: 31421957.

[3] Correia A, Bresciani E, Borges AB, Pereira DM, Maia LC, Caneppele T. Do Tooth- and Cavity-related Aspects of Noncarious Cervical Lesions Affect the Retention of Resin Composite Restorations in Adults? A Systematic Review and Meta-analysis. Oper Dent 2020;45(3):E124-E140. https://doi.org/10.2341/19-091-L.

[4] Cribari L, Madeira L, Roeder RBR, Macedo RM, Wambier LM, Porto TS, Gonzaga CC, Kaizer MR. High-viscosity glass-ionomer cement or composite resin for restorations in posterior permanent teeth? A systematic review and meta-analyses. J Dent 2023;137:104629. https://doi.org/10.1016/j.jdent.2023.104629.

[5] Dorri M, Dunne SM, Walsh T, Schwendicke F. Micro-invasive interventions for managing proximal dental decay in primary and permanent teeth. Cochrane Database Syst Rev 2015;2015(11):CD010431. https://doi.org/10.1002/14651858.CD010431.pub2.

[6] dos Santos MH, Santos LR dos, Alves LNS, Moura HS, Carvalho MMSG de, Lins RBE. Do Bulk-Fill Resin Composites Present More Susceptibility to Marginal Degradation in Different Clinical Scenarios? A Systematic Review and Meta-Analysis. Pesqui Bras Odontopediatria Clín Integr 2022;22:e210145. https://doi.org/10.1590/pboci.2022.055

[7] Fröhlich TT, Rocha RO, Botton G. Does previous application of silver diammine fluoride influence the bond strength of glass ionomer cement and adhesive systems to dentin? Systematic review and meta-analysis. Int J Paediatr Dent. 2020 Jan;30(1):85-95. doi: 10.1111/ipd.12571. Epub 2019 Sep 18. PMID: 31419356.

[8] Garcia PP, Wambier LM, de Geus JL, da Cunha LF, Correr GM, Gonzaga CC. Do anterior and posterior teeth treated with post-and-core restorations have similar failure rates? A systematic review and meta-analysis. J Prosthet Dent 2019;121(6):887-894.e4. https://doi.org/10.1016/j.prosdent.2018.08.004.

[9] Heintze SD, Ruffieux C, Rousson V. Clinical performance of cervical restorations--a meta-analysis. Dent Mater 2010;26(10):993-1000. https://doi.org/10.1016/j.dental.2010.06.003.

[10] Heintze SD, Ruffieux C, Rousson V. Clinical performance of cervical restorations--a meta-analysis. Dent Mater 2010 Oct;26(10):993-1000. https://doi.org/10.1016/j.dental.2010.06.003.

[11] Hong X, Huang Z, Tong Z, Jiang H, Su M. Clinical effects of different etching modes for universal adhesives: a systematic review and meta-analysis. Ann Palliat Med 2021;10(5):5462-5473. https://doi.org/10.21037/apm-21-890.

[12] Jurema ALB, Filgueiras AT, Santos KA, Bresciani E, Caneppele TMF. Effect of intraradicular fiber post on the fracture resistance of endodontically treated and restored anterior teeth: A systematic review and meta-analysis. J Prosthet Dent 2022;128(1):13-24. https://doi.org/10.1016/j.prosdent.2020.12.013.

[13] Maran BM, de Geus JL, Gutiérrez MF, Heintze S, Tardem C, Barceleiro MO, Reis A, Loguercio AD. Nanofilled/nanohybrid and hybrid resin-based composite in patients with direct restorations in posterior teeth: A systematic review and meta-analysis. J Dent 2020;99:103407. https://doi.org/10.1016/j.jdent.2020.103407.

[14] de Souza Melo G, Batistella EÂ, Bertazzo-Silveira E, Simek Vega Gonçalves TM, Mendes de Souza BD, Porporatti AL, Flores-Mir C, De Luca Canto G. Association of sleep bruxism with ceramic restoration failure: A systematic review and meta-analysis. J Prosthet Dent 2018;119(3):354-362. https://doi.org/10.1016/j.prosdent.2017.07.005.

[15] Mickenautsch S, Yengopal V. Caries-preventive effect of glass ionomer and resin-based fissure sealants on permanent teeth: An update of systematic review evidence. BMC Res Notes 2011;4:22. https://doi.org/10.1186/1756-0500-4-22.

[16] Mungur A, Chen H, Shahid S, Baysan A. A systematic review on the effect of silver diamine fluoride for management of dental caries in permanent teeth. Clin Exp Dent Res 2023 Apr;9(2):375-387. https://doi.org/10.1002/cre2.716.

[17] Nedeljkovic I, Teughels W, De Munck J, Van Meerbeek B, Van Landuyt KL. Is secondary caries with composites a material-based problem? Dent Mater 2015;31(11):e247-77. https://doi.org/10.1016/j.dental.2015.09.001.

[18] Palmier NR, Madrid Troconis CC, Normando AGC, Guerra ENS, Araújo ALD, Arboleda LPA, Fonsêca JM, de Pauli Paglioni M, Gomes-Silva W, Vechiato Filho AJ, González-Arriagada WA, Paes Leme AF, Prado-Ribeiro AC, Brandão TB, de Goes MF, Lopes MA, Santos-Silva AR. Impact of head

and neck radiotherapy on the longevity of dental adhesive restorations: A systematic review and meta-analysis. J Prosthet Dent 2022;128(5):886-896. https://doi.org/10.1016/j.prosdent.2021.02.002.

[19] Pinto NS, Jorge GR, Vasconcelos J, Probst LF, De-Carli AD, Freire A. Clinical efficacy of bioactive restorative materials in controlling secondary caries: a systematic review and network meta-analysis. BMC Oral Health 2023;23(1):394. https://doi.org/10.1186/s12903-023-03110-y.

[20] Poggio CE, Ercoli C, Rispoli L, Maiorana C, Esposito M. Metal-free materials for fixed prosthodontic restorations. Cochrane Database Syst Rev 2017 Dec 20;12(12):CD009606. https://doi.org/10.1002/14651858.CD009606.pub2.

[21] Qin et al., 2014. Clinical effectiveness of self-etching adhesives with or without selective enamel etching in noncarious cervical lesions: A systematic review. Journal of Dental Sciences 2014;9(4):303-312. https://doi.org/10.1016/j.jds.2014.03.002.

[22] Ruengrungsom C, Palamara JEA, Burrow MF. Comparison of ART and conventional techniques on clinical performance of glass-ionomer cement restorations in load bearing areas of permanent and primary dentitions: A systematic review. J Dent. 2018 Nov;78:1-21. doi: 10.1016/j.jdent.2018.07.008.

[23] Saber AM, El-Housseiny AA, Alamoudi NM. Atraumatic Restorative Treatment and Interim Therapeutic Restoration: A Review of the Literature. Dent J (Basel) 2019;7(1):28. https://doi.org/10.3390/dj7010028.

[24] Shaalan OO, Abou-Auf E, El Zoghby AF. Clinical evaluation of flowable resin composite versus conventional resin composite in carious and noncarious lesions: Systematic review and meta-analysis. J Conserv Dent 2017;20(6):380-385. https://doi.org/10.4103/JCD.JCD_226_17.

[25] Sorrentino R, Di Mauro MI, Ferrari M, Leone R, Zarone F. Complications of endodontically treated teeth restored with fiber posts and single crowns or fixed dental prostheses-a systematic review. Clin Oral Investig 2016;20(7):1449-57. https://doi.org/10.1007/s00784-016-1919-8.

[26] Sousa SJL, Poubel DLDN, Rezende LVML, Almeida FT, de Toledo IP, Garcia FCP. Early clinical performance of resin cements in glass-ceramic posterior restorations in adult vital teeth: A systematic review and meta-analysis. J Prosthet Dent 2020;123(1):61-70. https://doi.org/10.1016/j.prosdent.2018.12.006.

[27]Tabesh M, Nejatidanesh F, Savabi G, Davoudi A, Savabi O, Mirmohammadi H. Marginal adaptation of zirconia complete-coverage fixed dental restorations made from digital scans or conventional impressions: A systematic review and meta-analysis. J Prosthet Dent. 2021 Apr;125(4):603-610. doi: 10.1016/j.prosdent.2020.01.035. Epub 2020 Apr 10. PMID: 32284188.

[28] Thammajaruk P, Guazzato M, Naorungroj S. Cleaning methods of contaminated zirconia: A systematic review and meta-analysis. Dent Mater 2023;39(3):235-245. https://doi.org/10.1016/j.dental.2023.01.009.

[29] Yengopal V, Mickenautsch S. Caries-preventive effect of resin-modified glass-ionomer cement (RM-GIC) versus composite resin: a quantitative systematic review. Eur Arch Paediatr Dent. 2011 Feb;12(1):5-14. https://doi.org/10.1007/BF03262772.

[30] Zhang SN, DU Q, Han L, Liu P, Li T, Zhang LL. [Fiber post versus metal screw post for repair of residual crowns and roots: a systematic review]. Shanghai Kou Qiang Yi Xue. 2014 ;23(2):237-42. Chinese. PMID: 24935852.

[31] Zotti F, Falavigna E, Capocasale G, De Santis D, Albanese M. Microleakage of Direct Restorations-Comparisonbetween Bulk-Fill and Traditional Composite Resins:Systematic Review and Meta-Analysis. Eur J Dent 2021;15(4):755-767. https://doi.org/10.1055/s-0041-1724155.

Untranslated Studies (n=1)

[1] Liu Y, Hou X, Zhang W, Zhao J. Efficacy of indirect and direct resin composite restorations in permanent posterior teeth: A meta-analysis. Chinese J Evidence-Based Med [Internet]. 2017;17(6):685–91.

Unavailable Studies (n= 4):

[1] Aziz IM, Locke M. Success and Survival of Composite Resin Restorations for the Management of Localized Anterior Tooth Wear: A Systematic Review and Meta-Analysis. Eur J Prosthodont Restor Dent. 2024;32(4):403-414. https://doi.org/10.1922/EJPRD_2576Aziz12.

[2] Boruziniat A, Gharaee S, Sarraf Shirazi A, Majidinia S, Vatanpour M. Evaluation of the efficacy of flowable composite as lining material on microleakage of composite resin restorations: A

systematic review and meta-analysis. Quintessence Int. 2016 Feb;47(2):93-101. doi: 10.3290/j.qi.a35260.

[3] Deshmukh VS, Joshi PB, Madi RS, Nirmal SS, Rane SS, Kulkarni KM. Evaluation Of Bond Strength of Indirect Composite with Titanium and Co-Cr Framework - A Systematic Review. Journal of Prosthodontic Research 2022. https://doi.org/10.1016/j.jpor.2021.09.001.

[4] Niakou A, Stratos A, Pissadakis M, Kamalakidis S, Michalakis P, Pissiotis A, Baba NZ, Tsouknidas A, Vasilaki D, Michalakis K. Complications and Survival Rates of Endocrowns vs. Crowns: A Systematic Review and Meta-Analysis. Int J Prosthodont. 2025 Oct 16:1-33. doi: 10.11607/ijp.9498. Epub ahead of print.

Primary and permanent teeth together (n= 1)

[1] Mickenautsch S, Yengopal V. Failure rate of high-viscosity GIC based ART compared with that of conventional amalgam restorations--evidence from an update of a systematic review. SADJ. 2012 Aug;67(7):329-31. PMID: 23951787.
