# Supplementary material for: Spin and reporting quality in systematic reviews of nonrandomized studies in restorative dentistry: a meta-research study
Source: Braz Oral Res. 2026 Nov 9;40:e094. doi: 10.1590/1807-3107bor-2026.vol40.094 (PMC13750809; doi:10.1590/1807-3107bor-2026.vol40.094)
Supplement: Supplementary file 4 [file 1807-3107-bor-40-e094-suppl04.pdf]

Supplementary Table 4. Variables extracted from the included systematic reviews, grouped by category.

Variable Group	Variable	Data Type	Description
1. General characteristics of the review	Number of included primary studies	Quantitative	Total number of primary studies included in the meta-analysis
	Number of reported outcomes (primary and secondary)	Quantitative	As reported or inferred
	Distinction between primary and secondary outcomes	Categorical (Yes/No)	Based on explicit declaration or first outcome reported
	Use of a risk of bias tool	Categorical (Yes/No)	Name of the tool recorded
	Use of GRADE	Categorical (Yes/No)	For certainty of evidence assessment
2. Journal-related characteristics	Open access status	Categorical (Yes/No)	Based on journal policy
	Journal impact factor	Quantitative (continuous)	From JCR at the time of publication
	Continent of corresponding author	Categorical	e.g., Europe, Americas, Asia
	Citation count in Scopus	Quantitative	Retrieved up to February 2025
3. Methodological and reporting features	Protocol registration	Categorical (Yes/No)	Registry number included when available
	Declaration of funding source	Categorical (Yes/No)	Classified as public, private, mixed, or not reported
	Declaration of conflicts of interest	Categorical (Yes/No)	As stated in the manuscript
	PRISMA adherence	Categorical (Yes/No)	Independently verified by two reviewers
	Structured abstract	Categorical	Based on journal formatting model
	Abstract word count	Quantitative	Compared with journal's permitted word limit
	Exceeded abstract word limit	Categorical (Yes/No)	Binary variable created
4. Spin-related variables	Presence of spin in the abstract	Categorical (Yes/No)	Based on criteria from Yavchitz et al. (2016)
	Level of spin in the abstract	Categorical (None/Low/High)	According to predefined classification
	Number of spin items in the abstract	Quantitative	Up to 21 possible items
	Number of spin items in the main text	Quantitative	Up to 28 possible items
