# Supplementary material for: Spin and reporting quality in systematic reviews of nonrandomized studies in restorative dentistry: a meta-research study
Source: Braz Oral Res. 2026 Nov 9;40:e094. doi: 10.1590/1807-3107bor-2026.vol40.094 (PMC13750809; doi:10.1590/1807-3107bor-2026.vol40.094)
Supplement: Supplementary file 5 [file 1807-3107-bor-40-e094-suppl05.pdf]

Supplementary Table 5. Operational definitions of spin criteria

This supplementary material provides the detailed criteria used to assess *spin* in the included systematic reviews and meta-analyses. The classification is adapted from Yavchitz et al. (2016) and Boutron et al. (2010).

Main category	Description of spin in the main text	Description of spin in abstract
Misleading reporting	**(1) Failure to acknowledge a departure from protocol that could modify the interpretation of results**<br><br>**Explanation:** judged present when deviations from the registered protocol (if available) or stated methods were not mentioned.	**(1) Selective reporting of or overemphasis on efficacy outcomes favoring the beneficial effect of the experimental intervention**<br><br>**Explanation:** judged when conclusions highlighted secondary or subgroup outcomes with positive results instead of the main outcome.
	**(2) Selective reporting of or overemphasis on efficacy outcomes favoring the beneficial effect of the experimental intervention (e.g., secondary outcomes, subgroup analyses)**<br><br>**Explanation:** identified when secondary outcomes or subgroup analyses were highlighted instead of primary outcome results.	**(2) Selective reporting of or overemphasis on harm outcomes favoring the safety of the experimental intervention**<br><br>**Explanation:** recorded when safety outcomes were emphasized despite limited or nonsignificant data on harms.
	**(3) Selective reporting of or overemphasis on harm outcomes favoring the safety of the experimental intervention**<br><br>**Explanation:** considered present when harms with favorable safety interpretation were selectively emphasized.	**(3) Failure to report a wide confidence interval of estimates**<br><br>**Explanation:** for ratio measures (e.g., OR, RR, HR), an interval was considered wide when (upper limit – lower limit) > 1.0.
	**(4) No or inadequate reporting of the limitations of the systematic review**<br><br>**Explanation:** marked when limitations regarding bias, heterogeneity, or imprecision were omitted.	**(4) Authors hide or do not present any conflict of interest**<br><br>**Explanation:** judged when the abstract did not disclose funding sources or potential conflicts of interest

Misleading reporting	(5) Selective citation of articles in favor of the beneficial effect of the experimental intervention<br><br>Explanation: assessed when citations disproportionately supported beneficial effects, ignoring contradictory evidence.	(5) Inadequate focus on the results of primary studies favoring the beneficial effect of the experimental intervention instead of the meta-analysis results<br><br>Explanation: judged when the abstract emphasized findings from individual trials showing benefit, while minimizing or omitting the pooled meta-analysis estimates.
	(6) Authors hide or do not present any conflict of interest<br><br>Explanation: judged when no statement about conflicts of interest or funding was provided.	(6) Conclusion focusing selectively on statistically significant efficacy outcome<br><br>Explanation: judged when the abstract conclusion highlighted only significant efficacy results (e.g., secondary or subgroup outcomes), while ignoring the nonsignificant primary outcome.
	(7) Conclusion focusing selectively on statistically significant efficacy outcome<br><br>Explanation: present when conclusions ignored the nonsignificant primary outcome.	(7) Failure to report the number of studies/patients actually contributing to the analysis for main outcomes<br><br>Explanation: judged when abstracts did not specify how many studies or participants were included in the analyses of the main outcomes
	(8) Selective reporting of analysis favoring the beneficial effect of the experimental intervention (e.g., selective analysis using random or fixed effect according the results)<br><br>Explanation: noted when authors chose the model post hoc according to desired results.	(8) Failure to specify the direction of the effect when it favors the control intervention<br><br>Explanation: judged when the abstract reported an effect estimate or statistical result but did not clearly state that the effect favored the control group, potentially leading readers to assume benefit of the experimental intervention
	(9) Inadequate focus on the results of primary studies favoring the beneficial effect of the experimental intervention instead of the meta-analysis results	

	**Explanation:** identified when authors emphasized individual trials more than pooled results.	
	**(10) Changing the scale of the forest plot to magnify the results (diamond size)**<br><br>**Explanation:** considered present when the figure scaling magnifies the diamond size or effect visually.	
Misleading interpretation	**(11) Title claims or suggests a beneficial effect of the experimental intervention not supported by the findings**<br><br>**Explanation:** judged present when the title wording implied efficacy of a treatment not demonstrated.	**(9) Title claims or suggests a beneficial effect of then experimental intervention not supported by the findings**<br><br>**Explanation:** judged when the title was written in a way that implied efficacy or superiority of the intervention, although the main results were nonsignificant or did not support such benefit.
	**(12) Inadequate interpretation of nonstatistically significant results (with a wide confidence interval) as a lack of effect or an equivalent effect for efficacy outcomes**<br><br>**Explanation:** considered when wide CI was ignored and lack of significance treated as equivalence. For ratio measures (e.g., OR, RR, HR), an interval was considered wide when (upper limit – lower limit) > 1.0.	**(10) Inadequate focus on P-value instead of magnitude of the effect estimates for harm or efficacy outcome**<br><br>**Explanation:** judged when the abstract emphasized only whether results were statistically significant ($p < 0.05$) without presenting or discussing the size and clinical relevance of the effect estimates (e.g., risk difference, mean difference, confidence interval).
	**(13) Inadequate interpretation of nonstatistically significant results (with a wide confidence interval) as demonstrating safety for harm outcome**<br><br>**Explanation:** identified when interpretation relied mainly on p-value without magnitude.	**(11) Focus on relative effect when the absolute effect is small**<br><br>**Explanation:** judged when the abstract highlighted relative measures of effect (e.g., risk ratio, odds ratio) that suggested a large benefit, while the corresponding absolute effect size (e.g., risk difference, number needed to treat) was small and of limited clinical relevance.

Misleading interpretation	**(14) Inadequate focus on P-value instead of magnitude of the effect estimates for harm or efficacy outcome**<br><br>**Explanation:** identified when interpretation relied mainly on p-value without magnitude.	**(12) Conclusion claiming equivalence or comparable effectiveness for nonstatistically significant results with a wide confidence interval**<br><br>**Explanation:** judged when the abstract conclusion interpreted nonsignificant results as proof of equivalence or similar effectiveness, even though the wide confidence interval included both potential benefit and harm.
	**(15) Focus on relative effect when the absolute effect is small**<br><br>**Explanation:** judged when large relative benefit but trivial absolute benefit was highlighted.	**(13) Conclusion formulating recommendations for clinical practice not supported by the findings**<br><br>**Explanation:** judged when the abstract provided recommendations for clinical use of a restorative intervention despite insufficient, nonsignificant, or low-certainty evidence from the review.
	**(16) Misleading interpretation of cited articles, favoring the beneficial effect of the experimental intervention**<br><br>**Explanation:** present when citations were distorted in favor of benefit.	**(14) Conclusion claiming safety based on nonstatistically significant results with a wide confidence interval**<br><br>**Explanation:** judged when the abstract interpreted nonsignificant results regarding harms as evidence of safety, despite a wide confidence interval that included the possibility of clinically relevant adverse effects. For ratio measures (e.g., OR, RR, HR), an interval was considered wide when (upper limit – lower limit) > 1.0.
	**(17) Conclusion claiming equivalence or comparable effectiveness for nonstatistically significant results with a wide confidence interval**<br><br>**Explanation:** same operational rule as #12, focusing on wording like “similar” or “equivalent.”	**(15) Conclusion claiming the beneficial effect of the experimental treatment despite high risk of bias in primary studies**<br><br>**Explanation:** judged when the abstract concluded that the intervention was beneficial while the included primary studies had high risk of bias (e.g., inadequate randomization, lack of blinding, incomplete outcome data), and this limitation was not acknowledged.

Misleading interpretation	(18) Conclusion formulating recommendations for clinical practice not supported by the findings<br><br>Explanation: judged present when strong clinical recommendation was given despite weak evidence.	(16) Conclusion claiming the beneficial effect of the experimental treatment despite reporting bias<br><br>Explanation: judged when the abstract presented the intervention as beneficial even though there was evidence of reporting bias (selective outcome reporting, publication bias, asymmetry in funnel plot), and this limitation was not acknowledged.
	(19) Conclusion claiming safety based on nonstatistically significant results with a wide confidence interval<br><br>Explanation: flagged when the absence of significance in harms was used to claim safety.	(17) Conclusion claiming the beneficial effect of the experimental treatment despite high heterogeneity<br><br>Explanation: judged when the abstract concluded in favor of the intervention despite substantial heterogeneity among included studies ($I^2 \geq 75\%$), without acknowledging its impact on the certainty or consistency of the evidence.
	(20) Conclusion ignoring the high risk of bias of the studies, the heterogeneity or the reporting bias (i.e., a low level of evidence) in the interpretation of the results<br><br>Explanation: marked when interpretation did not account for serious methodological flaws or high risk of bias presented.	(18) Ignoring that the review included different study design (e.g., controlled trial or observational studies<br><br>Explanation: judged when the abstract did not clearly state that the review synthesized evidence from more than one study design (e.g., RCTs and non-RCTs, or different types of observational studies), potentially misleading readers about the overall strength and level of evidence.
	(21) No or inadequate consideration of heterogeneity in results interpretation (i.e., no assessment of heterogeneity reported, claiming the absence of heterogeneity not supported by the data, claiming the beneficial effect of the treatment despite high heterogeneity, no downgrading the evidence in cases of high heterogeneity, interpreting nonstatistical significant results for the test of heterogeneity as an evidence of no heterogeneity etc)	

	**Explanation:** judged when heterogeneity test not reported or ignored despite high I^2 (≥75%).	
Inappropriate extrapolation	(22) No or inadequate consideration of the risk of bias of primary studies included in results interpretation (i.e., no reporting of the risk of bias of the primary studies, claiming the low risk of bias of studies included not supported by the data, no downgrading the evidence despite several high risk of bias studies)<br><br>**Explanation:** judged when risk of bias assessment of primary studies was absent, not incorporated into the interpretation, or when conclusions claimed "low risk of bias" despite several studies being rated as high or unclear risk. Spin was also recorded when authors failed to downgrade evidence certainty in the presence of multiple high-risk studies.	
	(23) No or inadequate consideration of reporting bias in results interpretation (i.e., no reporting of an assessment of reporting bias, claiming efficacy despite an evidence of reporting bias, claiming the absence of reporting bias not supported by the data, negative test result interpreted as absence of publication bias, use of the test without the condition of validity, inade quate interpretation of a funnel plot, etc)<br><br>**Explanation:** judged when assessment of reporting bias (e.g., funnel plot, Egger's test) was absent or misinterpreted. Spin was recorded when authors claimed "no publication bias" without presenting valid analyses, when a negative test result was interpreted as definite absence of bias, or when evidence of reporting bias was ignored while still concluding treatment efficacy.	
	(24) Inadequate extrapolation of the results from surrogate markers or specific outcome to the global improvement of the disease	(19) Conclusion extrapolating the review's findings to a different population or setting

Inappropriate extrapolation	**Explanation (dental context):** judged when conclusions were based on surrogate outcomes (e.g., marginal adaptation, color stability, surface roughness, microleakage, or in vitro wear) and extrapolated to overall clinical success or long-term survival of dental restorations, without supporting evidence from patient-centered or long-term clinical outcomes.	**Explanation (dental context):** judged when the abstract generalized results obtained in a specific population or clinical setting (adults, permanent teeth, single-center studies) to different groups or contexts (children, deciduous teeth, elderly patients, or general dental practice), without sufficient supporting evidence.
	**(25) Inadequate extrapolation of the results to a larger population, a larger setting or a wider set of interventions (e.g., from a specific rehabilitation program to all rehabilitation programs, to a specialized unit to a nonspecialized medical unit etc)**<br><br>**Explanation (dental context):** judged when conclusions generalized results from specific conditions (one tooth type, a particular clinical setting, or a single restorative material) to broader populations, settings, or interventions (extending findings from anterior teeth to all teeth, from in vitro studies to clinical effectiveness, or from one resin composite brand to all restorative materials) without sufficient supporting evidence.	**(20) Conclusion extrapolating the review's findings to a different intervention (i.e., claiming efficacy of one specific intervention although the review covers a class of several interventions)**<br><br>**Explanation (dental context):** judged when the abstract claimed the effectiveness of one specific restorative material or technique (a particular brand of composite resin, a specific adhesive strategy) even though the review synthesized evidence on a broader class of interventions (all resin composites, all adhesive systems, or all indirect restorations).
	**(26) Conclusion extrapolating the review's findings to a different population or setting**<br><br>**Explanation (dental context):** judged when authors generalized results obtained in specific populations or clinical settings (adults, permanent teeth, single-center studies) to different groups or contexts (children, deciduous teeth, elderly patients, or multicenter/general practice settings) without evidence supporting such extrapolation.	**(21) Conclusion extrapolating the review's findings from a surrogate marker or a specific outcome to the global improvement of the disease**<br><br>**Explanation (dental context):** judged when the abstract drew conclusions about overall clinical success, long-term survival, or patient-centered outcomes of restorative treatments based only on surrogate measures (microleakage, bond strength, marginal adaptation, color stability), without direct evidence from clinical endpoints.

Inappropriate extrapolation	**(27) Conclusion extrapolating the review's findings to a different intervention (i.e., claiming efficacy of one specific intervention although the review covers a class of several interventions)**<br><br>**Explanation:** judged when conclusions claimed the efficacy of one specific material or technique (a particular brand of composite resin, a specific adhesive system, or CAD/CAM resin blocks) although the review included a broader class of interventions (all resin composites, all adhesive strategies, or all indirect restorations).	
	**(28) Conclusion extrapolating the review's findings from a surrogate marker or a specific outcome to the global improvement of the disease**<br><br>**Explanation (dental context):** judged when conclusions were based on surrogate or specific outcomes (microleakage, bond strength, surface roughness, marginal discoloration) and extrapolated to overall clinical success, longevity, or patient-centered outcomes of restorative treatments, without direct evidence from long-term clinical data.	
