# Supplementary material for: Spin and reporting quality in systematic reviews of nonrandomized studies in restorative dentistry: a meta-research study
Source: Braz Oral Res. 2026 Nov 9;40:e094. doi: 10.1590/1807-3107bor-2026.vol40.094 (PMC13750809; doi:10.1590/1807-3107bor-2026.vol40.094)
Supplement: Supplementary file 7 [file 1807-3107-bor-40-e094-suppl07.pdf]

Supplementary Material 7. Presentation for ROBIS Results.

Review	Phase 2				Phase 3
	1.Study eligibility criteria	2. Identification and selection of studies	3. Data collection and study appraisal	4. Synthesis and findings	Risk Of Bias In The Review
Al-Dabbagh (2021)	Unclear	High	Low	Unclear	High
Albanesi et al (2016)	Low	Unclear	Low	Low	Low
Alenezi et al., (2024)	Unclear	Unclear	Unclear	Unclear	Unclear
Alqutaibi et al. (2024)	Low	Low	Low	High	High
Bhagwat et al (2025)	Low	Unclear	Low	Low	Unclear
Bresser et al (2023)	Low	Low	Low	Unclear	Unclear
Chantler et al (2024)	Low	Low	Unclear	Unclear	High
Dewan et al (2023)	Low	Low	Low	Unclear	Unclear
Dioguardi et al (2021)	Low	Unclear	Low	Low	Low
Dioguardi et al (2022)	Low	Unclear	Low	Unclear	Unclear
Fan et al (2025)	Low	Low	Low	Unclear	Unclear
Fathi et al (2022)	Unclear	High	Unclear	Unclear	High
Heintze et al (2022)	Unclear	High	Unclear	Unclear	High
Kassardjian et al (2016)	Unclear	Unclear	High	Unclear	Unclear
Kreulen et al. (1998)	Unclear	High	Unclear	High	Unclear
Lim et al (2023)	Low	Low	Low	Low	Low
Mendes et al (2022)	Low	Low	Low	Unclear	Unclear
Pjetursson et al (2004)	Unclear	Unclear	High	High	High
Pjetursson et al (2007)	Unclear	Low	Unclear	High	High
Ploumaki et al (2013)	Low	High	High	High	High
Sailer et al (2007)	High	Unclear	Unclear	High	High
Sailer et al (2018)	Low	Unclear	Unclear	Unclear	High
Thoma et al (2017)	Low	High	High	Unclear	Unclear
Thomas et al (2020)	Low	Low	Low	Low	Unclear
Vetromila et al (2020)	Low	Low	Low	Unclear	Unclear
Whang et al (2022)	Low	Low	Low	Low	Low
