# Supplementary material for: Spin and reporting quality in systematic reviews of nonrandomized studies in restorative dentistry: a meta-research study
Source: Braz Oral Res. 2026 Nov 9;40:e094. doi: 10.1590/1807-3107bor-2026.vol40.094 (PMC13750809; doi:10.1590/1807-3107bor-2026.vol40.094)
Supplement: Supplementary file 8 [file 1807-3107-bor-40-e094-suppl08.pdf]

Supplementary Material 8. Ordinal logistic regression analysis of potential predictors of spin levels in abstracts of systematic reviews with meta-analysis.

Predictors	Odds Ratio (Unadjusted)	p-value	Odds Ratio (Ajusted)	p-value
Year				
≥ 2020	1	0.694		
< 2020	1.39 (0.26 – 7.34)			
Origin				
America	1	0.568		
Asia	2.44 (0.15 – 39.58)			
Europe	2.68 (0.39 – 18.19)			
Compliance with Word Limit				
Yes	1	0.364		
No	2.26 (0.38 – 13.29)			
Distinction primary/secondary outcomes				
Yes	1	0.205		
No	3.11 (0.49 – 19.54)			
Journal Endorsement of ICMJE				
No	1	0.557		
Yes	1.80 (0.25 – 12.82)			
Journal Endorsement of PRISMA				
Yes	1	0.389		
No	2.05 (0.38 – 10.89)			
Funding source				
No funding	1	0.037		
Non-profit	3.22 (0.26 – 38.62)			
Non-reported	28.49 (1.86 – 434.49)			
Manuscript cites PRISMA				
Yes	1	0.073		
No	5.17 (0.81 – 32.71)			
Manuscript Open Access				
Yes	1	0.395		
No	2.23 (0.33 – 14.98)			
Structured Abstract				
Yes	1	0.993		

No	1.99 (0.01 – 0.01)	
Conflict of interest reported		
No	1	0.334
Yes	2.24 (0.17 – 6.12)	
Risk of bias assessment		
Yes	1	0.972
No	1.03 (0.25 – 6.)	
GRADE assessment		
Yes	1	0.557
No	1.80 (0.25 – 12.82)	
Registered Protocol		
Yes	1	0.465
No	1.82 (0.35 – 9.29)	
ROBIS tool		
Low	1	
Unclear	7.79 (0.46 – 129.82)	0.236
High	2.72 (0.16 – 45.28)	
