# Supplementary material for: Spin and reporting quality in systematic reviews of nonrandomized studies in restorative dentistry: a meta-research study
Source: Braz Oral Res. 2026 Nov 9;40:e094. doi: 10.1590/1807-3107bor-2026.vol40.094 (PMC13750809; doi:10.1590/1807-3107bor-2026.vol40.094)
Supplement: Supplementary file 9 [file 1807-3107-bor-40-e094-suppl09.pdf]

Supplementary Material 9. Sensitivity analysis of borderline associations after Bonferroni correction*.

Outcome	Predictor	Comparison	PR (Adjusted, 95% CI)	p-value	Bonferroni-adjusted p-value
**Spin occurrences in abstracts**	Funding source	Not reported vs no funding	2.10 (1.22–3.59)	0.004	0.016
**Spin occurrences in main text**	Publication year	< 2020 vs ≥ 2020	1.81 (1.18–2.07)	0.014	0.056
	Structured Abstract	No vs Yes	1.56 (1.18 – 2.07)	0.002	0.008
**Spin occurrences in main text**	Registered protocol	No vs Yes	1.68 (1.08–2.61)	0.019	0.076
