# Supplementary material for: Spin and reporting quality in systematic reviews of nonrandomized studies in restorative dentistry: a meta-research study
Source: Braz Oral Res. 2026 Nov 9;40:e094. doi: 10.1590/1807-3107bor-2026.vol40.094 (PMC13750809; doi:10.1590/1807-3107bor-2026.vol40.094)
Supplement: Supplementary file 10 [file 1807-3107-bor-40-e094-suppl10.pdf]

Supplementary Material 10. Percentage comparison of randomized and non-randomized studies that contain each spin item in the abstract and full-text (data from systematic reviews with randomized clinical trials by Sensever et al., 2022).

Description	Spin n (%)			
	Abstract		Full-text	
	SRs Randomized n= 49	SRs non-randomized n = 26	SRs Randomized n =49	SRs non-randomized n= 26
Conclusion contains recommendations for dental clinical practice not supported by the findings	8 (16.3)	6 (23.07%)	11 (22.4)	8 (30.76%)
Title claims or suggests a beneficial effect of restorative material or technique not supported by the findings	0 (0)	0 (0)	0 (0)	0 (0)
Selective reporting of or overemphasis on efficacy outcomes or analysis favoring the beneficial effect of one of restorative techniques or materials	8 (16.3)	2 (7.69%)	7 (14.3)	1 (3.84)
Omission of primary outcomes	1 (2.0)	1 (3.84)	0 (0)	2 (7.69%)
Selective reporting of positive results and omission of negative results of primary outcomes	2 (4.1)	1 (3.84)	0 (0)	0 (0)
Fail to report statistically nonsignificant primary outcomes	0 (0)	2 (7.69%)	0 (0)	0 (0)
Focus on statistically significant outcomes other than the primary	0 (0)	2 (7.69%)	0 (0)	1 (3.84)
Overenthusiastic interpretation of primary outcomes results as effective	10 (20.4)	2 (7.69%)	9 (18.4)	1 (3.84)
